# HRDPath: an explainable multi-model deep learning architecture for predicting homologous recombination deficiency from histopathology images

**DOI:** 10.1038/s41467-026-78430-y

**Published:** 2026-10-21

**Authors:** Chun-I Wu, Kalyan Banda, Rupali Arora, T. Rinda Soong, Marc Radke, Melanie Dillon, Serena Nik-Zainal, Elizabeth M. Swisher, Heba Z. Sailem

**Affiliations:** 1 School of Cancer and Pharmaceutical Sciences, Stamford St., Franklin Wilkins Building, King’s College London, London, UK; 2 Clinical Research Division, Fred Hutchinson Cancer Center, Seattle, WA USA; 3 University College London Hospital, London, UK; 4 Department of Pathology, University of Pittsburgh School of Medicine, Pittsburgh, PA USA; 5 Division of Gynecologic Oncology, University of Washington, Seattle, Washington, Seattle, WA USA; 6 MRC Cancer Unit, Hutchison/MRC Research Centre, Cambridge Biomedical Research Campus, Cambridge, UK; 7 King’s Institute for Artificial Intelligence, King’s College London, London, UK

**Keywords:** Biomarkers, Computational biology and bioinformatics

## Abstract

Homologous recombination deficiency (HRD) is a critical biomarker for guiding treatment decisions in high-grade serous tubo-ovarian carcinoma (HGSOC), a cancer with few reliable biomarkers. However, existing genomics-based tests for HRD are variable, expensive, and time-consuming. We develop HRDPath, a patient-level deep learning architecture that predicts HRD status from whole slide images, requiring no additional molecular testing. HRDPath is validated across three cohorts of female patients and achieves an AUC of 0.846, significantly outperforming previously reported results in HGSOC. By incorporating multiple slides per patient and targeted optimization strategies, we strengthen the model clinical utility, with both specificity and sensitivity reaching 0.90. Our interpretability pipeline enhances model trustworthiness and reveals important clinical and biological insights into HRD-positive cancers, highlighting the associated morphological and pathological changes at the cellular and tissue levels. HRDPath provides a foundation for an accessible and scalable digital biomarker to improve treatment stratification in ovarian cancer globally.

## Abstract (web summary)

Genomic-based tests for Homologous recombination deficiency (HRD) are variable, expensive, and time-consuming. Here, the authors find that the multi-model transformer-based method HRDPath could be used as a digital biomarker to improve ovarian cancer diagnosis and therapy selection.’

## Introduction

High-grade serous tubo-ovarian carcinoma (HGSOC) is a highly aggressive malignancy with a paucity of available biomarkers for therapeutic intervention. HGSOC exhibiting homologous recombination deficiency (HRD) demonstrates marked sensitivity to targeted treatments such as platinum-based chemotherapy and Poly ADP-Ribose polymerase (PARP) inhibitors^1–3^. Specifically, PARP inhibitors demonstrate synthetic lethality in HRD-positive cancers by inhibiting the repair of damaged DNA, significantly extending progression-free survival and improving long-term outcomes in patients with HRD-positive ovarian cancer^4–6^. Given the significant relationship between HRD status and patient treatment outcomes, the identification of HRD is crucial for precision therapy.

Existing HRD tests analyze both mutations in DNA repair genes, such as *BRCA1/2*, and genomic instability. Examples include myChoice^7^, which uses *BRCA1/2* alterations and a combined genomic instability score^8,9^; FoundationOne^10^, which assesses *BRCA1/2* alterations alongside loss of heterozygosity and other genomic changes; and HRDetect, a widely accepted tool for HRD detection that has not been adopted clinically due to its dependence on costly whole-genome sequencing^11,12^. However, current HRD diagnostic tests, such as myChoice, predict a much less robust response to PARP inhibitors in *BRCA1/2*-wild-type cancers compared to germline or somatic *BRCA1/2* mutations^4,6,9,13^. Moreover, these tests may be less accessible in regions of the world with limited resources due to their high cost and reliance on advanced genomic technologies.

We hypothesized that using deep learning to predict HRD from hematoxylin and eosin-stained (H&E) whole-slide image (WSIs) has the potential to become a widely accessible biomarker detection tool, as H&E staining is routinely used in clinical practice worldwide and offers a cost-effective cancer profiling approach. Indeed, previous studies have shown progress in predicting *BRCA1/2* mutations and HRD status from H&E images in breast cancer^14^ but have showed limited performance in ovarian cancer with no reported data on method specificity or sensitivity^15^. Additionally, the application of modern slide-based weakly supervised learning approaches where the model takes the entire WSI as input and predicts clinically relevant labels such as grade, subtypes or genetic phenotypes remains lacking in the context of HRD prediction in HGSOC^16–18^. Therefore, a robust deep learning model with clinically relevant performance, robust interpretability, and generalizability has yet to be developed for HGSOC. Moreover, how HRD influences tumor architecture and heterogeneity remains underexplored.

In this work, we develop a multi-model transformer-based method, HRDPath, for cost-effective HRD detection from gigabyte-sized WSIs of primary or metastatic HGSOC tumors. We utilize a weakly supervised learning approach to identify image regions in WSIs that can consistently distinguish patterns and image regions associated with HRD-positive (HRD+) from HRD-negative (HRD−) cancers. A key feature of HRDPath is its patient-level analysis, aggregating data from multiple slides, without requiring precise anatomical or neoplastic region localization. HRDPath achieves up to 0.846 AUC with 0.692 sensitivity and 0.938 specificity in detecting HRD in an test dataset and achieves comparable performance on two publicly available datasets. Moreover, we propose several strategies to enhance the translational potential of HRDPath and increase confidence in its predictions. To better understand HRDPath performance and the pathological manifestation of HRD in HGSOC and support trust in model predictions, we develop explainability approaches to assess regions that receive high attention from the model based on deep-learning features, cancer cell morphology, and tumor composition^10–13^. These analyses consistently reveal that HRD+ HGSOC exhibit distinct morphologies, including polymorphic nuclei. Finally, we demonstrate that HRDPath predictions correlate with patient outcomes. Our work demonstrates state-of-the-art performance in ovarian cancer and provides significant insights into the pathological changes associated with HRD.

## Results

### Training and evaluation datasets

**Fig. 1:**
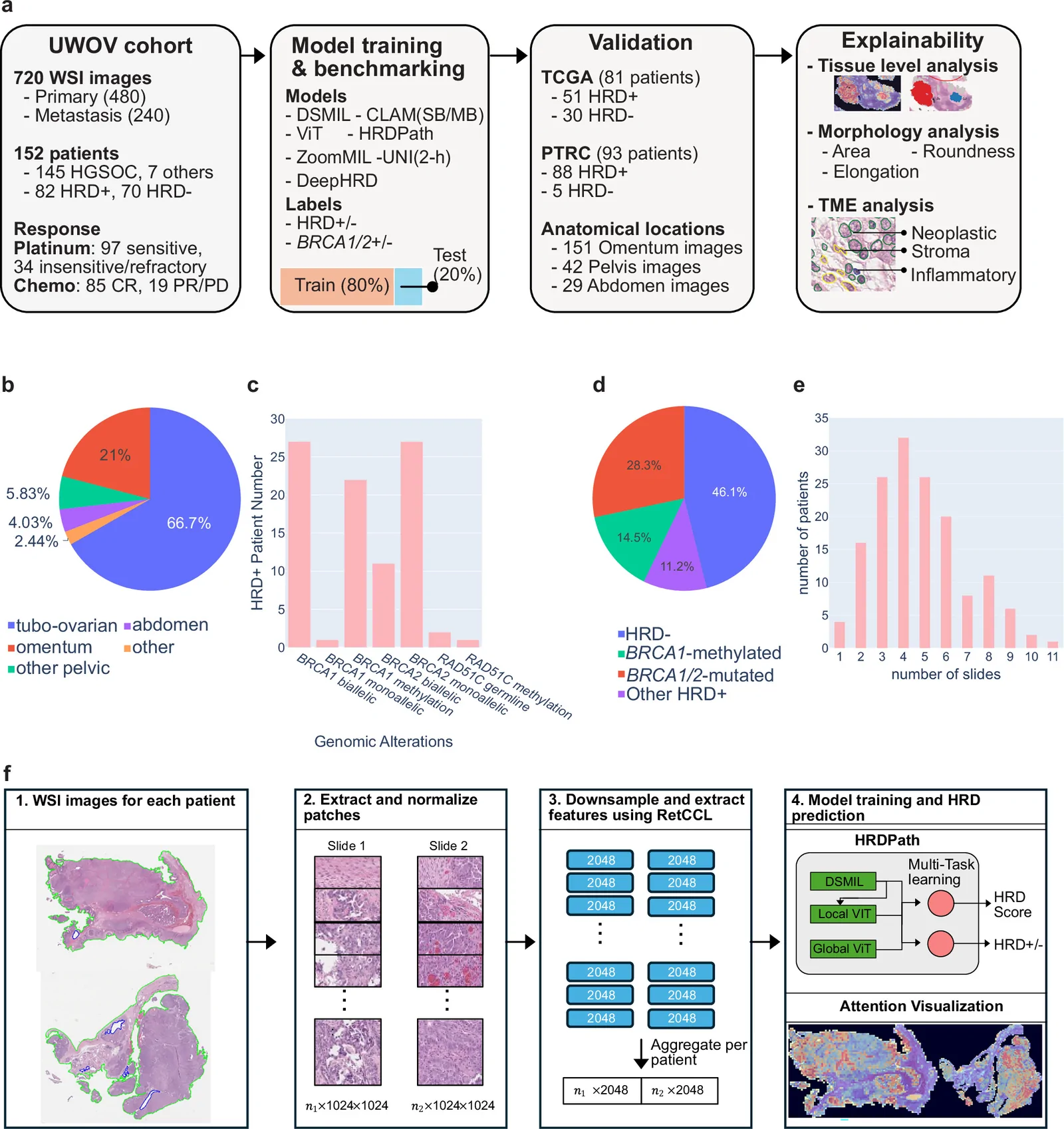
Overview of the HRDPath workflow and study design. **a** Schematic of the experimental design and dataset composition. HRD homologous recombination deficiency, HGSOC high-grade serous ovarian cancer, WSI whole-slide image, CR complete response, PR/PD partial response/progressive disease, DSMIL Dual-Stream Multiple Instance Learning, CLAM Clustering-constrained Attention Multiple Instance Learning, ViT Vision Transformer, RetCCL Retrieval with Clustering-guided Contrastive Learning, TME Tumor microenvironment. **b** Distribution of slides by anatomical location. **c** Genomic stratification of HRD+ patients, including *BRCA1/2* mutations, *BRCA1* methylation, and *RAD51C* alterations. **d** Proportional distribution of *BRCA1/2*-associated alterations in HRD+ cases. **e** Histogram showing the number of slides per patient in the UWOV cohort. **f** HRDPath pipeline, including patch extraction, feature embedding via RetCCL, model training, and prediction (ni: number of cleaned patches for slide index:i). Source data are provided as a Source Data file.

We utilized an in-house dataset, UWOV, with 152 patients (145 HGSOC and 7 other ovarian cancer patients) as well as two publicly available datasets: TCGAOV (81 HGSOC patients with 82 diagnostic WSIs) and PTRC (93 HGSOC patients) (Fig. 1a and Methods). For our model development, we focused on the UWOV cohort due to its high-quality, large sample size, and the comprehensive information from multiple slides of different anatomical locations per patient, and used TCGAOV and PTRC as independent validation sets where we only used diagnostic slides from TCGA. The UWOV dataset consists of 720 diagnostic whole tissue section slides from primary debulking resections of HGSOC. The specimens encompassed fallopian tube or ovary (tubo-ovarian: *n* = 480) and extra-ovarian anatomic locations: omentum (*n* = 151), abdomen (*n* = 29), Other Pelvic (*n* = 42), and other locations (*n* = 18) (Fig. 1b). HRDetect was used to generate ground truth HRD status for the UWOV dataset^19^ (“Methods”). 70 patients were classified as HRD−, while 82 were classified as HRD+ . These cancers were also assessed for HRD-related genomic alterations, including *BRCA1* (32.9%), *BRCA*2 (25.7%), and *RAD51C* (2%) mutations (Fig. 1c and Methods). Methylation alterations were also assessed, where among the HRD+ cancers, neoplasms from 65 patients (79.3%) harbored either *BRCA1/2*-associated mutations o*r BRCA1* methylation (Fig. 1d). We adopted a patient-level training framework, where multiple WSIs from each patient were combined into a single input (Fig. 1e and “Methods”). In the training stage, we considered only patients with diagnostic slides from the primary fallopian tube or ovary (tubo-ovarian), or omentum, as it is the most common metastatic site (146 patients with 631 slide images). The number of slides per patient was comparable between HRD+ and HRD− groups (Overall mean: 4.3 ± 2.19; HRD−: 4.99 ± 2.07; HRD+: 4.52 ± 2.12), where Fig. 1e shows the distribution of the number of WSIs per patient across the cohort. To ensure that HRDPath learns features associated with HRD, rather than patient-specific patterns, all slides from a single patient were assigned exclusively to either the training or testing set. Specifically, we used data from 117 female patients (80%) with 493 slides (tubo-ovarian: 377; omentum: 116) for training, and 29 female patients (20%) with 138 slides (tubo-ovarian: 103; omentum: 35) for validating the model (Supplementary Table 1).

Given the large size of WSI images, we extracted smaller patches that were processed following the CLAM^20^ processing pipeline and encoded using a histopathology-pretrained RetCCL^21^ feature extractor. We also performed color normalization to ensure transferability across datasets (“Methods”). This pipeline enables the efficient handling of gigapixel images into a series of deep learning-based feature vectors that are then used for the subsequent classification of HRD status.

### HRDPath development

To predict HRD status from WSIs, we developed HRDPath, a weakly supervised learning approach (Fig. 1f). HRDPath is an end-to-end model that overcomes limitations of previous approaches for HRD prediction as it operates without the need for tumor segmentation and has been validated on two independent benchmarking unseen datasets (Tables 1–2 and Supplementary Note 1). HRDPath introduces several key features, including multi-task learning for predicting binary HRD status as well as continuous HRD score, resulting in more comprehensive evaluations. Additionally, it introduces patient-level learning that increases confidence in prediction by aggregating features from multiple WSIs (Supplementary Fig. 1a–f). Moreover, HRDPath adopts an ensemble approach that combines multiple models with different learning strategies, employing a Vision Transformer (ViT)^22^ and Dual-Stream Multiple Instance Learning^23^ (DSMIL models) (Box 1; Methods). Specifically, HRDPath utilizes DSMIL, which identifies and aggregates information from key image patches and analyzes how they relate to the remaining patches. Second, to capture local and global information, HRDPath utilizes a ViT model that uses a self-attention mechanism to learn the most relevant regions in WSI images.

**Table 1:** Comparison of HRDPath against other existing studies on image-based HRD prediction in Ovarian Cancer (OC)

Study/Method	Loeffler et al.^35^	Zhang et al.^36^	Bergstrom et al.^24^ (DeepHRD)	HRDPath (Ours)
Cancer types considered	Breast (main focus) OC (train and validate) And 7 others	OC	Breast (main focus) OC (validation only)	OC
Performance on OC (training dataset)	AUC: 0.63 Specificity: NP Sensitivity: NP	AUC: 0.769 Specificity: NP Sensitivity: NP	Breast cancer AUC: 0.77–0.85 HGSOC: NP	AUC: 0.846 Specificity: 0.938 Sensitivity :0.692
Performance on OC (independent dataset)	NP	NP	NP	Few-shot on PTRC & TCGAOV
Number of OC WSIs	TCGAOV (*n* = 520 diagnostic and non-diagnostic WSIs)	MSKCC (*n* = 205), Zhongda (*n* = 22)	TCGAOV subset (*n* = 459 diagnostic and non-diagnostic WSIs) + MSKCC (*n* = 141 WSIs)	UWOV (*n* = 720) PTRC (*n* = 198) TCGAOV (82 diagnostic WSIs from 81 patients)
Model details	Slide-level attention-based MIL	Two-stages: 1) U-Net++ segmentation 2) random forest classifier	Patch-level Ensemble ResNet18	- End-to-end deep learning - Multi-task - Patient-level
Prediction of continuous HRD score	No	No	No	Yes (average correlation of 0.653)
Multi-slide aggregation	No	No	No	Yes (Transformer-based)
Assessment of intra-patient consistency	NP	NP	NP	Yes
HRD scores considered	myChoice	myChoice	myChoice or (GIS > 40 or *BRCA1/2-mutated)*	- HRDetect - myChoice
Metastases investigation	No	No	No	Yes (Omentum, abdomen, other pelvic sites)
Explainability	Partially (showing highlighted tumor regions)	Partially (showing highlighted tumor regions)	Partially (showing highlighted tumor regions)	Yes: Neoplastic cell morphology, Tumor microenvironment Clustering of model attention
Limitations	- Requires large training datasets; - Low ovarian performance. - Limited interpretability	- Not end-to-end, - Need segmentation of tumor region; - No direct inference validation	- Patch-level classification - Only select top 5 patches and average their features across an ensemble of models - Indirect ovarian validation based on correlation with survival	Main evaluation on HRDetect

*NP* Not performed/provided.

**Table 2:** HRDPath performance and benchmarking against widely-used architectures. ++ indicates that all models are adapted with our patient-level and multiple task learning approach to ensure objective assessment

Model	AUC	Sensitivity	Specificity
ViT++	0.707	0.385	0.813
UNI++	0.731	0.769	0.563
CLAM-MB++	0.745	0.692	0.750
CLAM-SB++	0.750	0.615	0.875
ZoomMIL++	0.808	0.615	0.813
DSMIL++	0.808	0.538	0.875
DeepHRD++ (*k* = 5, *n* = 3)	0.827	0.769	0.750
HRDPath-Uni-Task learning (UTL)	0.760	0.846	0.688
HRDPath-Multi-task learning (MTL) (ours)	0.846	0.692	0.938

Box 1 HRDPath training algorithm

**Figure:**
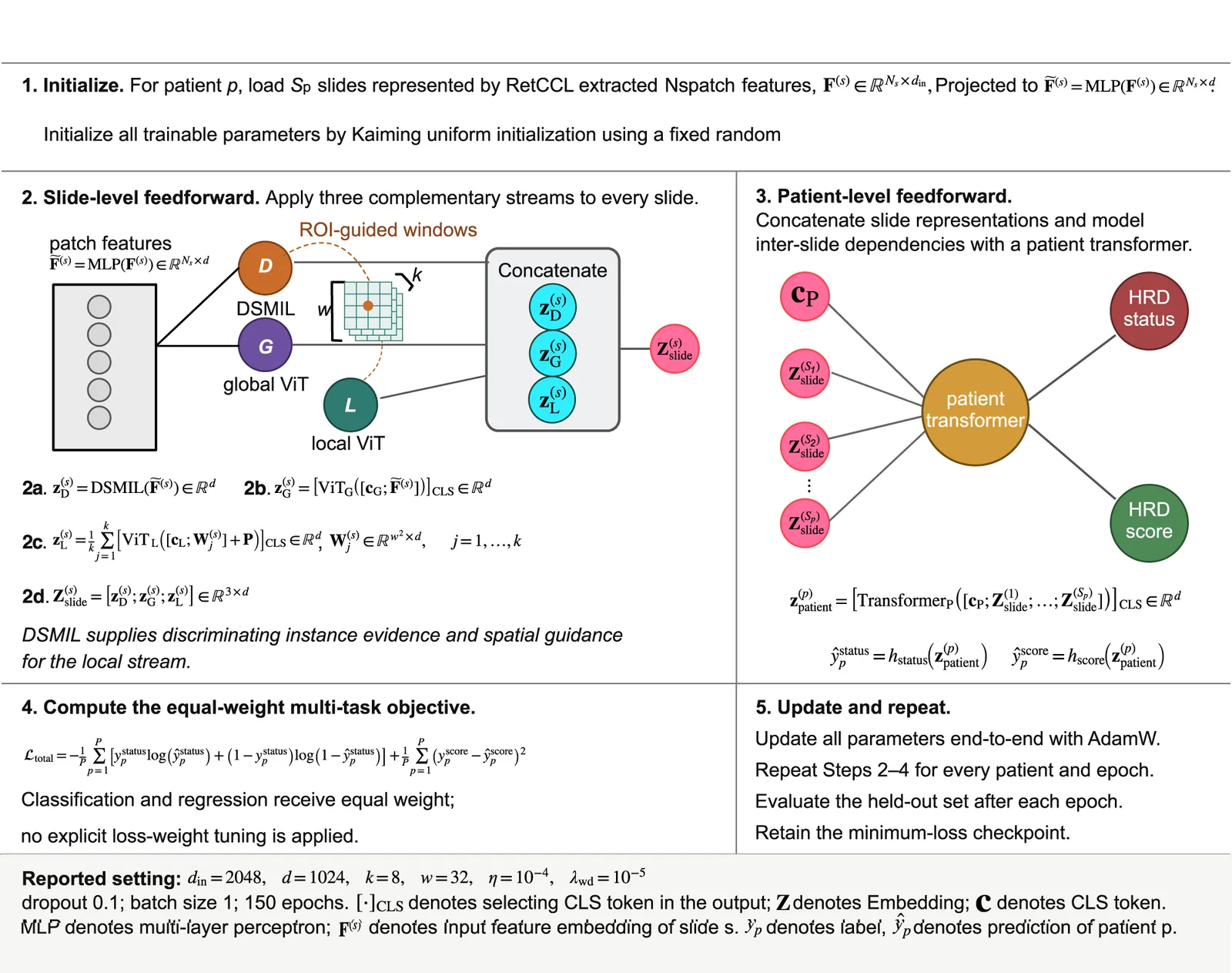

### HRDPath accurately predicts HRD status from histology images

**Fig. 2:**
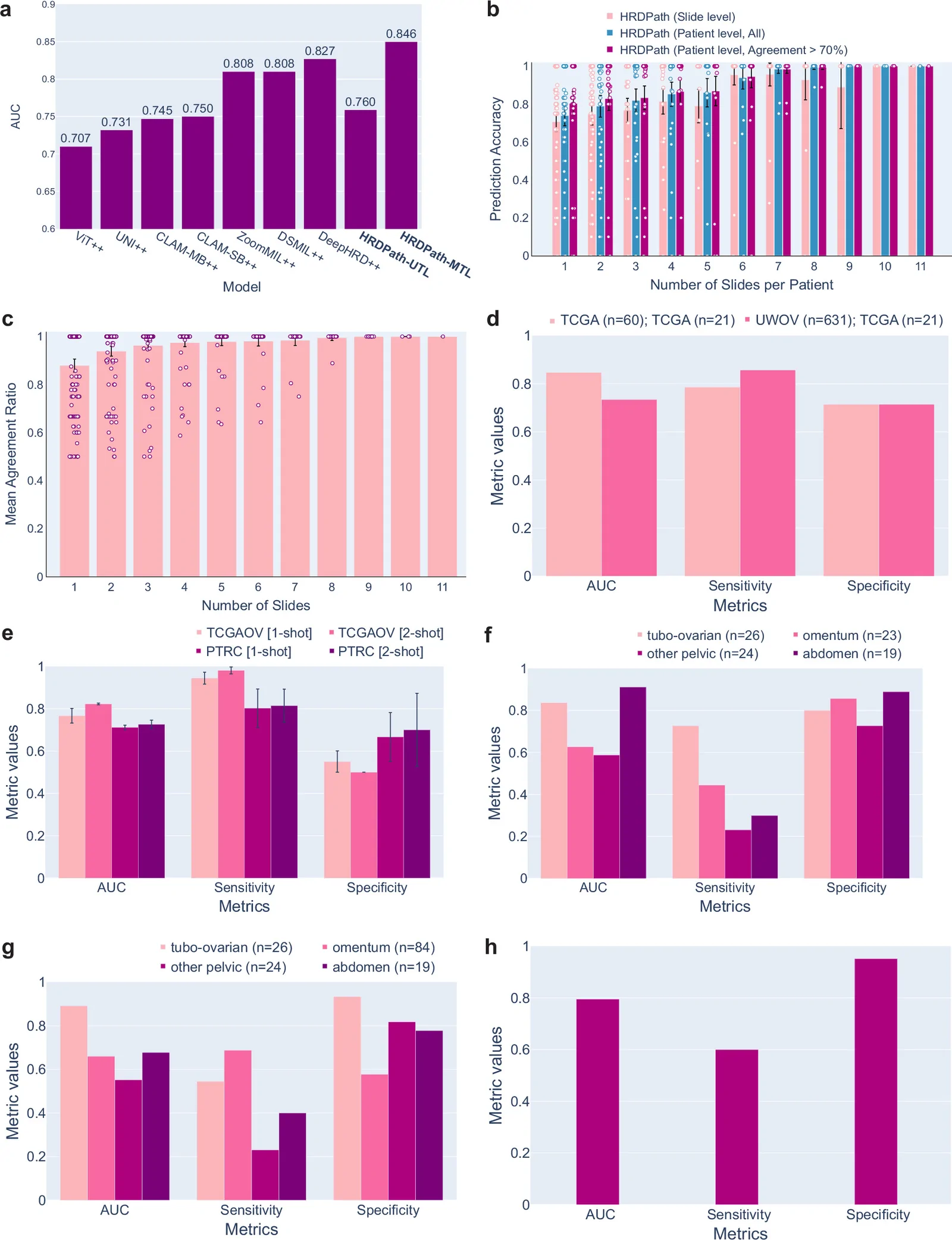
HRDPath accurately predicts HRD across datasets and anatomical locations. **a** UWOV benchmark results (*n* = 138 WSIs from 29 test female patients aged 38–83). All methods were adapted using our patient-level multi-task learning strategy (++). **b** Prediction accuracy across 1–11 slides per patient as input to HRDPath (720 WSIs from 152 female patients aged 18–86) was grouped by patient and evaluated by majority voting on all WSI combinations. Per-patient agreement ratio across combinations with ground truth shown as dots. Error bars denote 95% confidence intervals of the mean. **c** Mean agreement across slide combinations per patient for HRDPath (*n* = 720 WSIs from 152 female patients aged 18–86). Bars show mean per-patient agreement ratio; dots indicate per-patient agreement ratios; error bars denote 95% confidence intervals of the mean. **d** Generalizability results on the TCGAOV test set (21 WSIs from 21 female patients aged 40–78). TCGAOV model training used 61 WSIs from 60 patients. **e** Few-shot validation with Youden-optimal thresholds in the TCGAOV (*n* = 82 WSIs from 81 female patients aged 37–85) and PTRC-HGSOC (*n* = 198 WSIs from 93 female patients aged 36–87) datasets (Supplementary Table 7). Bars show mean metric values across folds; dots indicate fold-level values; error bars denote standard deviation across folds (*n* = 3 folds per dataset per shot setting). **f** Performance across anatomical locations in the test set for HRDPath pretrained on both tubo-ovarian and omental slides (tubo-ovarian: *n* = 103 WSIs from 26 female patients aged 38–83; omentum: *n* = 35 WSIs from 23 female patients aged 44–81; other pelvic: *n* = 42 WSIs from 24 female patients aged 43–83; abdomen: *n* = 29 WSIs from 19 female patients aged 39–82). **g** Performance of HRDPath-primary trained only on tubo-ovarian slides across anatomical locations (tubo-ovarian: *n* = 103 WSIs from 26 female patients aged 38–83; omentum: *n* = 151 WSIs from 84 female patients aged 18-86; other pelvic: *n* = 42 WSIs from 24 female patients aged 43–83; abdomen: *n* = 29 WSIs from 19 female patients aged 39–82). **h** Prediction of *BRCA1/2*-associated alterations, demonstrating transferability to related classification tasks (*n* = 160 WSIs from 31 female patients aged 38-83). No statistical hypothesis tests were performed in any panel; therefore, no multiple-comparison adjustment was applied. UTL, uni-task learning; MTL, multi-task learning. Source data are provided as a Source Data file.

When using the DSMIL or ViT model alone for predicting HRD, DSMIL performed better than ViT, with an AUC of 0.808 with 0.875 specificity and 0.538 sensitivity (Fig. 2a and Table 2). This motivated us to develop HRDPath that integrates both models resulting in significant improvement in performance on the test dataset with AUC of 0.846, specificity of 0.938, and sensitivity of 0.692 (Fig. 2a and Supplementary Fig. 2a–i). Incorporating a multi-task learning objective proved essential for achieving superior specificity with an absolute improvement of 0.250 and a higher overall performance, reflected by a 0.087 gain in AUC. However, this enhancement came at the cost of reduced sensitivity (Table 2). Importantly, HRDPath achieved good correlation with HRD score (*r* = 0.653–0.81; Supplementary Fig. 2j–l). These benchmark results remained consistent when the models were pretrained on TCGAOV, further supporting the robustness of HRDPath (Supplementary Table 2).

To evaluate the discriminative capacity of the continuous HRD probabilities, Area Under the Receiver Operating Characteristic (AUROC) analysis was performed (Supplementary Fig. 2a–i). The HRDPath model, utilizing multi-task learning (MTL), maintained robust overall ranking ability across the continuous score spectrum. Notably, the ROC curve demonstrates a favorable trade-off pattern in the high-specificity regime, achieving a True Positive Rate of approximately 0.7–0.8 while maintaining a False Positive Rate below 0.2. However, pushing the model to maximum sensitivity requires a substantial compromise in specificity, a pattern consistent with the score dispersion observed among certain HRDetect-positive cases (Supplementary Fig. 2j). These continuous probability metrics confirm that while the fixed 0.5 threshold provides a balanced baseline, the continuous scores capture a meaningful clinical signal that could support threshold optimization for specific sensitivity-driven or specificity-driven clinical objectives.

The performance of HRDPath was generally robust to the proportion of neoplastic cells and cellularity (Supplementary Fig. 3a). This suggests that genomic alterations leading to HRD are associated with consistent changes in tissue architecture and visual patterns detectable from H&E images.

### Evaluation of HRDPath consistency to support translation

As WSIs from multiple tumor locations are often acquired in HGSOC, we sought to evaluate HRDPath consistency when multiple slides are used per patient. We found that HRDPath predictions are generally consistent across different slides from the same patient, and that integrating features from three or more slides can significantly improve model accuracy and consistency (Fig. 2b–c and “Methods”). Importantly, when six or more slides were used, accuracy exceeded 0.950 with 0.910 sensitivity and 1.000 specificity (Fig. 2b and Supplementary Table 3). To further assess whether anatomical diversity contributes to model performance beyond slide count, we evaluated all enumerated slide combinations at fixed sampling depths (two or three slides per patient), stratified by the number of primary and metastatic WSIs, and diverse anatomy composition. When at least two primary-site slides were included, sensitivity and specificity remained high and stable regardless of whether additional slides originated from the primary site or other anatomical locations, indicating no independent performance gain from anatomical diversity under adequate primary sampling (0.906–0.938 sensitivity; 0.896–0.938 specificity; Supplementary Table 4). In contrast, when primary sampling was limited to a single slide, sensitivity decreased while specificity remained high (0.769–0.869 sensitivity; 0.941–1.000 specificity; Supplementary Table 4). This is likely due to the fact that our training cohort is currently under-represented in metastatic samples, which are limited to omental sites and constitute only 23% of the total training data. When restricting analysis to patients sampled from three distinct anatomical locations, HRDPath achieved a sensitivity of 1.000 and a specificity of 1.000. However, we note that this analysis is based on data from only 13 patients and needs to be expanded in future studies (Supplementary Table 4). Interestingly, we found that Pearson correlation between continuous HRD score and HRDPath-predicted score increased from 0.65 to 0.81 when more primary slides were considered (Supplementary Fig. 2k, l), suggesting that this approach might be more appropriate to mitigate tumor heterogeneity. This pattern remained consistent across the entire HRD score range, with no specific HRD score values linked to discordant cases. This patient-level aggregation strategy can also be used to derive a confidence score for model predictions by passing individual WSIs through the model and considering the prediction reliable only when at least 70% of slides yield consistent results. Collectively, these findings support the translational potential of HRDPath and underscore the utility of our patient-level approach in increasing the robustness and trustworthiness of model predictions.

### Benchmarking of HRDPath

To rigorously evaluate HRDPath, we benchmarked its performance against widely used and recent deep learning models in histopathology using the same patient cohort. Our evaluation included HRD-specific models and state-of-the-art methods with different learning strategies. These methods include the patch-based DeepHRD^24^, multi-scale ZoomMIL^25^ and feature extractions using the UNI foundation model^26^. For fair comparison of HRDPath architecture contribution, we incorporated the patient-level and multi-task learning approach (denoted by ++) as we found that it significantly improves the performance of these models (Supplementary Table 5). Moreover, all models were trained and tested using the same training/testing split and a fixed 0.5 probability threshold for classification. We also included the baseline models of HRDPath: Vision Transformers (ViT++)^26^ and DSMIL++^27^. We considered the widely used CLustering-constrained-Attention Multiple-instance learning (CLAM-SB++ and CLAM-MB++), which are both popular multiple instance learning approaches specifically designed for whole slide image analysis. HRDPath outperformed the ResNet-based DeepHRD++, particularly in specificity in UWOV, with an absolute improvement of 0.188 even when both models were trained using our multi-task, patient-level strategy (Table 2 and Fig. 2a). It also outperformed the widely used CLAM-SB++ and its variant CLAM-MB++ models in predicting HRD with absolute improvements of 0.096 in AUC, 0.063 in specificity, and 0.077 in sensitivity for CLAM-SB++ (Table 2). Moreover, HRDPath outperformed ZoomMIL++, a hierarchical model that integrates features extracted at different image resolutions. Surprisingly, we observe that using the foundation model UNI++ as a feature extractor results in significantly lower performance, which could be due to a more complex embedding (Table 2, Supplementary Note 2). These results show that HRDPath consistently outperforms other approaches, supporting its robustness and potential clinical applicability.

### Generalizability of HRDPath

We validated HRDPath on publicly available HGSOC data from TCGA and PTRC. In both TCGA and PTRC, HRD was defined based on myChoice method^27^ where neoplasms were classified as HRD+ if the sum of LOH, LST, and TAI was greater than 42 (Methods). Unlike UWOV, the TCGA cohort comprises 82 diagnostic WSIs from 81 patients (51 HRD+ and 30 HRD−); 80 patients contributed one WSI each and one patient contributed two WSIs^28^. PTRC-HGSOC comprises 198 slides (106 tubo-ovary and 92 omentum) from 93 patients from different hospitals with a highly imbalanced HRD distribution (88 HRD+ and 5 HRD− patients). Despite the small size of the TCGA dataset, HRDPath achieved comparable performance in predicting HRD status when trained on TCGA, consistently outperforming other models, achieving 0.847, 0.714, and 0.786 in AUC, specificity, and sensitivity, respectively (Fig. 2d; Supplementary Fig. 3b; and Supplementary Table 6). These results confirm that HRD status defined based on different HRD assays can be robustly predicted from H&E images.

To further confirm HRDPath generalizability, we evaluated its performance on independent cohorts using few-shot learning, images of metastatic neoplasms, and classification of *BRCA1/2* alterations (Supplementary Notes 3 and 4). We first employed 3-fold few-shot learning to determine whether HRDPath could be applied without significant finetuning. After fine-tuning with 1–2 example images per class, HRDPath achieved up to 0.823 AUC, 0.700 specificity, and 0.981 sensitivity in the TCGAOV and PTRC-HGSOC datasets (Fig. 2e and Supplementary Table 7). Color normalization significantly improved HRDPath generalizability across datasets (Supplementary Fig. 3c and Supplementary Table 8). We next assessed performance across metastatic sites. HRDPath showed good performance in predicting HRD from abdominal metastatic WSIs despite no abdominal samples being included in the training set (Fig. 2f; Supplementary Table 9 and Supplementary Note 3). Performance was lower in Other Pelvic samples, particularly when HRDPath was trained on primary-site images alone (Fig. 2f, g and Supplementary Table 10). Finally, HRDPath-BRCA classified *BRCA1/2*-associated alterations, including *BRCA1* methylation, with an AUC of 0.800, specificity of 0.952, and sensitivity of 0.600 (Fig. 2h; Supplementary Fig. 3d; Supplementary Table 11; Supplementary Note 4; “Methods”). These findings highlight the robustness and generalizability of HRDPath, suggest concordance between HRDetect and myChoice scores, and demonstrate HRD prediction with as few as one sample per class, a key step toward clinical translation.

### Ablation analysis of HRDPath components

Having previously established the baseline performance of the individual ViT and DSMIL modules that comprise HRDPath, we conducted a series of multi-model variants under identical experimental settings to elucidate the contribution of different components (Table 3). Integrating global and instance-level representations (ViT + DSMIL) improved sensitivity and specificity balance but did not consistently enhance overall AUC, suggesting that fusion of global and instance features alone is not sufficient. Adding local window construction (LWC) without absolute positional encoding (APE) increased the observed AUC from 0.745 to 0.769, although performance remained below that of the complete model. While using LWC and APE together in the final HRDPath model, the observed AUC increased to 0.846. We attribute this gain to the fact that local window construction, together with APE, imposes a CNN-like inductive bias by restricting attention to spatially proximate regions and explicitly encoding relative spatial relationships. This localized inductive structure enhances the model’s ability to capture spatially coherent morphological patterns that are critical for HRD prediction. These findings demonstrate that HRDPath’s performance improvements arise from the synergistic interaction between global morphology, discriminative instance selection, and CNN-inspired local spatial inductive bias, rather than from a single architectural component.

**Table 3:** Ablation study of HRDPath model components: local window construction (LWC), with and without absolute positional encoding (APE)

Model	AUC	Sensitivity	Specificity
ViT++	0.707	0.385	0.813
DSMIL++	0.808	0.538	0.875
HRDPath-Multi-task (ViT + DSMIL)	0.745	0.615	0.938
HRDPath-Multi-task (ViT + DSMIL + LWC)	0.769	0.615	0.938
HRDPath-Multi-task (ViT + DSMIL + LWC + APE) (our final model)	0.846	0.692	0.938

### Characterization of morphological changes identified by HRDPath

**Fig. 3:**
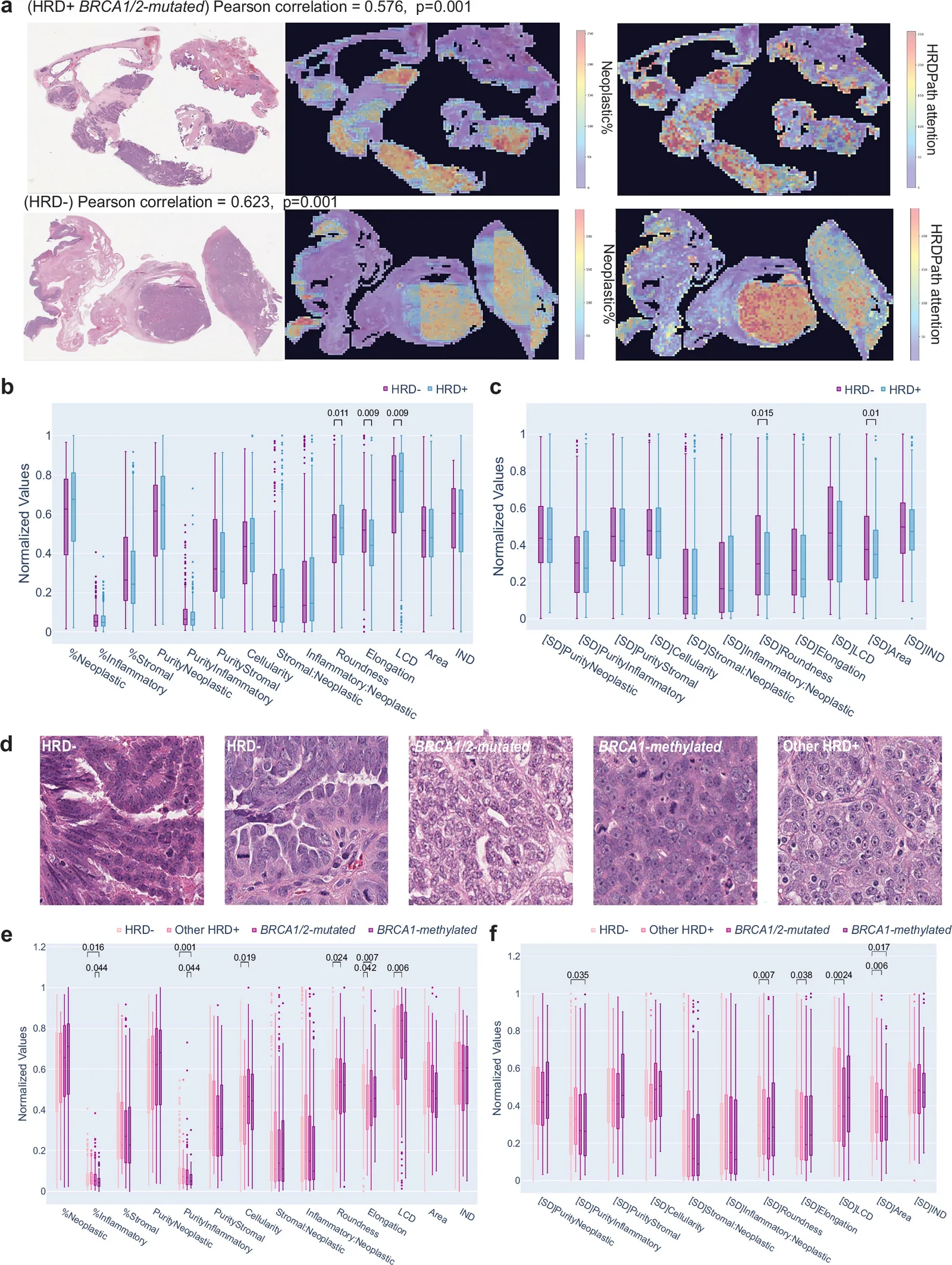
Characterization of morphological differences across HRD status and genomic alterations. **a** Example WSI images of HRD+/− neoplasms (left), corresponding neoplastic proportion heatmaps (middle), and HRDPath attention heatmaps (right). For **a**, Pearson correlation coefficients were calculated between patch-wise HRDPath attention and neoplastic-cell proportion using two-sided tests; no multiple-comparison adjustment was applied to these illustrative example-slide analyses. **b**, **e**. Distribution of average nuclear morphology per patient across HRD status (HRD−: 349 WSIs from 70 female patients aged 18–86; HRD+: 371 WSIs from 82 female patients aged 37–85) (**b**) and genomic alterations (HRD−: 349 WSIs from 70 patients aged 18–86; Other HRD+: 75 WSIs from 17 patients aged 39–77; *BRCA1/2*-mutated: 197 WSIs from 43 female patients aged 39–82; *BRCA1-*methylated: 99 WSIs from 22 female patients aged 37–85) (**e**). Center line, median; box bounds, 25th and 75th percentiles; whiskers, 1.5 × IQR; points beyond whiskers, outliers. **c**, **f**. Distribution of the standard deviation of nuclear morphology per female patient across HRD status (HRD−: 349 WSIs from 70 female patients aged 18–86; HRD+: 371 WSIs from 82 female patients aged 37–85) (**c**) and genomic alterations (HRD−: 349 WSIs from 70 patients aged 18–86; Other HRD+: 75 WSIs from 17 patients aged 39–77; *BRCA1/2*-mutated: 197 WSIs from 43 female patients aged 39–82; *BRCA1*-methylated: 99 WSIs from 22 female patients aged 37–85) (**f**), with box plots defined as in **b**, **e**. **d** Example regions across genomic alterations. *P* values in **b**, **c**, **e**, **f** were calculated using two-sided t-tests with FDR correction; only comparisons with corrected *q* < 0.05 are shown (values of *q* < 0.001 are rounded to 0.001). Source data are provided as a Source Data file.

To increase the confidence in the reliability of HRDPath predictions, we analyzed tissue architecture to explain HRDPath predictions. Although HRDPath did not receive any information on neoplastic regions, it successfully identified that visual patterns associated with HRD were generally located in neoplastic regions (average Pearson correlation between neoplastic proportion and model attention = 0.34 and Fig. 3a).

Using the HoVer-NeXt^29^ model we performed single-cell morphological analysis on 720 slides, while restricting the analysis to regions that received high attention (≥0.7) from HRDPath. We considered neoplastic, inflammatory, and stromal cell types as these were the most accurately predicted by the model as evaluated by a pathologist (“Methods”). We computed various features to quantify tumor composition, including proportion of different cell types, cellularity, cell purity, which measures the extent to which a single cell type dominates a specific image region (i.e., patch), and the ratio between different cell types which has been reported to be associated with clinical outcomes in HGSOC^30^. Additionally, we measured the mean and standard deviation (SD) of various morphological features of neoplastic nuclei, including roundness, elongation, area, local cell density (LCD), and immune-to-neoplastic cell distance (IND) (“Methods”).

HRD−+ HGSOC showed a higher proportion of neoplastic cells within tumor-associated regions compared with HRD− tumors. These differences were not due to variations in the overall neoplastic proportion between HRD+ and HRD− cancers, as no FDR-significant difference was observed when considering the entire WSI (Supplementary Fig. 3a). Compared with HRD− tumors, HRD+ tumors were characterized by significantly increased nuclear roundness (*q* = 0.011, Cohen’s *d* = 0.236; Fig. 3b), reduced nuclear elongation (*q* = 0.009, *d* = −0.262; Fig. 3b) and increased LCD (*q* = 0.009, *d* = 0.257; Fig. 3b; Supplementary Dataset 1). HRD+ tumors also showed significantly reduced variability in neoplastic cell roundness (*q* = 0.015, *d* = −0.225; Fig. 3c) and area (*q* = 0.010, *d* = −0.247; Fig. 3c; Supplementary Dataset 1). To further characterize the magnitude of these associations, we evaluated effect sizes using Cohen’s d by comparing feature distributions between HRD+ and HRD− groups. Across evaluated features, effect sizes were small, consistent with the observed overlap in feature distributions (Supplementary Dataset 1). Slide-level Pearson correlations between HRDPath-predicted HRD score and morphological features were assessed using two-sided tests with BH–FDR correction (Supplementary Table 12). In primary-site slides (*n* = 480), significant correlations were observed for elongation (*r* = −0.161, *q* = 0.007), roundness (*r* = 0.216, *q* < 0.001), and LCD (*r* = 0.145, *q* = 0.017). These correlations were generally consistent when all slides were considered (*n* = 720) with significant correlations observed for elongation (*r* = −0.118, *q* = 0.006), roundness (*r* = 0.142, *q* = 0.001), LCD (*r* = 0.183, *q* < 0.001), roundness SD (*r* = −0.146, *q* = 0.001) and nuclear area SD (*r* = −0.131, *q* = 0.003). Together, these results indicate that HRD status is associated with reproducible differences in nuclear morphology.

Given the heterogeneity of HRD-associated genomic alterations, we next examined morphological differences across mutation-defined HRD subgroups. *BRCA1/2*-mutated tumors exhibited significantly increased nuclear roundness (two-sided t-test, BH–FDR *q* = 0.024, Cohen’s *d* = 0.242) and reduced elongation (*q* = 0.007, *d* = −0.260), whereas these features were not significant in *BRCA1*-methylated cancers (*q* ≥ 0.247; Fig. 3d, e). Other HRD+ cancers showed a significantly increased proportion of inflammatory cells, particularly within neoplastic regions (Supplementary Fig. 3e–g). Nuclear area variability was reduced in *BRCA1/2*-mutated (*q* = 0.006) and *BRCA1*-methylated tumors (*q* = 0.017), whereas mean nuclear area did not differ significantly from HRD− tumors (*q* = 0.938 and 0.347, respectively; Fig. 3e, f; Supplementary Dataset 1). *BRCA1/2*-mutated tumors exhibited a significantly higher LCD compared with HRD− tumors (*q* = 0.006, *d* = 0.293; Fig. 3e). Overall, mutation-defined HRD subgroups display distinct but overlapping nuclear and immune microenvironmental features that remain significant after FDR correction.

To gain a better understanding of the contribution of our quantified morphological features to HRDPath predictions, we employed a regression approach to evaluate the predictive contribution of aggregated single-cell morphological features. Specifically, we used linear ridge regression to predict HRDetect scores and logistic regression to classify HRD status, under stratified five-fold cross-validation. Across both all-site and primary-only subsets, ridge regression yielded cross-validated R² values of −0.294. For HRD classification, morphology-only models achieved limited discrimination, with a maximum cross-validated AUC of 0.593 in the primary-only subset (Supplementary Table 13). Together, this confirms that while morphology contributes to the predictivity of HRD, HRD cannot be predicted based on simple aggregated morphological features, and no single morphology variable is sufficient to explain or predict HRD status.

### HRD morphological patterns in metastatic neoplasms

**Fig. 4:**
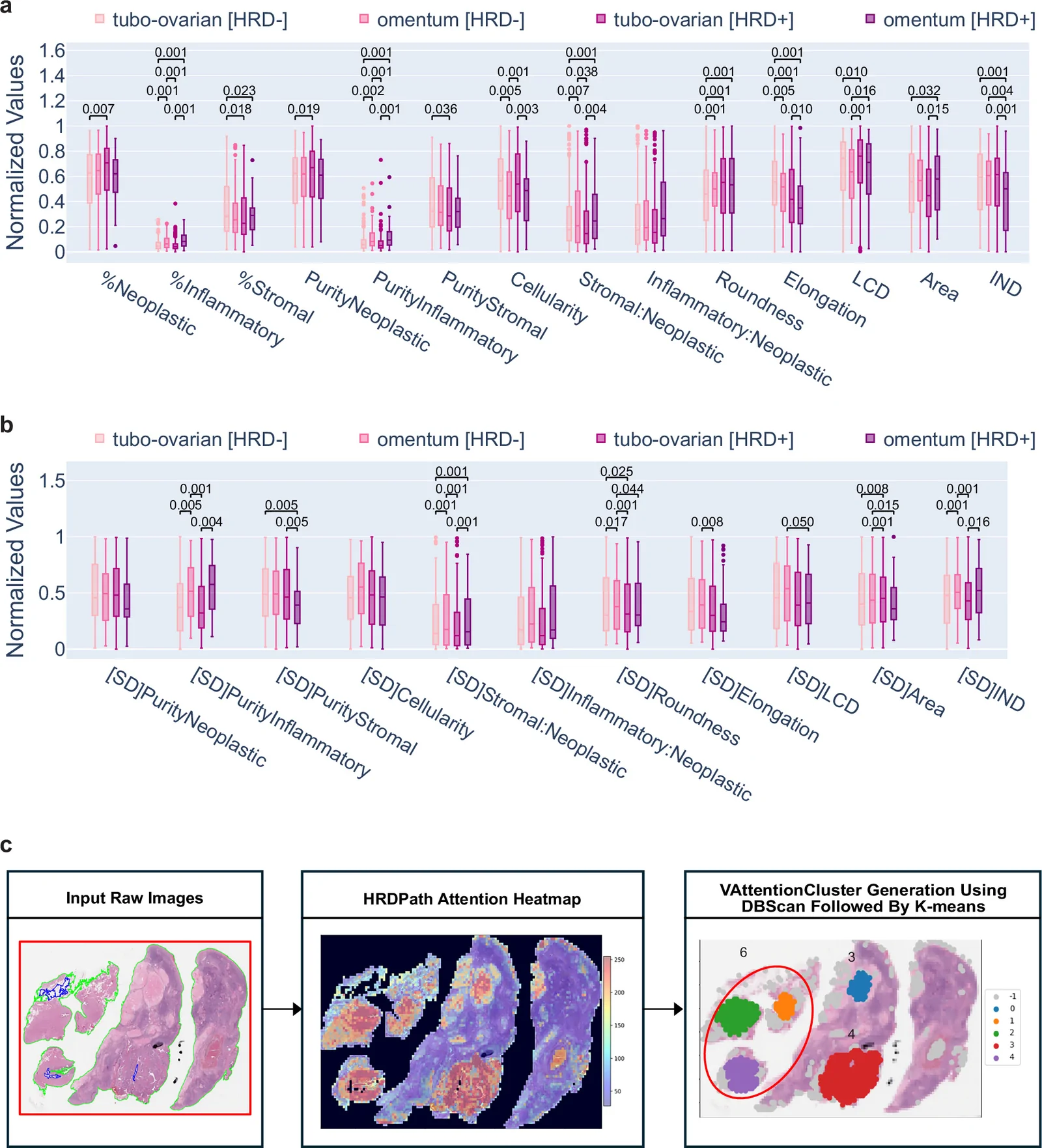
Morphological characterization across primary and metastatic sites and VAttentionClusters. **a** Distribution of TME and morphology features across primary and metastatic sites (tubo-ovarian [HRD−]: 222 WSIs from 62 female patients aged 18–86; omentum [HRD−]: 84 WSIs from 54 female patients aged 18–86; tubo-ovarian [HRD+]: 258 WSIs from 68 female patients aged 37–85; omentum [HRD+]: 67 WSIs from 42 female patients aged 39–82). Center line, median; box bounds, 25th and 75th percentiles; whiskers, 1.5 × IQR; points beyond whiskers, outliers. **b** Standard deviation of features in (**a**) across primary and metastatic sites with box plots and data information defined as in (**a**). **c** VAttentionClusters generation pipeline. (1) HRDPath attention is first clustered according to spatial distribution using DBSCANc and outliers are removed, (2) K-Means clustering (*k* = 8) is applied to attention clusters obtained from (1) considering their attention-weighted RetCCL features to form VAttentionCluster (“Methods”). *P* values in (**a**, **b**) were calculated using two-sided t-tests with FDR correction; only comparisons with corrected *q* < 0.05 are shown (values of *q* < 0.001 are rounded to 0.001). Source data are provided as a Source Data file.

As HRDPath accurately detected HRD+ cancers from images of metastatic HGSOC, we investigated morphological differences by HRD status between primary (tubo-ovarian) and omental sites. The metastatic regions had a higher stromal-to-neoplastic ratio, lower cellularity, higher proportion and purity of inflammatory cells, and lower elongation regardless of HRD status (Fig. 4a). As expected, metastatic regions also showed higher variability (SD) in inflammatory purity, stromal-to-neoplastic cell ratio, and immune-to-neoplastic cell distance (Fig. 4b). Among HRD− tumors, omental metastases had higher nuclear roundness than primary neoplasia (two-sided t-test, BH–FDR *q* = 0.002, Cohen’s *d* = 0.484), whereas the difference in roundness SD did not remain significant after FDR correction (*q* = 0.095, *d* = 0.307; Fig. 4a, b). Among HRD+ tumors, lower SD of stromal purity in omental metastases compared to primary was retained after FDR correction (*q* = 0.006, *d* = −0.392), whereas the difference in neoplastic-cell area did not remain significant (*q* = 0.071, *d* = 0.340; Fig. 4a, b). These results indicate that disease sites are associated with distinct tumor-microenvironment features in HGSOC.

### Analysis of tissue and tumor microenvironment phenotypes

**Fig. 5:**
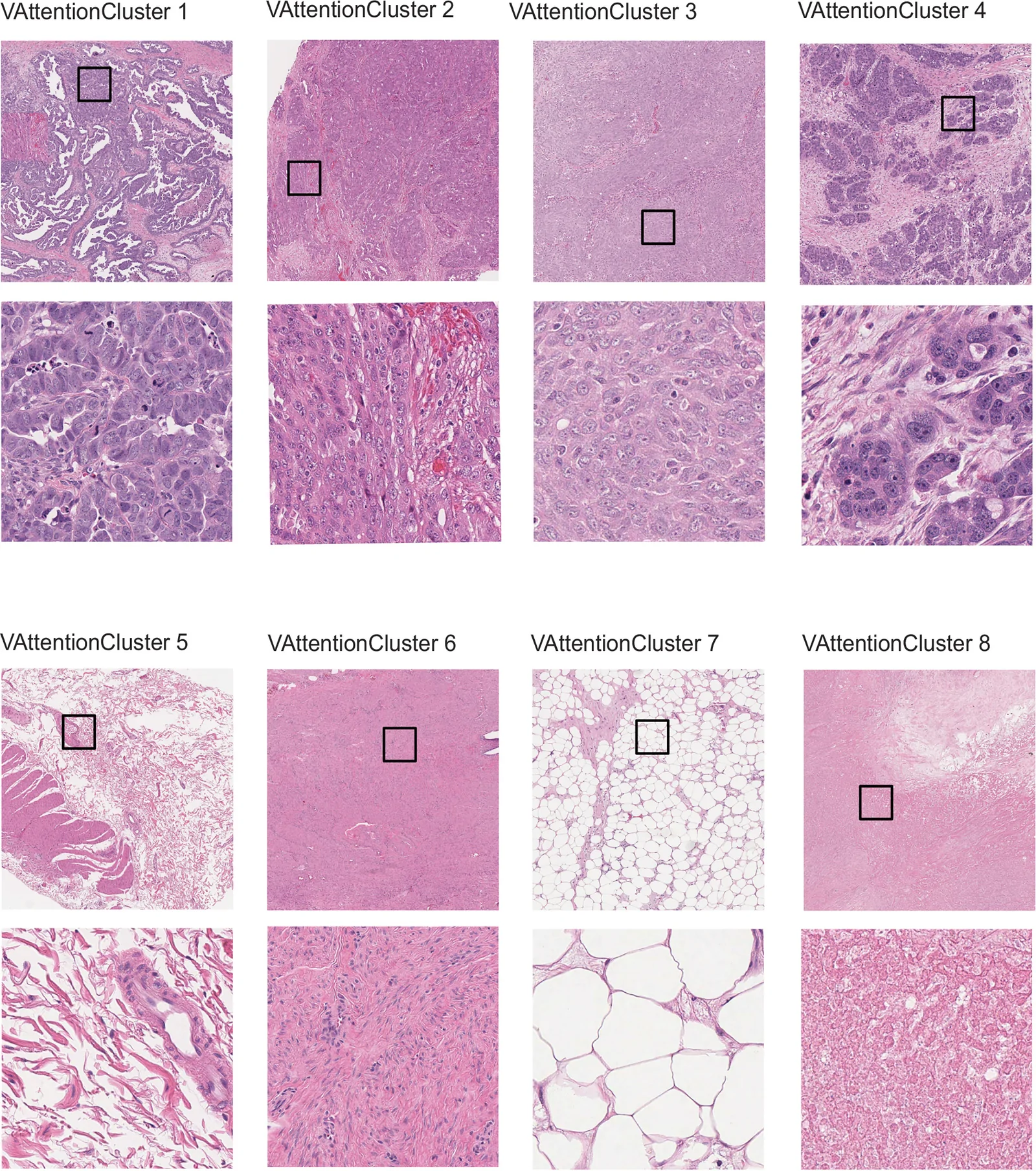
Representative images of different VAttentionClusters. The top row shows a zoomed-out image and the bottom row displays a zoomed-in image of the region outlined by the black box in the top row based on regions selected by HRDPath (*n* = 7 WSIs; 7 female patients aged 42–79). VAttentionClusters 1–4 are dominated by neoplastic cells. VAttentionClusters 5–6 are dominated by stromal and fibrotic tissue. VAttentionCluster 7 comprises adipose tissue, while VAttentionCluster 8 comprises infarcted tissue.

**Fig. 6:**
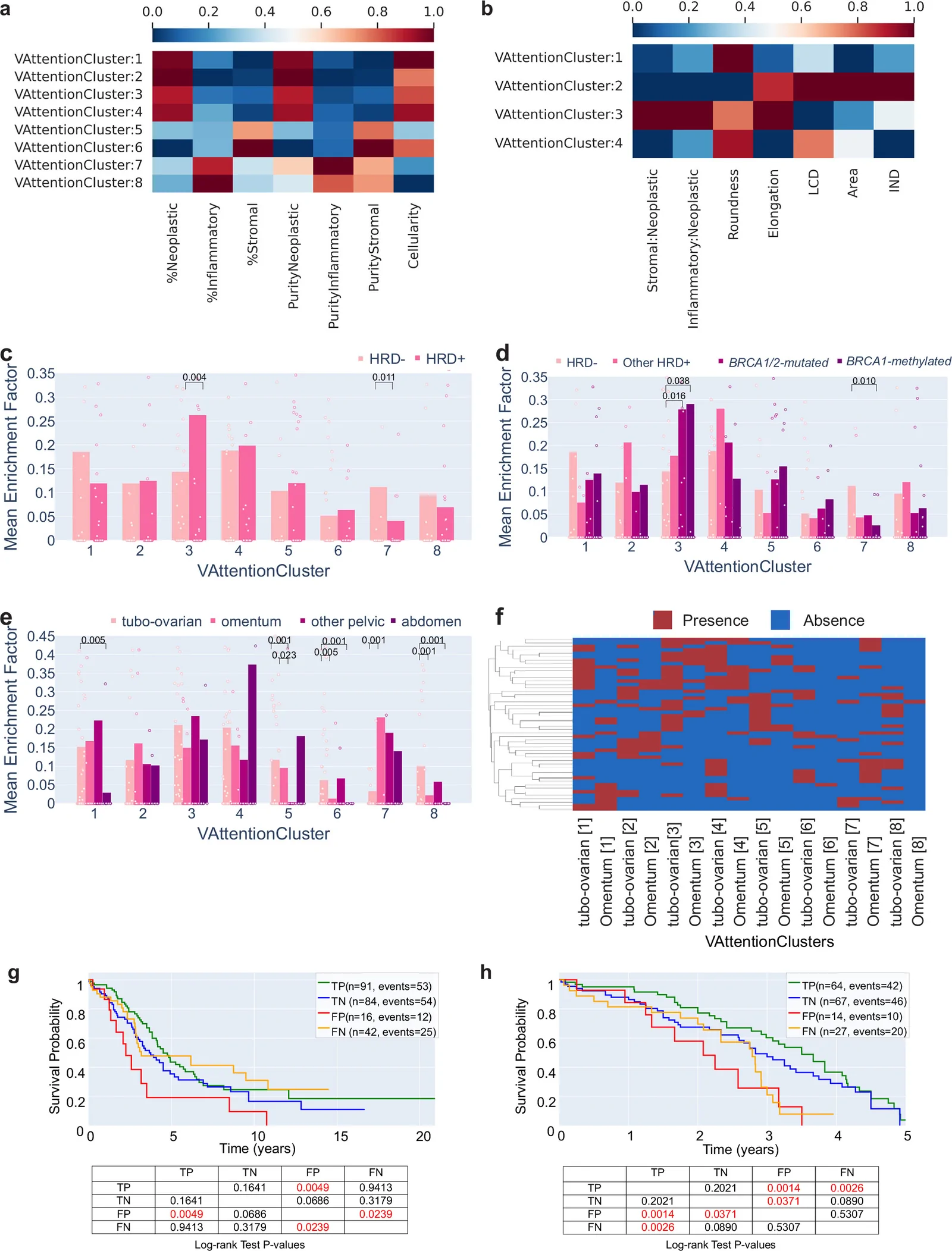
VAttentionClusters enrichment factor distribution across HRD status, genomic alterations, and anatomical locations. **a** Cell type distribution across VAttentionClusters **b** Morphological characterization of VAttentionClusters 1–4 (neoplastic clusters). **c, d** VAttentionCluster enrichment factors across HRD status (**c**) and genomic alterations (**d**), using 486 WSIs from 127 female patients aged 18–86. **e** Anatomical-location comparisons using 478 WSIs from 125 female patients aged 18–86: tubo-ovarian, 368 WSIs; omentum, 82 WSIs; other pelvic organs, 17 WSIs; and abdomen, 11 WSIs. Eight retained WSIs classified as other locations were excluded from panel **e** Of the original 720 WSIs, 234 were excluded from these analyses because model attention did not form suitable spatially coherent clusters under the DBSCAN-based procedure described in “Methods”. **f** Patient-level presence of VAttentionClusters in tubo-ovarian and omental neoplasia, with patients grouped by hierarchical clustering. This paired-site analysis used 228 retained WSIs from 50 female patients with evaluable profiles at both sites aged 39–82. *P* values in **c**–**e** were calculated using two-sided t-tests and adjusted for multiple comparisons using the Benjamini–Hochberg FDR procedure; only comparisons with *q* < 0.05 are shown (*q* *values* < 0.001 are rounded to 0.001). **g, h** Patient-level overall-survival analyses in the integrated UWOV and TCGAOV cohorts: all patients (802 WSIs from 233 female patients aged 18–86) (**g**), and the exploratory subset with observed survival or follow-up time ≤5 years (568 WSIs from 172 female patients aged 37–86) (**h**). Panel **h** excludes patients with an observed time >5 years; it does not apply five-year administrative censoring to the full cohort. Red and blue values in the matrices denote unadjusted *p* < 0.05 and *p* ≥ 0.05, respectively. TP (True Positives), TN (True Negatives), FP (False Positives), FN (False Negatives). **g**, **h** display unadjusted two-sided log-rank *p* values. BH–FDR correction was applied separately to the six pairwise comparisons within each panel to obtain the *q* values reported in the Results. Source data are provided as a Source Data file.

To gain further insights into tissue-level phenotypes associated with HRD and enhance trust in model predictions, we developed an explainability approach to determine key phenotypes based on deep learning features (Fig. 4c). We identified patches that received high attention from HRDPath (“Methods”). Unsupervised DBSCAN clustering was applied to patches, considering the spatial arrangement to form attention regions. These regions were then grouped into eight clusters using k-means clustering based on their RetCCL-derived visual features and attention scores, which we refer to as VAttentionClusters. Inspecting these clusters qualitatively and quantitatively revealed that VAttentionClusters 1, 2, 3, and 4 predominantly consisted of neoplastic cells and exhibited distinct phenotypes (Figs. 5 and 6a, b). VAttentionCluster 1 was significantly enriched in HRD− HGSOC whereas VAttentionCluster 3 was significantly enriched in HRD+ cancers (Fig. 6c). Nuclei in VAttentionClusters 1 and 4 were significantly less elongated and rounder than VAttentionClusters 2 and 3 (Fig. 6b). Moreover, nuclei in VAttentionCluster 3 had the lowest local cell density and were most enriched in tumors harboring *BRCA1/2* alterations (Fig. 6b, d). Interestingly, VAttentionCluster 2, although significantly enriched in Other HRD+ cases, exhibited high nuclear elongation and the lowest roundness among neoplastic clusters (Fig. 6b–d). These results suggest that genetic alterations associated with HRD are correlated with distinct tumor morphologies.

Our VAttentionClusters revealed that HRDPath also identified several microenvironmental signatures associated with HRD status. Specifically, VAttentionClusters 5–8 were characterized by low neoplastic cell proportions and cellularity but were dominated by necrotic, stromal, fibrotic, or adipose tissue regions (Fig. 5). Through qualitative assessment, we found that VAttentionCluster 7 was mainly composed of adipose tissue and was significantly associated with HRD− cancers (Fig. 6c). Conversely, VAttentionClusters 5 and 6 were primarily composed of stromal cells with high fibrosis present in VAttentionCluster 5 supported by its lower cellularity and high stromal proportion (Figs. 5 and 6a). VAttentionCluster 8, on the other hand, was characterized by infarcted tissue and fibrosis with the lowest cellularity and was enriched in both HRD− and Other HRD+ cancers (Fig. 6a–d). These results suggest that HRD+ cancers display location-specific changes in the tumor microenvironment which can provide additional context for HRDPath predictions.

Next, we investigated whether VAttentionClusters tended to localize to specific anatomical regions. All neoplastic clusters were present across different locations, except for VAttentionCluster 1, which showed a low enrichment factor in the abdomen (Fig. 6e). In contrast, abdominal metastases were mostly enriched for VAttentionCluster 4 (Fig. 6e). While stromal VAttentionClusters 5 and 6 were present in primary sites, stromal VAttentionCluster 5 was absent in other pelvic metastases but present in abdominal metastases. Stromal VAttentionCluster 6 was detected in primary sites and other pelvic metastases but absent in abdominal metastases, suggesting location-specific signatures. Adipose VAttentionCluster 7 had a very low enrichment factor in primary sites, while necrotic VAttentionCluster 8 was enriched in tubo-ovarian primary sites (two-sided t-tests, BH–FDR *q* = 0.001, Cohen’s *d* = −0.300 for omentum versus tubo-ovary and *q* < 0.001, *d* = −0.362 for abdomen versus tubo-ovary; Fig. 6e). We also observed that if VAttentionClusters 2–4 were present at the primary site, they were likely to be present at the omental site in that patient (Fig. 6f). These results suggest that phenotypic changes associated with HRD are shared between primary and metastatic locations, but that some tumor-microenvironment phenotypes might be location specific.

### Association of HRDPath predictions and patient outcomes

We found that differences in genomic alterations and morphological heterogeneity could partially explain discordant cases where image-based HRD prediction did not match genomic HRD status (Supplementary Note 5). Cross-cohort comparisons nevertheless identified differences in staining and morphology between TCGAOV and UWOV, together with variation in clinical and molecular features across TCGAOV prediction groups (Supplementary Fig. 4a–e).

Prediction accuracy varied across genomic subgroups, with the highest accuracy observed in *BRCA1*-methylated cancers and the lowest in Other HRD+ cancers lacking *BRCA1/2* alterations (Supplementary Table 14). Notably, incorrectly predicted cases (false-positives and false-negatives) exhibited significantly different morphology in both TCGA and UWOV (Supplementary Figs. 4c–e and 5a–c). These incorrect predictions were not significantly associated with HRD scores, tumor stage, or platinum response. Site-stratified analyses further revealed anatomical location-dependent morphological differences between correctly and incorrectly predicted HRD-positive cases (Supplementary Fig. 6a–d).

We next investigated whether discordant predictions were associated with clinical outcomes. HRDPath predictions were not significantly associated with OS in UWOV (two-sided log-rank *p* = 0.455; Supplementary Fig. 6e). In the integrated UWOV and TCGAOV cohorts (802 WSIs from 233 female patients; Fig. 6g), false-positive cases (HRDPath+/HRD−) had worse survival than true-positive cases (two-sided log-rank test, BH–FDR *q* = 0.030), but the comparison with true-negative cases was not significant (*q* = 0.137). In an exploratory subset restricted to patients with observed survival or follow-up time of five years or less (568 WSIs from 172 female patients; Fig. 6h), false-negative cases (HRDPath−/HRD+) had worse survival than true-positive cases (*q* = 0.008), but not true-negative cases (*q* = 0.134). These exploratory associations suggest that image-based predictions may capture prognostic phenotypes not fully reflected by genomic HRD status.

## Discussion

In this study, we developed HRDPath, a multi-model architecture to predict HRD status using H&E images of HGSOC. HRDPath marks a significant advance with an AUC of 0.846, sensitivity of 0.692, and specificity of 0.938 when considering tubo-ovarian and omental sites. We validated that it performs comparably on unseen images from TCGAOV and PTRC even when fine-tuned using only one image per class in a few-shot learning paradigm. We also show that HRD can be predicted from metastatic tissue slides. HRDPath also achieved state-of-the-art performance in predicting *BRCA1/2* alterations, outperforming existing methods^14,31–33^. Additionally, we performed patient-level classification by incorporating data from multiple slide images and anatomical locations to improve model performance, and we demonstrate that this leads to very high accuracy, sensitivity, and specificity when 6 or more slides are included. Our comprehensive phenotypic profiling reveals important insights on morphological correlates with HRD at cellular and tissue levels which can be exploited in therapy development and target discovery.

Although deep learning models showed great promise in other cancer types^34^, their application to HRD prediction in ovarian cancer has yielded limited performance^32,35,36^. This is likely due to architectural constraints. For instance, DeepHRD utilized a computationally intensive, patch-level classification strategy that not only ignored broader contextual information but also relied on top five patches for its prediction. Furthermore, a shared limitation among these previous methods is the lack of evaluation on truly independent patient cohorts. Another approach required tumor region segmentation followed by extraction of features, which were then fed into a random forest classifier hindering the translation of this approach ^36^. In contrast, our approach only requires patch extraction using CLAM pipeline followed by RetCCL feature extraction. As such, we anticipate that our approach is more robust to inter-dataset variability as it requires minimal preprocessing. Interestingly, using UNI foundation models as feature extractors, instead of RetCCL, did not improve performance based on our data. Importantly, our evaluation of HRDPath on both unseen data within the same cohort and on independent cohorts further underscores the importance of color normalization in addressing domain shifts, particularly in one-shot or few-shot learning scenarios. Overall, our study addresses key gaps in HRD prediction and offers a computationally efficient, end-to-end framework suitable for broader application.

The integration of ViT and DSMIL into HRDPath architecture allowed effective detection of different pathological changes associated with diverse HRD alterations that include copy number amplifications, mutations, and large genomic structural phenotypes. HRDPath not only extracts meaningful features from gigapixel-sized whole-slide tissue images but also identifies the most relevant patches and regions among millions. Specifically, DSMIL selects relevant regions, thereby guiding the transformer model’s attention, while the transformer-based feature fusion method to integrate slide-level and patient-level information. We found that this patient-level and multi-model approach significantly improves the overall performance of HRDPath, resulting in more stable model predictions with three or more slides. Interestingly, the proposed patient-level approach also allowed us to quantify the variability in model predictions, showing that even with one slide, HRDPath tends to be consistent in its predictions. HRDPath outperformed more recent advanced approaches such as the ZoomMIL model, which identifies relevant patches from low-resolution images and analyzes their features at higher resolution. This may be because HRD-associated alterations are more discernible at the single-cell level. Our findings are also consistent with results from our AI in Histopathology Explorer study, where we observed that multi-model architectures often outperform single-model approaches and are less sensitive to dataset size based on comparison of more than 1,400 published studies^37^. These results suggest that diversifying model learning strategies may be more effective than focusing on building a single highly specialized model.

Our explainability framework allows the model to highlight relevant regions and phenotypes, providing clinicians with critical insights to evaluate and validate the trustworthiness of individual predictions. Moreover, many of the differences identified by HRDPath were consistent with previous observations that *BRCA1/2*-mutated ovarian cancers have a higher frequency of certain pathological and invasion patterns^38,39^. Importantly, we also found that *BRCA1*-methylated cancers exhibited distinct morphologies. While such morphological differences have been previously observed by pathologists, deep learning models enable their systematic and quantitative characterization and robust discrimination between HRD+ and HRD− cancers. Specifically, HRDPath allowed systematic and quantitative discovery of the multitude of phenotypes associated with HRD+ cancers using WSI images, integrating information from millions  of patches in a non-linear manner, which might be challenging to detect by the naked eye alone or based on simple aggregation of a few morphological features. Our results are generally consistent with the features reported in HRD+ breast cancers such as increased fibrosis, and polymorphic nuclei^34^. The decrease in the overall proportion of stromal cells in HRD+ HGSOC might reflect a more complex interplay of features, as the reduced stromal cell count could be indicative of increased fibrosis as it was also associated with reduced cellularity. This interpretation is supported by our tissue-level analysis, which showed a higher enrichment of fibrosis-associated VAttentionClusters 5 and 6 in cancers with *BRCA1/2* genomic alterations. Unlike previous reports in HRD+ breast cancers, our analysis does not support increased necrosis as a distinguishing feature of HRD+ HGSOC, as reported in HRD+ breast cancers. Our explainability pipeline can be potentially used to discover phenotypic differences but also to increase model trustworthiness, by highlighting regions aligned with model predictions.

Analysis of morphological features contribution indicates that individual nuclear morphology features capture biologically-relevant aspects of HRD-associated tissue alterations. In particular, nuclei in HRD+ primary and metastatic cancers tended to exhibit reduced elongation compared with HRD− cases. Such alterations in nuclear morphology may reflect elevated proliferative activity associated with impaired DNA repair, as dividing cells often adopt a rounder morphology during mitosis^40^. Moreover, the high proliferation rate, as reflected by the higher neoplastic density, can disrupt epithelial polarity, as well as cell adhesion and cytoskeleton^41^. Together, these findings indicate that individual morphology features capture biologically plausible aspects of HRD-related tissue alteration, while HRDPath integrates these signals within a broader spatial and contextual framework across whole-slide images.

Interestingly, we identified a subset of HRD+ cancers that showed significantly increased elongation and reduced roundness, especially in cancers with no *BRCA1/2*-associated alterations. This might be associated with epithelial-to-mesenchymal transition, which has been linked to differential DNA damage repair response^42,43^. However, further experimental validation is required to confirm the biological processes driving these differences. Intriguingly, misclassified cases, and especially HRDPath+/HRD−, were associated with significantly worse patient outcomes, suggesting that image-derived morphological patterns may capture clinically relevant phenotypes not fully reflected by genomic HRD assays alone. Our current cohort involves heterogeneous treatments, where many patients were treated before the frontline FDA approvals of PARP inhibitors for ovarian cancer in 2018^44^. An interesting direction for future research is to determine whether these morphological signatures also correlate specifically with response to PARP inhibitor and to evaluate how morphological patterns can be leveraged to refine prognostic stratification in HGSOC.

The evaluation of our balanced in-house UWOV and TCGA, along with additional evaluation on metastatic slides, provides strong evidence of the generalizability of our approach. While HRDetect provides a well-validated genomic signature with demonstrated correlation to *BRCA1/2* deficiency^11,45,46^, we recognize that it is not FDA-approved. Nonetheless, the validation on two independent cohorts (TCGA and PTRC) where myChoice labels were available addresses this limitation and demonstrates consistent performance based on few-shot learning. Broader validation across more diverse patient populations and balanced datasets will be necessary to advance HRDPath translation potential, especially given that PTRC dataset included only five HRD− patients. Additional validation of HRDPath using multiple clinical HRD assays will be important to support clinical translation.

HRDPath operates at the patient level by aggregating information across multiple whole-slide images, aligning with standard pathological workflows involving multiple specimens per patient. Sensitivity increases substantially with the number of slides available, demonstrating that multi-slide aggregation improves confidence in HRD classification. To mitigate the limited sensitivity of HRDPath and support clinical translation, we propose to optimize by establishing minimum performance thresholds based on available diagnostic input. For instance, our model achieves a sensitivity of 0.910 and a specificity of 1000 when 6 slides are used. When using two or more WSI from the primary site, we obtain an average sensitivity of 0.930 and an average specificity of 0.890. Moreover, our consistency measure can provide clinicians with an estimate of model confidence, providing actionable insights. We anticipate that sensitivity and overall performance may be further improved through training on larger and more diverse datasets, incorporating alternative learning strategies such as self-supervised or weakly supervised pretraining, and integrating richer clinical or molecular annotations.

We believe that HRDPath has the potential to be translated into a digital biomarker for detecting HRD in ovarian cancer patients, integrating histopathology images with a transformer-based deep learning model for rapid assessment of genetic signatures. For example, to aid risk stratification, prioritize cases for molecular testing, or provide cost-effective decision support in settings where genomic assays are delayed or resource intensive. Our approach is to demonstrate significant improvement over existing methods and to predict HRD from metastatic tumors, thus expanding its potential clinical relevance beyond primary sites. Additionally, our morphological analysis uncovered previously unrecognized features of HRD+ HGSOC, offering insights into cancer biology and tumor–microenvironment interactions. Together, these findings position HRDPath as a promising step toward image-based digital biomarkers for HRD.

## Methods

### Ethics statement

Ethical approval for data collection and research use in the original study^12^ was obtained from the University of Washington Human Subjects Division Institutional Review Board under approvals IRB 2872 and IRB 2802. All participants provided written informed consent before enrollment. The study design was approved by the King’s College London Research Ethics Committee (LRM-24/25-36190). All data analyzed in this study were anonymized, and no identifiable personal information was available to the researchers.

### Statistics and reproducibility

This study was designed as a retrospective computational pathology study to develop and evaluate HRDPath, a patient-level deep learning model for predicting HRD status from H&E-stained whole-slide images of HGSOC. Model development was performed using the UWOV cohort, and model performance was evaluated on held-out UWOV cases and publicly available independent datasets, including TCGAOV and PTRC-HGSOC. HRDPath was benchmarked against established and recent weakly supervised histopathology models, including CLAM-SB, CLAM-MB, DSMIL, ViT, DeepHRD, ZoomMIL, and UNI, using harmonized patient-level evaluation procedures where applicable. Additional robustness analyses assessed performance across anatomical tumor sites, few-shot adaptation to independent datasets, *BRCA1/2* alteration prediction through transfer learning, intra-patient prediction consistency across multiple whole-slide images, and attention-based morphological interpretability.

This study is reported in accordance with the TRIPOD + AI reporting guideline for studies developing and evaluating clinical prediction models that use regression or machine-learning methods. A completed TRIPOD + AI checklist is provided with the submission.

Sample size was determined by the availability of eligible cases with digitized whole-slide images and corresponding molecular HRD annotations. No statistical method was used to predetermine sample size. Cases were included when the required histopathological images and HRD labels were available. Data were excluded only according to predefined eligibility, annotation availability, and preprocessing criteria, including lack of matched HRD annotation and digitized H&E images, feature-level outlier filtering for morphology analysis, and exclusion of slides labeled as “other location” from anatomical site-specific morphology analyses. These exclusions and the corresponding analysis denominators are reported in the relevant Methods subsections and supplementary tables. No imputation was performed; cases lacking matched HRD annotations or digitized H&E images were excluded.

For model training and evaluation, all slides from the same patient were assigned to the same data split to avoid patient-level data leakage. The experiments were not randomized. The researchers were not blinded to allocation during experiments and outcome assessment. For morphology validation, HoVer-NeXt-derived cell-type proportions were compared with reference scores generated by manual qualitative assessment of 40 randomly selected whole-slide images by a pathologist.

Model performance was evaluated using the area under the receiver operating characteristic curve, sensitivity, and specificity. Continuous HRDPath-predicted scores were compared with genomic HRD scores using correlation analysis where applicable. Intra-patient consistency was assessed by evaluating all possible combinations of available slides per patient and aggregating prediction accuracy across patients with at least the corresponding number of slides. Morphological differences between HRD-positive and HRD-negative tumors were assessed using two-sided two-sample t-tests. Given the number of morphological features evaluated, *p* values were adjusted using the Benjamini–Hochberg false discovery rate procedure, and statistical significance was assessed using FDR-adjusted *p* values unless otherwise stated. Effect sizes were quantified using Cohen’s d. Associations involving attention-derived phenotypes, anatomical sites, and HoVer-NeXt-derived features were analyzed as described in the relevant Methods subsections.

Reproducibility was supported by patient-level data partitioning, fixed random seeds for model initialization and training, standardized whole-slide image preprocessing, harmonized benchmarking across comparator models, validation on independent public datasets, few-shot adaptation experiments, anatomical site-specific evaluation, intra-patient slide-combination analysis, and pathologist comparison for cell-type proportion estimates. Statistical analyses were performed using Python v3.10 with SciPy and scikit-learn libraries.

### Patient cohort

#### University of Washington Ovarian (UWOV) dataset and patient cohort

Patients diagnosed with high-grade ovarian cancer, undergoing surgery, and receiving secondary care at a single center, the University of Washington, in Seattle, Washington, USA were enrolled in a biorepository initiated in 2000 and approved by the Institutional Review Board University of Washington Human Subjects Division IRB 2872 and 2802. For the present study, data were obtained from the biorepository in 2024; therefore, only eligible patients enrolled and with data available at the time of data extraction were considered for inclusion in this study. Sex was considered in the study design through the inclusion of patients recorded as female in the clinical record. All patients provided written informed consent prior to enrollment. In this study, 212 representative patients with 222 samples were selected. Of these, 152 patients had both HRD scores generated, and H&E images digitized (Supplementary Table 1). All patients were followed until death and, if alive, censored at the last follow-up. Detailed treatment data were not available, and treatment was not included as a predictor or otherwise handled during model development or evaluation.

In this dataset, HRD status is defined using HRDetect algorithm, with a cutoff of 0.95 for HRD-positive (HRD+) classification. HRDetect is a whole-genome sequencing-based algorithm that predicts cancers with high, intermediate or low HRD score^47,48^. It is a weighted model that combines the contributions of six distinguishing mutational signatures to produce a singel score predictive of a defect in the homologous recombination DNA repair pathway. Following signature assignment, the contributions of substitution signatures SBS3 and SBS8, rearrangement signatures RefSig R3 and R5, loss of heterozygosity index and proportion of small deletions in regions of microhomology were used to calculate the HRDetect probability score. This score is predictive of the response to PARP inhibitors in HGSOC^12^.

For supervised learning, we adopted a binary HRD definition, grouping HRDetect-intermediate cases with HRDetect-low cases as HRD-negative in line with previous work^12^.

#### TCGA Ovarian cancer whole-slide image dataset

This study was validated on publicly available H&E diagnostic slides and whole genomic sequence from the TCGA Ovarian dataset (https://portal.gdc.cancer.gov/). The 82 diagnostic WSIs from 81 patients were processed using the same pipeline as UWOV. The patient-level training/test split comprised 60/21 patients, corresponding to 61/21 WSIs; both WSIs from the patient with two slides were assigned to the training set (Supplementary Table 1). The TCGAOV HRD scores used in this study were not generated or collected by the authors; they were obtained from the publicly available dataset reported by Kang et al.^49^. The underlying public HRD-score data are available at https://api.gdc.cancer.gov/data/66dd07d7-6366-4774-83c3-5ad1e22b177e.

#### PTRC-HGSOC whole slide image dataset

198 H&E slides from PTRC-HGSOC were processed using the same pipeline. The HRD score was defined as the sum of LOH, LST, and TAI provided in the original paper^50^. PTRC-HGSOC is a publicly available dataset hosted by The Cancer Imaging Archive (See Data availablity).

### Image Preprocessing

To make WSI images suitable for deep learning models, we adapted the processing steps of CLAM. Each WSI is cut into a sequence of square patches of 1024 pixels at 40× resolution (0.25 µm/pixel) at the selected level dimensions. The level dimension that has a resolution closest to 1e^8^ was selected. Background patches were filtered using median thresholding. To mitigate the effect of H&E staining variation within the dataset, the patches were normalized based on slide-level statistics. For each whole-slide image, the mean and standard deviation of the red, green, and blue channels were computed across all pixels within the slide. Subsequently, each pixel value in the corresponding patch was standardized by subtracting the channel-specific mean and dividing by the channel-specific standard deviation. Each patch was resized via bi-linear interpolation to 384 pixels, which is equivalent to ~15× magnification and is sufficient to retain nuclear and cytological features needed for cell-level analysis. Then, patches were processed using RetCCL, a self-supervised model trained on 32,000 pan-cancer whole slide images^21^, to obtain 2048 features per slide.

### Patient-Level Multi-task Learning

We included HRD score prediction as a complementary task because the HRD+ group is heterogeneous. Some HRD+ patients exhibit borderline HRD scores that may reflect distinct morphological characteristics. By jointly optimizing for both classification and regression, the model is encouraged to capture finer-grained morphological cues, enhancing generalizability and interpretability. Specifically, our framework simultaneously performs (i) HRD status classification, a binary prediction task (HRD+ vs. HRD−), and (ii) HRD score regression, a continuous prediction of the homologous recombination deficiency (HRD) score derived from genomic data, with both tasks given equal weights using the following objective function:

1 $${{{\boldsymbol{{{\mathscr{L}}}}}}}_{{{{\boldsymbol{total}}}}}=	 {{{\boldsymbol{{{\mathscr{L}}}}}}}_{{{{\boldsymbol{CE}}}}}+{{{\boldsymbol{{{\mathscr{L}}}}}}}_{{{{\boldsymbol{MSE}}}}}=-\frac{{\sum }_{{{{\boldsymbol{i}}}}={{{\bf{1}}}}}^{{{{\boldsymbol{N}}}}}{\sum }_{{{{\boldsymbol{c}}}}={{{\bf{0}}}}}^{{{{\bf{1}}}}}{{{{\boldsymbol{y}}}}}_{{{{\boldsymbol{ic}}}}}^{{{{\boldsymbol{status}}}}}{{{\mathbf{log }}}}{\left({{{{\bf{y}}}}}_{{{{\boldsymbol{ic}}}}}^{{{{\boldsymbol{statu}}}}{{{{\boldsymbol{s}}}}}^{{\prime} }}\right)}}{{{{\boldsymbol{N}}}}} \\ 	+\frac{{\sum }_{{{{\boldsymbol{i}}}}={{{\bf{1}}}}}^{{{{\boldsymbol{N}}}}}{{({{{{\bf{y}}}}}_{{{{\boldsymbol{i}}}}}^{{{{\boldsymbol{score}}}}}-{{{{\bf{y}}}}}_{{{{\boldsymbol{i}}}}}^{{{{\boldsymbol{score}}}}{\prime} })}}^{{{{\bf{2}}}}}}{{{{\boldsymbol{N}}}}}$$

Where ${{{{\mathscr{L}}}}}_{{total}}$ represents the combined multi-task loss, consisting of classification cross entropy loss ${{{{\mathscr{L}}}}}_{{CE}}$ and regression mean-squared error loss ${{{{\mathscr{L}}}}}_{{MSE}}$. Here, N, ${y}_{i}^{{statu}{s}^{{\prime} }}$, ${y}_{i}^{{status}}$, ${y}_{i}^{{score}{\prime} }\,$, ${y}_{i}^{{score}}$ denotes the total number of training samples; predicted HRD status; ground truth HRD status; predicted HRD score, ground truth HRD score, respectively. Equal weighting was adopted to avoid introducing additional hyperparameters and to encourage balanced learning across the two closely related tasks; no explicit optimization of loss weight was performed.

### HRDPath architecture

HRDPath is a three-stream architecture integrating a DSMIL module, a global Vision Transformer (ViT), and a Local Window Transformer to integrate fine-grained local context with slide-level global semantics. (Box 1; Supplementary Fig. 1a). The model takes a set of patch-level features ${{{\bf{F}}}}\,\epsilon {{\mathbb{R}}}^{N\times d}$ as input, where N denotes the number of cleaned image patches from a slide and d is the feature dimension. In the first stream, DSMIL identifies coarse-grained regions of interest (ROIs) and produces a slide-level feature embedding ${{{{\bf{Z}}}}}_{{DSMIL}}\epsilon {{\mathbb{R}}}^{d}$ through attention-based pooling. The ROIs are also used to guide local feature selection for the third stream. In the second stream, the full set of patch-level features is prepended with a CLS token ${{{{\bf{C}}}}}_{G}\epsilon {{\mathbb{R}}}^{d}$ and passed through ${{ViT}}_{G}$. The final CLS token embedding ${{{{\bf{Z}}}}}_{{ViT}}\epsilon {{\mathbb{R}}}^{d}$ is extracted as a global semantic representation. The third-stream constructs k fixed-size windows (of size w×w) centered on the ROIs identified by DSMIL (Supplementary Fig. 1d–f) where hyperparameter *k* is identified by experimentation. DSMIL’s lightweight architecture allowed faster training and quick identification of critical patch phenotypes which can serve as smooth additional supervision to explore by transformer encoders. Size of each window remains fixed to enable parallelism for efficiency. Each local window, after appending a window-specific CLS token ${{{{\bf{C}}}}}_{L}^{k}\epsilon {{\mathbb{R}}}^{d}$ and applying absolute positional encoding, has shape ${{\mathbb{R}}}^{(1+{w}^{2})\times d}$ and is processed independently through ${{ViT}}_{L}$. The k resulting CLS embeddings are averaged to yield the fine-grained local representation ${{{{\bf{Z}}}}}_{{local}}\epsilon {{\mathbb{R}}}^{d}$. The outputs from the three streams, ${{{{\bf{Z}}}}}_{{DSMIL}}$, ${{{{\bf{Z}}}}}_{{ViT}}$, and ${{{{\bf{Z}}}}}_{{local}}$ are concatenated to form a slide-level representation ${{{{\bf{Z}}}}}_{{slide}}\epsilon {{\mathbb{R}}}^{3\times d}$. For a patient with S slides, these are concatenated across slides to yield a patient-specific tensor ${{{{\bf{Z}}}}}_{{patient}}\epsilon {{\mathbb{R}}}^{S\times 3\times d}$. A CLS token ${{{{\bf{C}}}}}_{P}\epsilon {{\mathbb{R}}}^{d}$ is then prepended, resulting in an input of shape ${{\mathbb{R}}}^{(1+S\times 3)\times d}$, which is passed through a patient-level transformer to model inter-slide dependencies and enhance cross-slide pathological pattern recognition. The output CLS token from this transformer (also shape ${{\mathbb{R}}}^{d}$) serves as the final patient-level embedding. This embedding is then passed to a multi-task prediction head for joint learning of two correlated downstream tasks: HRD status classification (binary) and HRD score regression (continuous). All model components are initialized using Kaiming uniform initialization with fixed random seeds and trained end-to-end using a unified optimization framework. Eight attention heads were utilized, which was the number utilized in the original ViT paper^51^. The number of encoder layers of 2 was determined based on available computational resources, and these are now included in our methods. Final HRDPath hyperparameters used for reported performance (transformer encoder depth: 2, attention heads: 8, dimension: 1024, *k* = 8, *w* = 32, dropout = 0.1 (Uniform in all transformer encoder layers and multi-layer perceptron), learning rate: 1e-4, weight decay: 1e-5, AdamW optimizer, batch size:1, number of training epochs: 150).

### Model benchmarking implementation details

To ensure consistent and fair benchmarking, all models were evaluated using a standardized patient-level aggregation module. The input resolutions and adaptation strategies varied across models. All models utilized features extracted at ×15 magnification. The UNI model was evaluated exclusively at 15×, using a pretrained UNI2-h backbone as a frozen feature extractor to generate patch-level embeddings using attention pooling. All models output a slide-level representation from their original architecture, which was aggregated by HRDPath’s patient-level transformer encoder for patient-level prediction. DeepHRD was implemented in a two-stage, non-end-to-end pipeline. Initially, DeepHRD was trained on 7.5× resolution patches to identify regions of interest (ROIs). The resulting model was then used to guide ROI selection for training a second DeepHRD model at 15× resolution. DeepHRD also employs an ensemble of ResNet-18 backbones, and for each ensemble member, the top-scoring patches were averaged to obtain slide-level features (following the original study). The final slide representation was derived by averaging across ensemble outputs. ZoomMIL also incorporates a multi-resolution approach: it first encodes WSIs at ×7.5 magnification to identify ROIs, then re-encodes those regions at 15× resolution. The final slide-level representation consists of the concatenation of the 7.5× and 15× features (ZoomMIL originally used three scales, including a thumbnail scale; for a fair comparison, we fixed it to two scales). This unified aggregation strategy ensures that model comparisons focus on differences in feature extraction and representation learning, rather than downstream fusion or classification methods.

### Intra-patient consistency analysis

Each patient in our dataset is associated with a variable number of slides. To assess the consistency and robustness of model predictions at the patient level, we designed a systematic experiment analyzing how prediction accuracy varies with the number of slides available per patient. For each patient with *n* slides (where *n* ≥ 1), we evaluated model performance across all possible combinations of *i* slides (1 ≤ *i* ≤ *n*). For instance, for a patient with three slides labeled (a, b, c), when evaluating performance at *i* = 2, we considered all combinations of slide pairs: (a, b), (a, c), and (b, c). The model was independently run on each combination, and the resulting patient-level predictions were aggregated and compared to the ground truth to calculate average prediction accuracy.

We repeated this process across all patients for increasing values of *i*, ranging from 1 to the maximum number of slides observed in our dataset. The aggregated accuracy was computed by averaging performance across all patients with at least *i* slides.

To further investigate the impact of predictive confidence, we computed agreement scores for each prediction combination, defined as the proportion of constituent slide-level predictions. This approach allowed us to quantify not only the marginal benefit of incorporating more slides but also the extent to which consistency among slides correlates with improved prediction reliability. Importantly, inference is computationally efficient as each slide requires only ~2.4 s to process.

### Morphology analysis

We performed single-cell morphology analysis using Hover-NeXt pretrained on the PanNuke^52^ dataset. We qualitatively assessed several model variants and found that pannuke_convnextv2_tiny_3 version performs best. Cells were classified as neoplastic, inflammatory, or connective (stromal). Since Hover-NeXt was not originally trained on images of ovarian cancers, we validated its performance by comparing the model’s output to reference scores generated through manual qualitative assessment by a pathologist for 40 randomly selected WSIs. We observed good correlations for HoVer-NeXt-based scores and pathologist scores of neoplastic cell proportion (Two-sided Spearman correlation *ρ* = 0.67, *q* < 0.001) and stromal cell proportion (Two-sided Spearman correlation *ρ* = 0.76, *q* = 0.004).

### Morphology feature computation

Morphological and pathological patterns were captured by evaluating features including cell proportion and purity, neoplastic cell roundness, elongation, area, local cell density, and distance to the nearest immune cells (IND). Cell proportions were obtained by calculating the percentage of a certain cell type contained in each slide, while the purity of a certain cell type was the slide average of the patch cell proportion. Cellularity is the total number of identified cells normalized to the region area (patch or high-attention region). Roundness, elongation, and area were computed from segmented cell contours using the Python OpenCV library. Local cell density (LCD) was defined as the inverse of the mean Euclidean distance between cell centroids within a region. Larger mean intercellular distances therefore yield lower LCD values, whereas smaller mean distances yield higher LCD values. The distance between neoplastic cells and immune cells (IND) was obtained by calculating the mean distance of the nearest 10 immune cells to each neoplastic cell. Z-score normalization was applied to the features, using the mean and standard deviation of the entire dataset. Outliers with an absolute z-score greater than 3 were filtered out. All features were then scaled to a range between 0 and 1. The morphology of neoplasms with different genomic alterations was computed across all anatomical locations (*n* = 720 slides; HRD−: 349 slides; Other HRD+: 75 slides; *BRCA1/2*-mutated:197 slides; *BRCA1-methylated: 99 slides*). To assess morphological features across different anatomical locations, 702 slides were analyzed, excluding those labeled “other location”.

### Statistical analysis of morphological features

To compare morphological differences between HRD+ and HRD− tumors, we applied two-sided two-sample *t*-tests. Given the large number of morphological features evaluated, *p* values were corrected for multiple hypothesis testing using the Benjamini–Hochberg false discovery rate (FDR) procedure. Statistical significance was assessed based on FDR-adjusted *p* values unless otherwise stated.

In addition to statistical significance, effect sizes were explicitly quantified to account for the large sample sizes inherent to cell-level analyses. Cohen’s *d* was reported for *t*-tests. These effect size measures were used to assess the magnitude of morphological differences independently of sample size. All statistical results, including raw *p* values, FDR-adjusted *p* values, and effect sizes, are provided in Supplementary Dataset 1.

### VAttentionClusters generation

HRDPath is a patient-level transformer-based deep learning model with two levels of attention: (i) patient-level attention to assign a score to each WSI to identify importance, and (ii) slide-level attention to demonstrate HRD-related regions within WSI. VAttentionClusters were defined based on HRDPath attention using a multi-stage approach (Fig. 4c). DBSCAN clustering (epsilon = 3.5) was applied based on the HRDPath attention score and spatial distribution to identify attention clusters within each WSI and filter patches with inconsistent spatial attention patterns. This resulted in the identification of 1542 attention clusters. To characterize semantically meaningful morphological patterns within high-attention regions, we implemented k-means clustering on identified attention clusters regarding their visual features, and we qualitatively evaluated whether the resulting clusters captured distinct phenotypes and tissue regions. This design enables a conceptual correspondence between clusters and attention heads, supporting interpretability under the hypothesis that each head captures a distinct morphological signature^53^.

### VAttentionCluster enrichment factor

The enrichment factor is the product of VAttentionCluster proportion and the percentage of enrichment. The VAttentionCluster proportion is the ratio of the number of VAttentionClusters to all identified attention clusters in a slide. The percentage of enrichment is computed by dividing the regions of a specific VAttentionCluster occupied in a slide by the entire slide region.

Associations between HRDPath attention and cellular morphological features extracted by HoVer-NeXt were evaluated using two-sided t-tests. *p* values were adjusted for multiple comparisons using the Benjamini–Hochberg FDR procedure, with significance defined as *q* < 0.05 unless otherwise stated. Cellular morphological features were normalized and standardized, and outliers (>3 standard deviations) were excluded.

### Reporting summary

Further information on research design is available in the Nature Portfolio Reporting Summary linked to this article.

## Supplementary information

Supplementary Information.

Description of Additional Supplementary Files

Supplementary Dataset 1

Reporting Summary

Transparent Peer Review File

## Source data

Source Data

## Data Availability

TCGAOV and PTRC-HGSOC datasets are publicly available from their respective repositories. The UWOV is available under restricted access due to patient privacy and consent restrictions. Access may be granted for ethically approved, bona fide research projects that are consistent with the participants’ consent and subject to approval by the study team and completion of any required data access agreements. Requests should be directed to the corresponding author, who will aim to respond within 4 weeks. If access is approved, the data will be made available for the duration specified in the approved project/data access agreement. No new data were generated for this study. Source data are provided with this paper as Source Data file. The HRD scores were provided by Banda et al. for use in the present study and included in the Source Data file^12^. The underlying genomic data from which these scores were derived were not accessed nor used in this study and can be requested by contacting the authors of the original study^12^. The TCGAOV whole-slide images and genomic data are available through the Genomic Data Commons (https://portal.gdc.cancer.gov/), and PTRC-HGSOC is available through TCIA (https://www.cancerimagingarchive.net/collection/ptrc-hgsoc/). The TCGAOV HRD scores were not generated or collected by the authors; they were obtained from Kang et al. (2022; https://doi.org/10.1177/03000605221133655) and are publicly available from the Genomic Data Commons (https://api.gdc.cancer.gov/data/66dd07d7-6366-4774-83c3-5ad1e22b177e). Source data are provided with this paper.

## Code availability

The code used in this study is not publicly available because it is subject to institutional intellectual property and commercialization processes related to the clinical translation of HRDPath. Access may be considered upon reasonable request to the corresponding author, subject to institutional intellectual property policies and any required agreements.
