# Supplementary material for: HRDPath: an explainable multi-model deep learning architecture for predicting homologous recombination deficiency from histopathology images
Source: Nat Commun. 2026 Oct 21;17:10870. doi: 10.1038/s41467-026-78430-y (PMC13686980; doi:10.1038/s41467-026-78430-y)
Supplement: Supplementary file 1 [file 41467_2026_78430_MOESM1_ESM.pdf]

Supplementary Figures

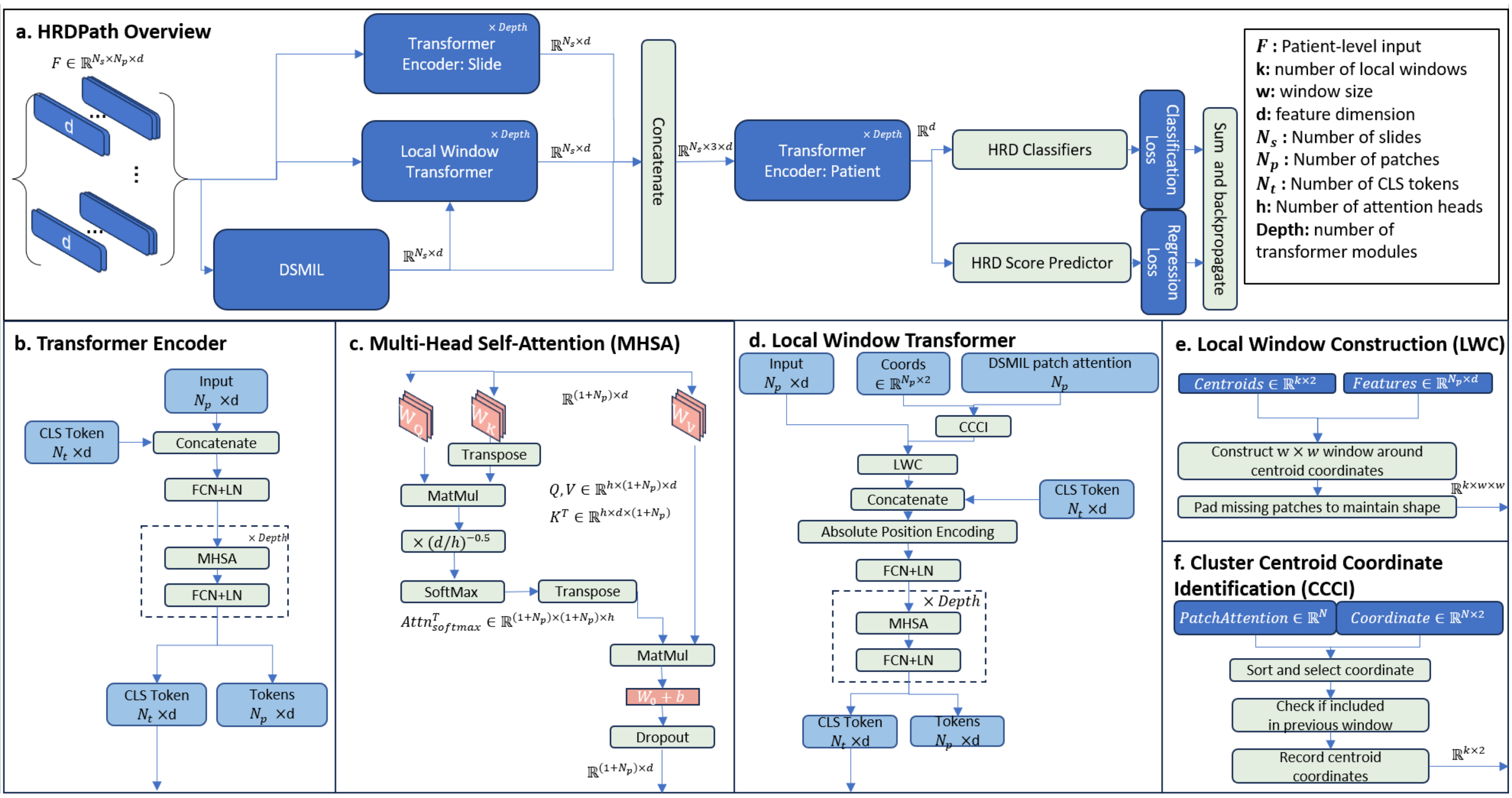

Supplementary Figure 1. Overview of HRDPath architecture. a. HRDPath overview, first processed by DSMIL, then its attention is used for dual-branch procedure. Finally, slide-level representations of three streams are concatenated and processed by patient-level transformer encoder for patient-level representation and multi-task predictions. b. Transformer encoder overview (FCN: fully connected layer; LN: layer normalisation). c. MHSA overview (MatMul: matrix multiplication). d. Local window transformer overview. e. Local window construction (LWC) overview. f. Cluster Centroid Coordinate Identification (CCCI) overview.

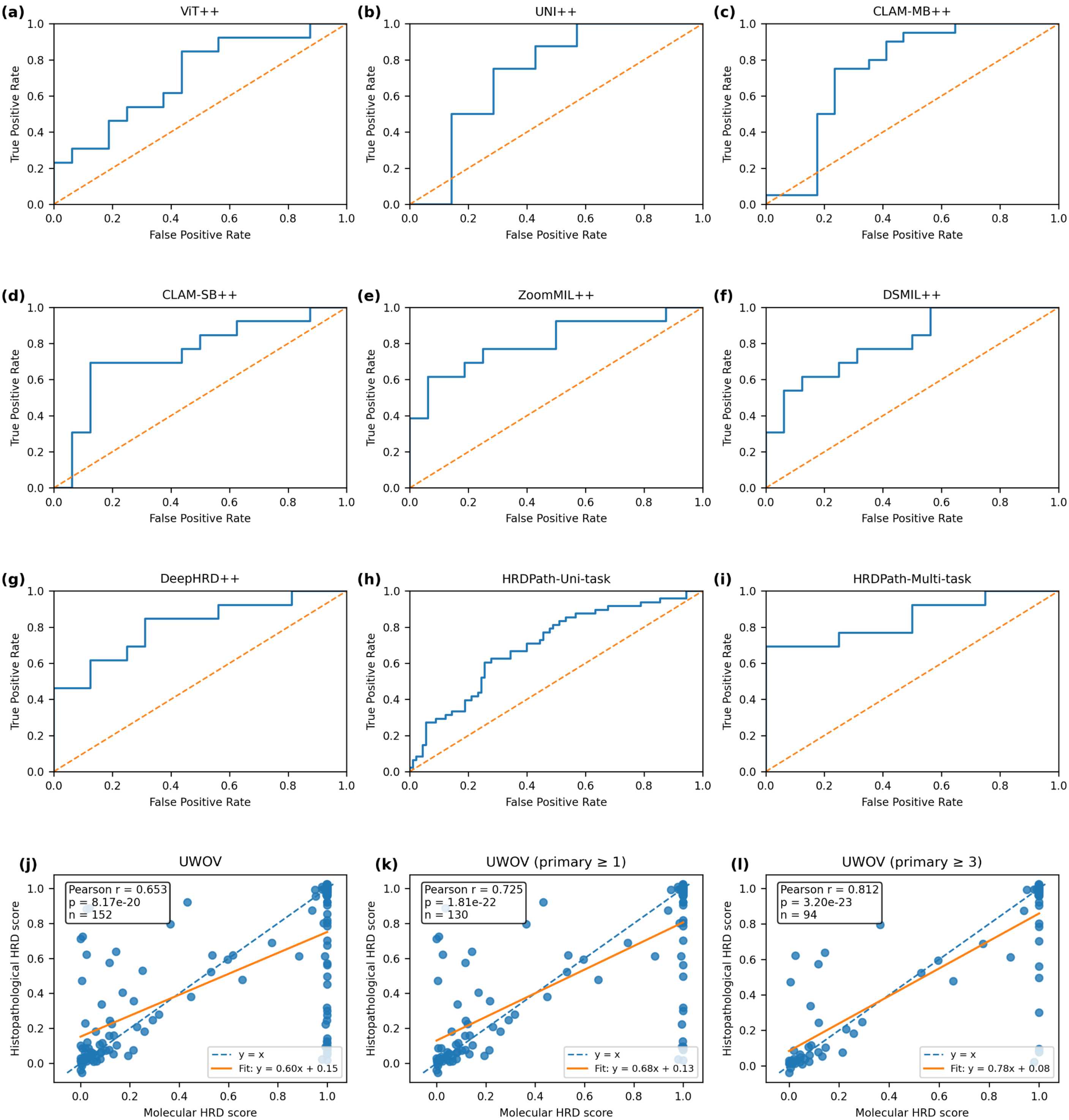

Supplementary Figure 2. Receiver operating characteristic (ROC) curves and score correlation analyses across cohorts. (a–i) ROC curves for the nine models evaluated on the UWOV test subset (n = 138 WSIs from 29 female patients, aged 38-83), corresponding to the models reported in Table 2. Each subplot reports the area under the curve (AUC). The binary HRD-low/HRD-high classification metrics in Table 2 were computed using a fixed probability threshold of 0.5 for all models, without cohort-specific or post hoc threshold optimisation. The ROC curves therefore illustrate the full sensitivity–specificity trade-off across all possible operating points for each model's predicted HRD probability. (j–l) Scatterplots comparing HRDPath-predicted histopathological HRD scores with ground-truth molecular HRD scores in UWOV, stratified by the number of available primary slides (n_primary) per patient: all slides considered (n=720 WSIs from 152 female patients, aged 18–86) (j), n_primary ≥ 1 (n=480 WSIs from 130 female patients, age 18–86) (k), and n_primary ≥ 3 (n=421 WSIs from 94 female patients, aged 18–86) (l). Correlation coefficients for each nested subset are reported in the legend. Dashed lines indicate the identity line (y = x), and the solid orange line indicates the overall linear regression fit. *P*-values in panels j–l were calculated using two-sided Pearson correlation tests. Multiple comparison correction was not applicable because these were correlation analyses. Source data are provided as a Source Data file.

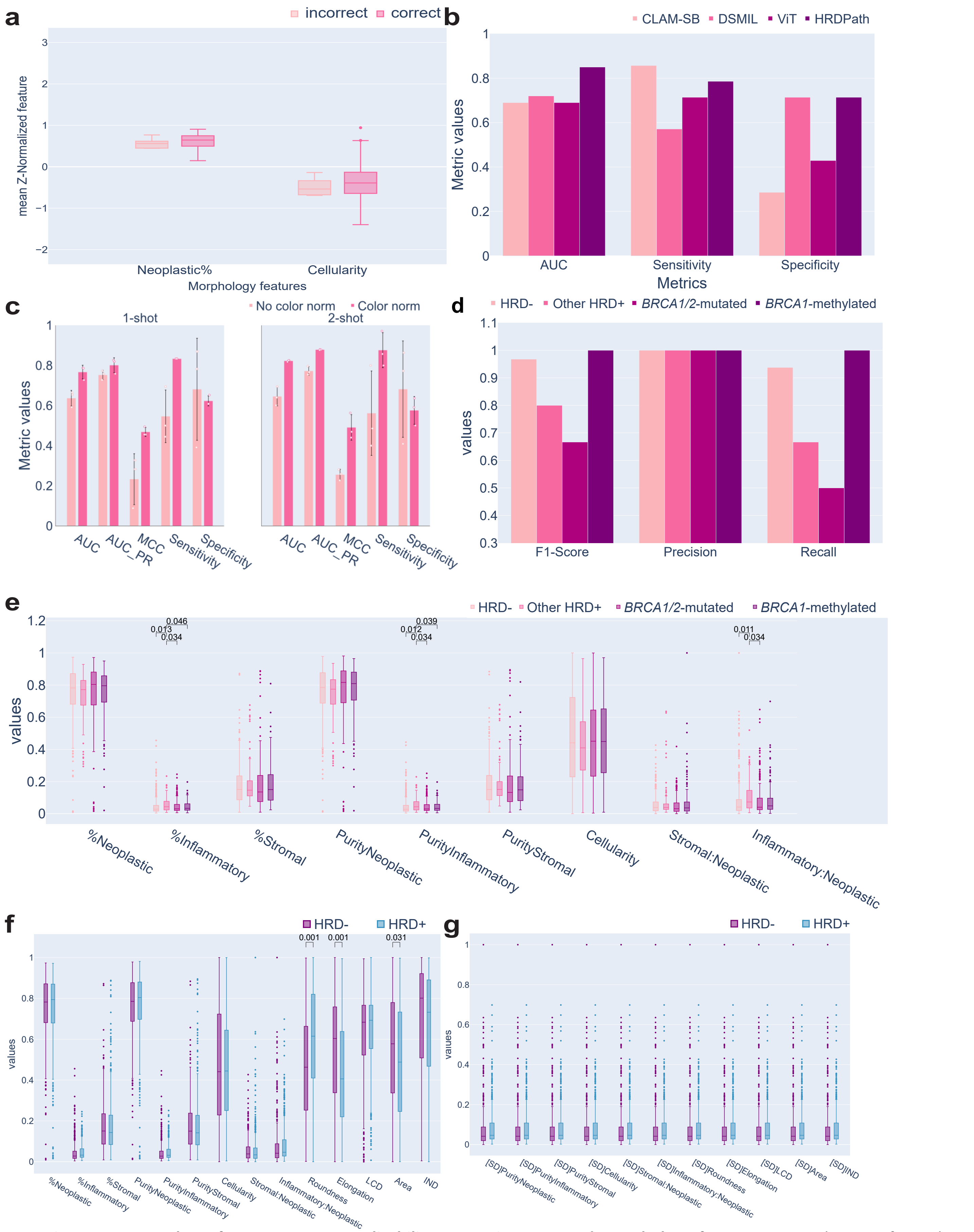

Supplementary Figure 3. HRDPath performance on generalizability, genomic status and morphology features a. Distribution of neoplastic percentage and cellularity across correct and incorrect HRDPath predictions in the full UWOV cohort (n = 720 WSIs from 152 female patients, aged 18–86). b. TCGAOV benchmark results in the test subset (n = 21 WSIs from 21 female patients, aged 37–85). c. Few-shot performance comparison evaluating the impact of colour-normalised input features in the TCGAOV cohort using 0.5 cutoff (n = 81 female patients, aged 37–85; three-fold cross-validation; CN, colour normalization； Error bars denote standard deviation across folds). d. HRDPath performance across genomic status groups (HRD−, n = 70; other HRD+, n = 17; *BRCA1/2*-mutated, n = 43; *BRCA1*-methylated, n = 22 female patients, aged 18–86). e-g. Distribution of average nuclear morphology per patient in tumor rich regions only across genomic status (n=152 female patients, aged 18–86; 720 slides; HRD-: 349; Other HRD+: 75; *BRCA1/2*-mutated:197; *BRCA1*-methylated: 99)(e), HRD status (f) and SD of nuclear morphology across HRD status (g). *P-values* in panels e-g were calculated with the two-sided *t*-test and adjusted for multiple testing using false discovery rate (FDR) correction. Only significant associations are shown. Source data are provided as a Source Data file.

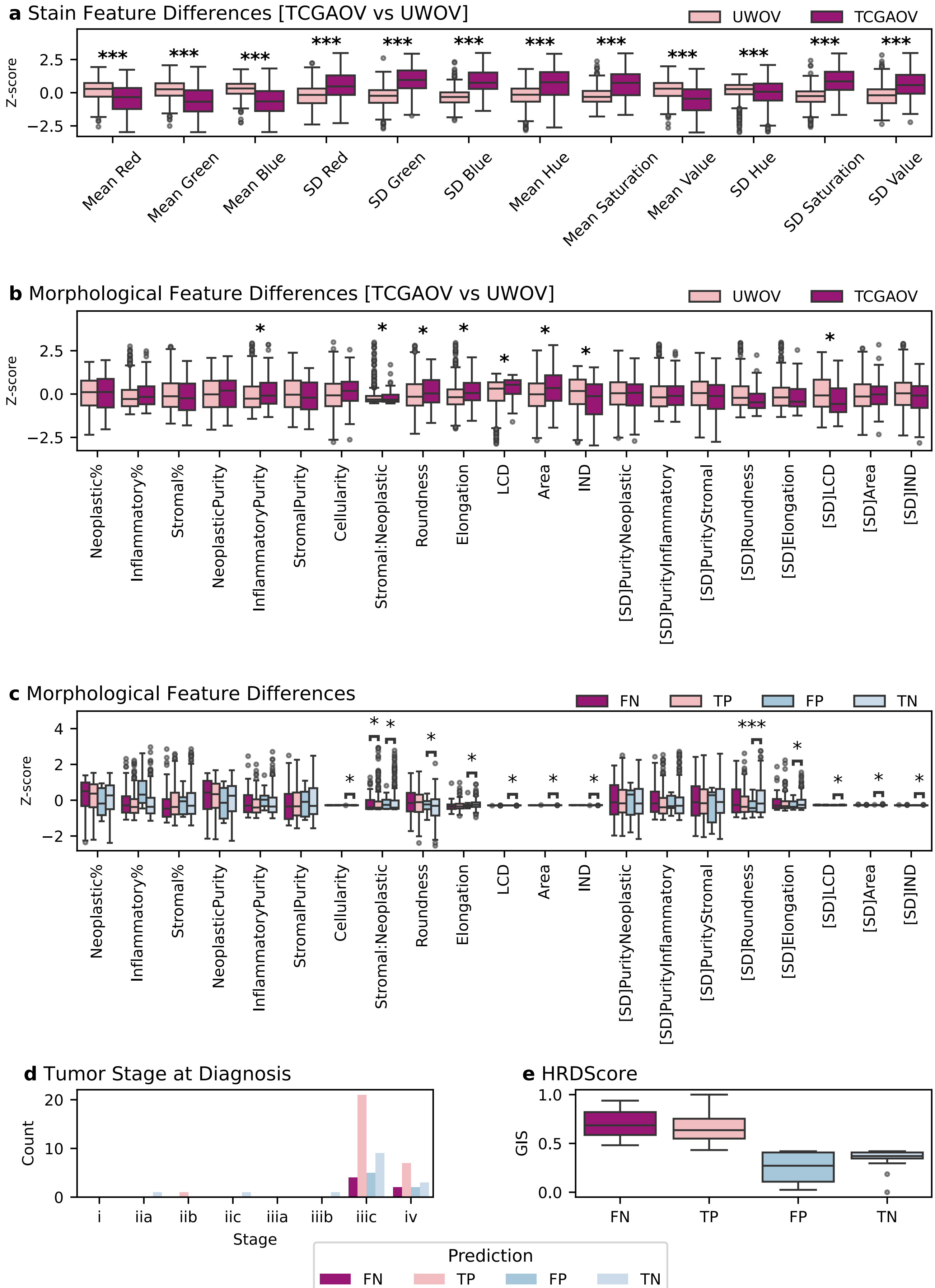

Supplementary Figure 4. Comparison of stain, morphological, and clinical features across datasets and prediction outcomes. a. Stain feature differences between TCGAOV (n=81 WSIs from 81 female patients, aged 37–85) and UWOV (n=720 WSIs from 152 female patients, aged 18–86) slides. b. Morphological feature differences between TCGAOV (n=81 WSIs from 81 female patients, aged 37–85) and UWOV (n=720 WSIs from 152 female patients, aged 18–86). c. Morphological differences between confusion groups on TCGAOV cohort (n=81 WSIs from 81 female patients, aged 37–85). d. Distribution of tumor stages at diagnosis between confusion groups, compared using a chi-squared test (n=81 WSIs from 81 female patients, aged 37–85). e. GIS (sum of LOH, TAI, LST) distribution across confusion groups. *P*-values in panels a–c, e were calculated using two-sided *t*-tests and adjusted for multiple testing using false discovery rate (FDR) correction. Statistical significance is annotated with asterisks according to the FDR-adjusted q < 0.05 (*), and q < 0.001 (***). Source data are provided as a Source Data file.

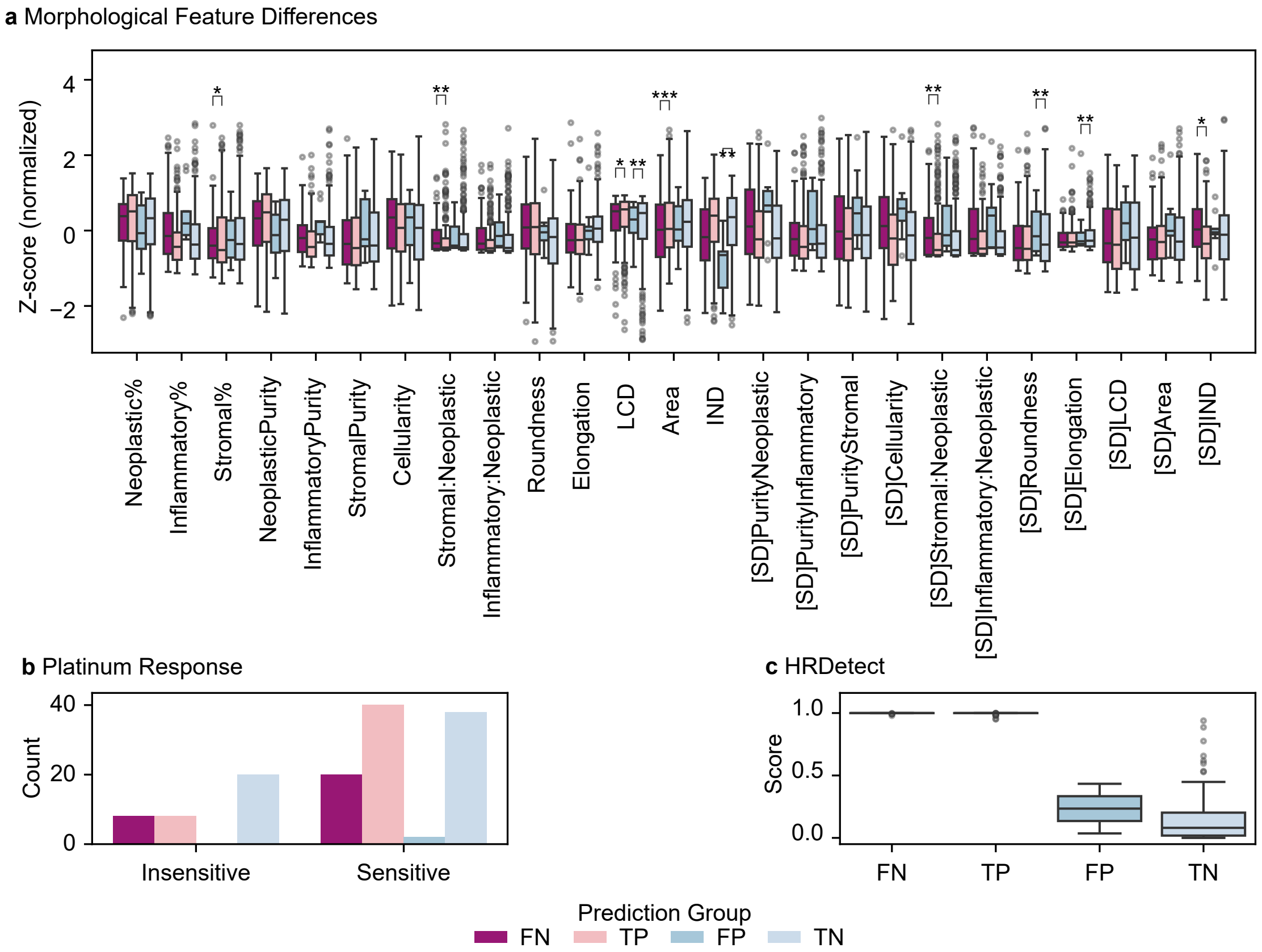

Supplementary Figure 5. Comparison between confusion groups on HRD predictions in the UWOV cohort across morphological, clinical, and molecular features. a. Z-score normalized morphological features show significant differences (n = 720 WSIs from 152 female patients, aged 18–86). b. Platinum sensitivity was more common in TP cases, though not statistically significant (NS, 137 female patients; 650 WSIs, aged 18-85). c. HRDetect scores across confusion groups (n = 720 WSIs from 152 female patients, aged 18–86). *P*-values in panels a, c were calculated using two-sided *t*-test and adjusted for multiple testing using false discovery rate (FDR) correction. *P*-values in panel b were calculated using two-sided Fisher's exact test. Statistical significance is annotated with asterisks according to the FDR-adjusted $q < 0.05$ (*), $q < 0.01$ (**); NS indicates not significant. Source data are provided as a Source Data file.

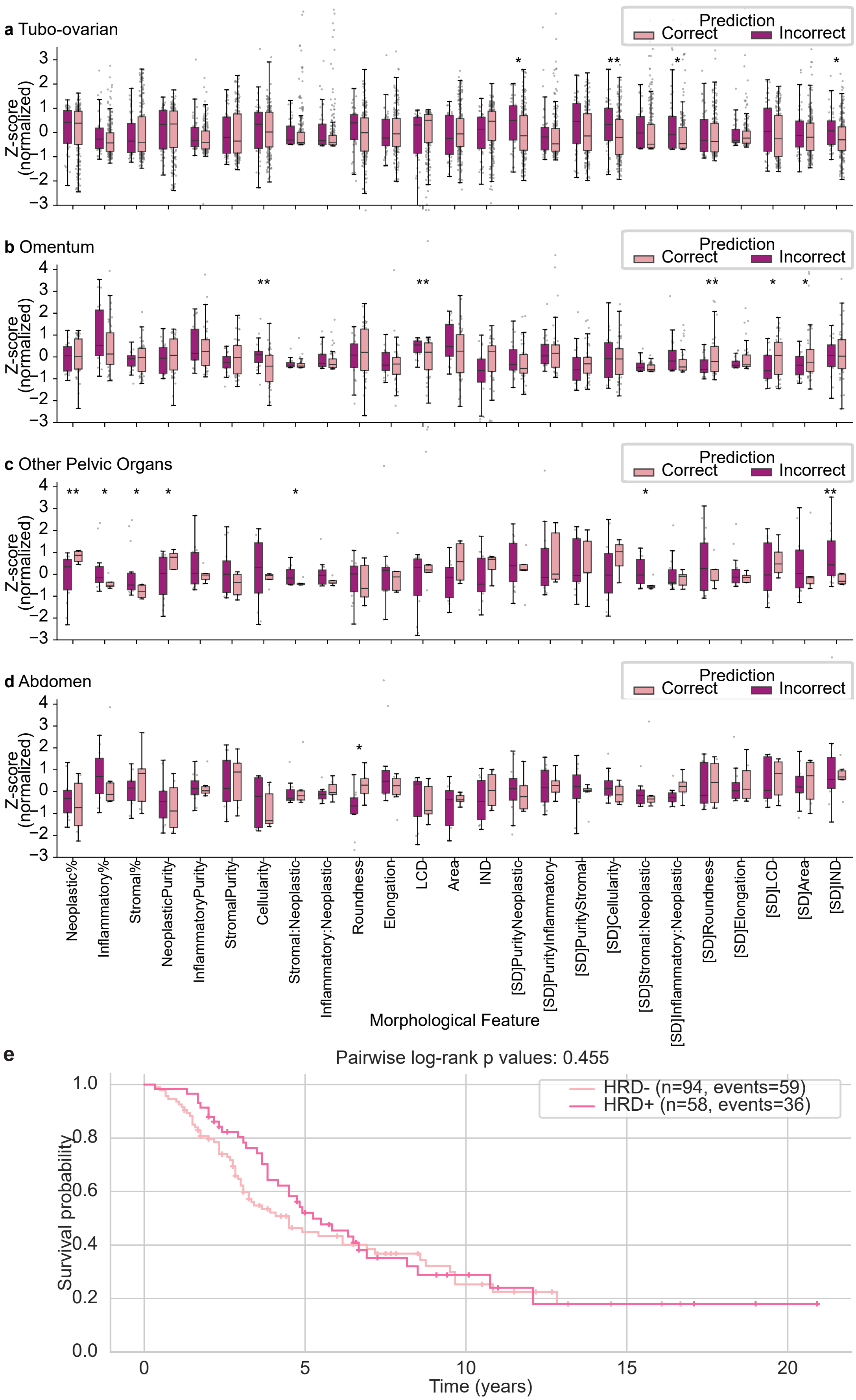

Supplementary Figure 6. Morphological differences between correctly and incorrectly predicted HRD-positive cases across anatomical locations. a-d. Box plots show z-score normalized values of selected morphological features in HRD-positive slides from four anatomical sites: tubo-ovarian (n=258 WSIs from 68 female patients, aged 37–85) (a), omentum (n=67 WSIs from 42 female patients, aged 39–82) (b), other pelvic organs (n=20 WSIs from 13 female patients, aged 48–68) (c), and abdomen (n=18 WSIs from 10 female patients, aged 39–82) (d). Cases are grouped by prediction outcome (correct vs. incorrect HRD status). Morphological features include cell-type composition (e.g., percentage of neoplastic, stromal, and inflammatory cells), cellularity, purity metrics, and spatial or structural variability descriptors. Due to the limited number of incorrect predictions, this analysis was considered exploratory and underpowered. e. Overall survival Kaplan-Meier analyses were performed on the UWOV cohort on HRDPath prediction (n = 720 WSIs from 152 female patients, aged 18–86). *P*-values in panels a-d were calculated with two-sided *t*-tests, and were not adjusted for multiple comparisons; panel e was calculated using two-sided log-rank test. Statistical significance is annotated with asterisks according to $p < 0.05$ (*), $p < 0.01$ (**). None of the comparisons remained significant after multiple-comparison correction. All features were z-score normalized across the full dataset prior to comparison. Source data are provided as a Source Data file.

Supplementary Tables

Supplementary Table 1. Datasets statistics. Six patients in UWOV were excluded from the training and were retained for cohort description and site-specific analyses due to absence of tubo-ovarian and omentum slides.

	**UWOV**		**TCGA Ovarian**		**PTRC**
**Split**	Train	Test	Train	Test	All
**No. of Patients**	117	29	61	20	93
**No. of WSIs**	493	138	61	21	198
**HRD Positive Ratio**	55.6%	44.8%	62.3%	66.7%	94.6%
***BRCA*-alteration/HRD+**	81.5%	76.9%	9.8%	4.8%	-
**Median Age (years)**	62.0	60.0	63.7	59	64
**IQR Age (years)**	15.0	19.0	16.5	10.5	15
**Histology**					
**High grade serous (1st and 2nd grade)**	111	28	61	21	93
**Low grade serous**	2	0	0	0	-
**Mixed**	3	0	0	0	-
**Clear cell**	1	0	0	0	-
**Endometrioid**	0	1	0	0	-
**Anatomical location**					
**Tubo-Ovarian**	377	103	61	20	106
**Omentum**	116	35	0	0	92
**Other Pelvic Organ**	0	42	0	0	0
**Abdomen**	0	29	0	0	0
**Others**	0	18	0	0	0

Supplementary Table 2. Multi-task learning, patient-level benchmark results on TCGA. Source data are provided as a Source Data file.

Models	AUC	Sensitivity	Specificity
**CLAM-SB++**	0.694	0.857	0.286
**DSMIL++**	0.724	0.571	0.714
**ViT++**	0.694	0.714	0.429
**HRDPath**	0.846	0.786	0.714

Supplementary Table 3. Effect of number of slides fed per patient on HRDPath performance. Source data are provided as a Source Data file.

Slides per patient	n patients	Sensitivity	Specificity
**1**	152	0.659	0.886
**2**	148	0.696	0.913
**3**	132	0.725	0.968
**4**	103	0.76	0.962
**5**	73	0.763	1.000
**6**	48	0.913	1.000
**7**	28	1.000	1.000
**8**	20	1.000	1.000
**9**	9	1.000	1.000
**10**	3	1.000	1.000
**11**	1	1.000	N/A

Supplementary Table 4. Composition-stratified sensitivity and specificity under fixed slide budgets (k). Abbreviations: p denotes number of primary slides and m denotes number of metastatic slides. Source data are provided as a Source Data file.

k	Composition (p,m)	n (HRD+)	Sensitivity	n (HRD−)	Specificity
2	p=2, m=0	32	0.938	48	0.896
2	p=1, m=1	33	0.879	51	0.941
3	p=3, m=0	29	0.931	37	0.892
3	p=2, m=1	32	0.906	48	0.938
3	p=1, m=2	13	0.769	27	1.000
3	Diverse 3 locations	4	1.000	9	1.000

Supplementary Table 5. Our proposed multi-task learning and patient-level (multiple slide) approach significantly improves the performance of HRDPath and the baseline model. For baseline models, our adapted models are indicated by ++. Source data are provided as a Source Data file.

**Models**	**AUC**	**Sensitivity**	**Specificity**
**ViT**	0.682	0.458	0.744
**ViT++ (multi-task, multi-slide)**	0.707	0.385	0.813
**DSMIL**	0.751	0.479	0.844
**DSMIL++ (multi-task, multi-slide)**	0.808	0.538	0.875
**HRDPath (single-task, single slide)**	0.700	0.521	0.744
**HRDPath (single-task, multiple slide)**	0.760	**0.846**	0.688
**HRDPath (multi-task, multi-slide)**	**0.846**	0.692	**0.938**

Supplementary Table 6. HRDPath performance on TCGA (unseen data) when trained on TCGA or UWOV. Source data are provided as a Source Data file.

Train source	Test source	#Patients	AUC	Sensitivity	Specificity
**TCGA**	TCGA	21	0.847	0.786	0.714
**UWOV**	TCGA	21	0.735	0.857	0.714

Supplementary Table 7. Few-shot learning results of HRDPath when pretrained on UWOV and evaluated on TCGAOV or PTRC when color normalization was employed (Youden-optimal thresholds). Source data are provided as a Source Data file.

Dataset	N-shot (3 folds)	AUC	AUC_PR	MCC	Sensitivity	Specificity
TCGAOV	1	0.767 ± 0.035	0.801 ± 0.037	0.560 ± 0.024	0.944 ± 0.028	0.551 ± 0.050
TCGAOV	2	0.823 ± 0.004	0.878 ± 0.002	0.581 ± 0.031	0.981 ± 0.016	0.500 ± 0.000
PTRC	1	0.711 ± 0.011	0.974 ± 0.002	0.269 ± 0.025	0.803 ± 0.091	0.667 ± 0.115
PTRC	2	0.726 ± 0.020	0.977 ± 0.002	0.289 ± 0.053	0.815 ± 0.078	0.700 ± 0.173

Supplementary Table 8. Few-shot learning results of HRDPath when pretrained on UWOV and evaluated on TCGAOV when no color normalization was employed. Source data are provided as a Source Data file.

**N-shot (3 folds)**	**AUC**	**AUC_PR**	**MCC**	**Sensitivity**	**Specificity**
1	0.636 ± 0.039	0.753 ± 0.023	0.233 ± 0.127	0.546 ± 0.131	0.681 ± 0.255
2	0.645 ± 0.047	0.773 ± 0.021	0.255 ± 0.027	0.562 ± 0.211	0.681 ± 0.241

Supplementary Table 9. Inference results of HRDPath across different anatomical locations when trained on images from primary and/or omental neoplasms. Source data are provided as a Source Data file.

Site	#Patients	AUC	Sensitivity	Specificity
**tubo-ovarian +omentum**	29	0.846	0.692	0.938
**tubo-ovarian**	26	0.836	0.727	0.800
**omentum**	23	0.627	0.444	0.857
**other pelvic locations**	24	0.587	0.231	0.727
**abdomen**	19	0.911	0.300	0.889

Supplementary Table 10. Inference results of HRDPath-primary across different anatomical locations when trained on images from primary neoplasms only. Source data are provided as a Source Data file.

Site	#Patients	AUC	Sensitivity	Specificity
**tubo-ovarian**	26	0.890	0.545	0.933
**omentum**	84	0.660	0.688	0.577
**other pelvic locations**	24	0.552	0.231	0.818
**abdomen**	19	0.678	0.400	0.778

Supplementary Table 11. Classification Performance of HRDPath-BRCA in predicting *BRCA1/2* alterations to demonstrate transferability[1]. Source data are provided as a Source Data file.

Task	Subset	#Patients	Sensitivity	Specificity
BRCA1/2	UWOV Test (All alterations)	31	0.600	0.952
BRCA1/2	TCGAOV	48	0.767	0.442
BRCA1/2	*BRCA1/2*-(HRD-)	65	-	0.982
BRCA1/2	All *BRCA1*-methylated	19	0.910	-
BRCA1/2	All *BRCA1/2*-mutated	30	0.912	-

[1] Italic font denotes genomic alterations

Supplementary Table 12. Pearson correlation between individual morphology-derived features and the HRDPath-predicted HRDScore across anatomical subsets. Pearson correlation coefficients were calculated between each morphology derived feature and the HRDPath predicted HRDScore across Primary (n=480) and All (n=720). Statistical significance was assessed using two-sided Pearson correlation tests with FDR correction. Features with FDR-adjusted q < 0.05 are marked in bold text. Source data are provided as a Source Data file.

Morphology feature	Subset	Pearson r	FDR q
**Neoplastic elongation**	**Primary**	**−0.161**	**0.007**
**Neoplastic roundness**	**Primary**	**0.216**	**0.000**
**LCD**	**Primary**	**0.145**	**0.017**
[SD] Neoplastic roundness	Primary	−0.110	0.116
[SD]Neoplastic area	Primary	−0.090	0.201
**Neoplastic elongation**	**All**	**−0.118**	**0.006**
**Neoplastic roundness**	**All**	**0.142**	**0.001**
**LCD**	**All**	**0.183**	**0.000**
**[SD] Neoplastic roundness**	**All**	**−0.146**	**0.001**
**[SD]Neoplastic area**	**All**	**-0.131**	**0.003**

Supplementary Table 13. Ridge and logistic regression analysis on all morphological features and HRDetect score and HRD status respectively across anatomical location subsets (CV: 5-fold cross-validation). Source data are provided as a Source Data file.

Location	CV (Ridge) HRDetect	CV AUC HRD
All sites	-0.294	0.539
**Primary only**	**−0.294**	**0.593**

Supplementary Table 14. HRDPath performance in predicting HRD across different genomic alterations[2] .Source data are provided as a Source Data file.

HRD Category	#Patient	Accuracy	Correct Predictions
HRD-	70	0.957	67
BRCA1-methylated	22	0.773	17
BRCA1/2-mutated	43	0.628	27
Other HRD+ (no *BRCA* alterations)	17	0.647	11

[2] Italic font denotes genomic alterations

Supplementary Notes

Supplementary Note 1. Comparison against existing HRD prediction methods

HRDPath addresses limitations of previous approaches and improves performance. For example, Loeffler et al.[35] have demonstrated promising results in predicting HRD from H&E images in breast cancer with an AUC reaching 93%. However, their models only achieved AUC of 63% in ovarian cancer[35]. DeepHRD also demonstrated strong potential for predicting HRD from H&E images in breast cancer, reporting AUC values between 0.77 and 0.85[24]. However, its performance in ovarian cancer, where there is a pressing clinical need, was not evaluated with classification metrics. The absence of key performance metrics such as specificity and sensitivity limits the interpretability of these results. Zhang et al. reported an AUC of 0.76 for HRD prediction in ovarian cancer[36], but their approach required manual segmentation of tumor regions followed by a random forest classifier, which is resource-demanding, limiting the method's translatability.

Supplementary Note 2. Linear Probing Analysis of Pretrained Feature Representations

To further isolate the contribution of pretrained feature representations, we performed a slide-level linear probing analysis comparing UNI and RetCCL embeddings. Although UNI is pretrained on a substantially larger corpus and designed as a general-purpose histopathology foundation encoder, we sought to directly assess the linear separability of the extracted features for HRD prediction. For each WSI in the UWOV cohort (n = 720), patch-level embeddings were mean-pooled to obtain slide-level representations. A logistic regression classifier was then trained and evaluated using repeated stratified 80/20 train/test splits with 10 fixed random seeds. UNI and RetCCL were evaluated on identical data splits to ensure fair comparison. Across accuracy, AUC, and macro-F1 metrics, RetCCL features consistently outperformed UNI. In particular, RetCCL achieved higher macro-F1 scores (0.718 ± 0.154) compared with UNI (0.676 ± 0.136). These findings indicate that RetCCL embeddings are more linearly separable for the HRD prediction task. This quantitative evidence suggests that differences in downstream model performance, including those observed with CLAM and related approaches, may be attributable to variation in representation quality and task alignment of the pretrained encoders.

Supplementary Note 3. Validation of HRDPath on metastasis-only tissue slides

As the majority of HGSOC female patients present with metastasis, we evaluated HRDPath performance across different anatomical locations when trained on tubo-ovarian and omental slides. Using a fixed probability cutoff of 0.5, HRDPath performed best on images from the tubo-ovarian site with AUC of 0.836, specificity of 0.800, and sensitivity of 0.727 (Figure 2f). In contrast, considering only omental tumors reduced the performance, with an AUC of 0.627, specificity of 0.857, and sensitivity of 0.444. The abdominal metastatic site (19 female patients) demonstrated an AUC of 0.911, specificity of 0.889, and sensitivity of 0.300. Other Pelvic metastases (24 female patients) resulted in an AUC of 0.587, specificity of 0.727, and sensitivity of 0.231 (Figure 2f; Supplementary Table 9).

Next, we sought to determine whether HRD-associated signatures remained consistent when HRDPath was trained solely on primary tumor images, with metastatic tumor images completely excluded. Using the same fixed probability cutoff of 0.5, HRDPath-primary achieved an AUC of 0.891, specificity of 0.933, and sensitivity of 0.545 on tubo-ovarian images (Figure 2g; Supplementary Table 10). On omental images, the AUC was 0.660, specificity 0.577, and sensitivity 0.688. The abdomen and Other Pelvic sites had lower AUCs of 0.678 and 0.552, with specificity/sensitivity of 0.778/0.400 and 0.818/0.231, respectively. These findings confirm the robustness of the signatures identified by HRDPath across different anatomical locations and suggest that including omental slides in HRDPath's training improves the model performance, especially on other metastatic sites, by learning more diverse pathological features.

Supplementary Note 4. HRDPath can predict *BRCA1/2*-associated alterations

To evaluate HRDPath generalizability to other tasks, we finetuned HRDPath to predict *BRCA1/2*-associated alterations. We found that HRDPath-BRCA can predict *BRCA1/2*-associated alterations with an AUC of 0.800, specificity of 0.952 and sensitivity of 0.600 (Figure 2h, Supplementary Table 11 and Methods). This reinforces confidence in HRDPath predictive power and suggests that genomic alterations associated with HRD correlate with distinct morphological patterns in H&E images. For this experiment, all available *BRCA*-annotated cases were used for fine-tuning (n = 95), and performance was evaluated on an unseen held-out test set (n = 31).

Given the limited number of TCGAOV samples with *BRCA1/2* labels (48 patients), we performed few-shot learning to evaluate HRDPath-BRCA performance on independent cohort (Methods). HRDPath demonstrated reasonable performance (mean AUC 0.680 ± 0.070; AUC-PR 0.590 ± 0.040 across three folds). The TCGAOV results showed relatively high sensitivity for *BRCA*-positive cases but reduced specificity (Supplementary Table 11). However, we note that this *BRCA1/2* analysis is exploratory and intended to demonstrate transferability of learned representations. Larger, more balanced multi-cohort datasets will be required for definitive evaluation of *BRCA1/2* prediction performance. Importantly, predicting patients with *BRCA1/2* alterations offers additional utility beyond overall HRD status, where it can provide a potential surrogate for patient stratification and prioritization in clinical trials targeting *BRCA1/2*-related mechanisms.

Supplementary Note 5. The impact of morphological and genomic heterogeneity on HRDPath performance

To gain insight into HRDPath performance, we examined genetic and morphological factors associated with incorrect predictions across datasets and anatomical locations. We compared correct predictions: true positives (TPs) and true negatives (TNs) with incorrect predictions: false positives (FPs) and false negatives (FNs). We did not identify FDR-significant differences in HRD scores for FP or FN cases (Supplementary Figures 4e and 5c). Morphological analyses identified selected differences between FN and TN cases. FNs had a lower stromal proportion (two-sided *t*-test, Benjamini–Hochberg FDR-adjusted $q = 0.0343$) and a lower stromal-to-neoplastic ratio ($q = 0.0144$; Supplementary Figure 5a). Although FNs showed a higher neoplastic proportion in the unadjusted analysis ($P = 0.0195$), this difference did not remain significant after FDR correction ($q = 0.0722$). FP cases showed a non-significant tendency towards a higher stromal-to-neoplastic ratio than other groups; this observation should be interpreted cautiously because only three FPs were present in the UWOV cohort. These patterns are consistent with genomic heterogeneity contributing to prediction discordance. HRD− cancers had the highest accuracy overall (67/70, 0.957). Among HRD+ subgroups, *BRCA1*-methylated tumors had the highest accuracy (17/22, 0.773), whereas Other HRD+ tumors lacking *BRCA1/2* alterations had the lowest (11/17, 0.647; Supplementary Table 14). Dataset-associated morphological differences also remained after FDR correction in the TCGAOV-versus-UWOV analysis (Supplementary Figure 4b). Together with the limited number of TCGAOV tumors harboring *BRCA* mutations (n = 7; Supplementary Table 1), these differences may contribute to the higher sensitivity, but lower specificity observed in TCGAOV relative to UWOV. These analyses support genomic and morphological heterogeneity as plausible contributors to prediction discordance, rather than establishing a single causal mechanism.

Some of the morphological differences between correct and incorrect cases were shared across anatomical regions, but we observed that the stromal-to-neoplastic ratio was higher in neoplasms from Other Pelvic metastases with incorrect predictions (Supplementary Figure 6c). We also observed weaker morphological separability between correct and incorrect cases in omentum and other pelvic metastases, with only modest differences in neoplastic proportion and inflammatory-to-neoplastic spatial relationships ($p = 0.0146$; Supplementary Figure 6b–c). These results suggest that differences in tumor microenvironment could be the reason for lower performance of HRDPath in Other Pelvic metastases and we anticipate that including images from this metastatic location could ensure the model learn location-dependent differences.
