# Supplementary material for: HRDPath: an explainable multi-model deep learning architecture for predicting homologous recombination deficiency from histopathology images
Source: Nat Commun. 2026 Oct 21;17:10870. doi: 10.1038/s41467-026-78430-y (PMC13686980; doi:10.1038/s41467-026-78430-y)
Supplement: Supplementary file 2 [file 41467_2026_78430_MOESM2_ESM.pdf]

Description of Additional Supplementary Files

Supplementary Dataset 1. Merged statistical analyses for HRD status and mutation comparisons. Values are reported to three decimal places. (HRD-: 350 WSIs from 70 female patients; HRD+: 370 WSIs from 82 female patients). Calculated using two-sided t-tests with FDR correction, and cohen d for effect size. Abbreviations: [%]: percentage of cell type in the entire slide, [Purity]: percentage of cell type in each patch then averaged over per slide. [SD]: standard deviation, LCD: local neoplastic cell density in a patch, IND: mean distance of inflammatory cells to neoplastic cells in a patch.
