# Supplementary material for: HRDPath: an explainable multi-model deep learning architecture for predicting homologous recombination deficiency from histopathology images
Source: Nat Commun. 2026 Oct 21;17:10870. doi: 10.1038/s41467-026-78430-y (PMC13686980; doi:10.1038/s41467-026-78430-y)
Supplement: Supplementary file 3 [file 41467_2026_78430_MOESM4_ESM.pdf]

Corresponding author(s): Heba Sailem

Last updated by author(s): Sep 21, 2026

Reporting Summary

Nature Portfolio wishes to improve the reproducibility of the work that we publish. This form provides structure for consistency and transparency in reporting. For further information on Nature Portfolio policies, see our Editorial Policies and the Editorial Policy Checklist.

Statistics

For all statistical analyses, confirm that the following items are present in the figure legend, table legend, main text, or Methods section.

n/a	Confirmed	
☐	☒	The exact sample size (*n*) for each experimental group/condition, given as a discrete number and unit of measurement
☐	☒	A statement on whether measurements were taken from distinct samples or whether the same sample was measured repeatedly
☐	☒	The statistical test(s) used AND whether they are one- or two-sided<br>*Only common tests should be described solely by name; describe more complex techniques in the Methods section.*
☐	☒	A description of all covariates tested
☐	☒	A description of any assumptions or corrections, such as tests of normality and adjustment for multiple comparisons
☐	☒	A full description of the statistical parameters including central tendency (e.g. means) or other basic estimates (e.g. regression coefficient) AND variation (e.g. standard deviation) or associated estimates of uncertainty (e.g. confidence intervals)
☐	☒	For null hypothesis testing, the test statistic (e.g. *F*, *t*, *r*) with confidence intervals, effect sizes, degrees of freedom and *P* value noted<br>*Give P values as exact values whenever suitable.*
☐	☒	For Bayesian analysis, information on the choice of priors and Markov chain Monte Carlo settings
☐	☒	For hierarchical and complex designs, identification of the appropriate level for tests and full reporting of outcomes
☐	☒	Estimates of effect sizes (e.g. Cohen's *d*, Pearson's *r*), indicating how they were calculated

Our web collection on statistics for biologists contains articles on many of the points above.

Software and code

Policy information about availability of computer code

Data collection	Data used in this study were obtained from previously published and well-established cohorts, including TCGA, HRDetect, and PTRC. These datasets were generated as part of prior studies and routine clinical practice, and no new data were collected specifically for this work. Whole-slide images were accessed in accordance with the original data access and ethical approvals associated with each cohort.
Data analysis	Whole-slide image processing and analysis were performed using the open-source CLAM (Clustering-constrained Attention Multiple Instance Learning) pipeline. The pipeline was used for tiling, feature extraction. In addition, a custom colour normalisation procedure was implemented by the authors, as described in the Methods section. All analyses were conducted using Python, with machine learning models implemented using PyTorch. Further details of the analysis workflow and software versions are provided in the Methods section.

For manuscripts utilizing custom algorithms or software that are central to the research but not yet described in published literature, software must be made available to editors and reviewers. We strongly encourage code deposition in a community repository (e.g. GitHub). See the Nature Portfolio guidelines for submitting code & software for further information.

Data

Policy information about availability of data

All manuscripts must include a data availability statement. This statement should provide the following information, where applicable:

- Accession codes, unique identifiers, or web links for publicly available datasets
- A description of any restrictions on data availability
- For clinical datasets or third party data, please ensure that the statement adheres to our policy

TCGAOV and PTRC-HGSOC datasets are publicly available from their respective repositories. The UWOV is available under restricted access due to patient privacy and consent restrictions. Access may be granted for ethically approved, bona fide research projects that are consistent with the participants' consent and subject to approval by the study team and completion of any required data access agreements. Requests should be directed to the corresponding author, who will aim to respond within 4 weeks. If access is approved, the data will be made available for the duration specified in the approved project/data access agreement. No new data were generated for this study. Source data are provided with this paper as Source Data file. The HRD scores were provided by Banda et al for use in the present study and included in the Source Data file. The underlying genomic data from which these scores were derived were not accessed nor used in this study and can be requested by contacting the authors of the original study49.

The TCGA-OV whole-slide images and genomic data are available through the Genomic Data Commons (https://portal.gdc.cancer.gov/), and PTRC-HGSOC is available through TCIA (https://www.cancerimagingarchive.net/collection/ptrc-hgsoc/). The TCGA-OV HRD scores were not generated or collected by the authors; they were obtained from Kang et al. (2022; https://doi.org/10.1177/03000605221133655) and are publicly available from the Genomic Data Commons (https://api.gdc.cancer.gov/data/66dd07d7-6366-4774-83c3-5ad1e22b177e).

Research involving human participants, their data, or biological material

Policy information about studies with human participants or human data. See also policy information about sex, gender (identity/presentation), and sexual orientation and race, ethnicity and racism.

Reporting on sex and gender	This study focused on ovarian cancer, a disease that occurs exclusively in individuals with female biological sex. As such, all samples included in the study were derived from female patients. Sex was considered in the study design through the inclusion of patients recorded as female in the clinical record. Sex- and gender-based comparisons were not applicable and were not performed. This is stated in accordance with journal guidelines.
Reporting on race, ethnicity, or other socially relevant groupings	Information on race, ethnicity, or other socially relevant groupings was not available in the primary dataset (UWOVHRD) used in this study. As such, these variables were not included or controlled for in the analyses.
Population characteristics	Relevant population characteristics are described in the Results, Methods, and supplementary table section.
Recruitment	All recruitment details are provided in this study. The analyses were based on secondary use of existing datasets obtained from previously established cohort
Ethics oversight	Ethical approval for data collection and research use in the original study12 was obtained from the University of Washington Human Subjects Division Institutional Review Board under approvals IRB 2872 and IRB 2802. All participants provided written informed consent before enrolment. The study design was approved by the King's College London Research Ethics Committee (LRM-24/25-36190). All data analyzed in this study were anonymized, and no identifiable personal information was available to the researchers.

Note that full information on the approval of the study protocol must also be provided in the manuscript.

Field-specific reporting

Please select the one below that is the best fit for your research. If you are not sure, read the appropriate sections before making your selection.

☒ Life sciences ☐ Behavioural & social sciences ☐ Ecological, evolutionary & environmental sciences

For a reference copy of the document with all sections, see nature.com/documents/nr-reporting-summary-flat.pdf

Life sciences study design

All studies must disclose on these points even when the disclosure is negative.

Sample size	Sample size was determined by data availability from the included cohorts. The study included a total of 326 patients across three independent cohorts (n = 152, 81, and 93). No formal a priori sample size calculation was performed, as this work involved retrospective analysis of existing datasets.
Data exclusions	No data were excluded from the analyses.
Replication	Model performance was evaluated using predefined and fixed training, and test splits. Replication was achieved through evaluation across independent cohorts, as described in the Methods section.

Randomization	Randomization was not applicable to this study, as no experimental allocation of participants or samples to groups was performed. Model evaluation was conducted using predefined and fixed data splits.
Blinding	Blinding was not applicable to this study, as all analyses were performed on de-identified data and did not involve subjective outcome assessment.

Reporting for specific materials, systems and methods

We require information from authors about some types of materials, experimental systems and methods used in many studies. Here, indicate whether each material, system or method listed is relevant to your study. If you are not sure if a list item applies to your research, read the appropriate section before selecting a response.

Materials & experimental systems

n/a	Involved in the study
☒	☐ Antibodies
☒	☐ Eukaryotic cell lines
☒	☐ Palaeontology and archaeology
☒	☐ Animals and other organisms
☐	☒ Clinical data
☒	☐ Dual use research of concern
☒	☐ Plants

Methods

n/a	Involved in the study
☒	☐ ChIP-seq
☒	☐ Flow cytometry
☒	☐ MRI-based neuroimaging

Clinical data

Policy information about clinical studies
All manuscripts should comply with the ICMJE guidelines for publication of clinical research and a completed CONSORT checklist must be included with all submissions.

Clinical trial registration	Not applicable. This study did not involve a clinical trial or prospective intervention and therefore was not registered.
Study protocol	Not applicable. This study involved secondary analysis of previously collected and de-identified data and did not involve a prospectively defined clinical study protocol.
Data collection	Not applicable. No new clinical data were collected for this study. All analyses were based on existing datasets obtained from previously established cohorts.
Outcomes	Not applicable. This study did not define or assess clinical trial outcomes. Model performance metrics and analytical endpoints are described in the Methods section.

Plants

Seed stocks	N/A
Novel plant genotypes	N/A
Authentication	N/A
