# Supplementary material for: HRDPath: an explainable multi-model deep learning architecture for predicting homologous recombination deficiency from histopathology images
Source: Nat Commun. 2026 Oct 21;17:10870. doi: 10.1038/s41467-026-78430-y (PMC13686980; doi:10.1038/s41467-026-78430-y)
Supplement: Supplementary file 4 [file 41467_2026_78430_MOESM5_ESM.pdf]

nature portfolio

Peer Review File

HRDPath: An Explainable Multi-Model Deep Learning Architecture for Predicting Homologous Recombination Deficiency from Histopathology Images

Corresponding Author: Dr Heba Sailem

This file contains all reviewer reports in order by version, followed by all author rebuttals in order by version.

Version 1:

Reviewer comments:

Reviewer #1

(Remarks to the Author)
This study proposes a novel deep learning model, HRDPath, that predicts homologous recombination deficiency (HRD) using histopathologic images. HRDPath utilizes an end-to-end learning approach and incorporates a multi-model architecture combining a Vision Transformer and DSMIL. The model achieved improved performance compared to previously published models, with an AUC of 0.846 and specificity of 0.938. Furthermore, the model demonstrated high generalizability by maintaining strong performance across two independent cohorts. The authors also attempted to explain the model's decision-making process using an attention-based explainable methods.
One limitation is that HRDetect, not an FDA-approved assay, was used as the ground truth in the training dataset. However, I consider that this limitation appears to be mitigated by the model's high performance when tested on TCGA-OV and PTRC-HGSOC datasets, in which HRD status was determined using the myChoice HRD.
In addition, the number of HRD-negative patients was extremely small (n=5) in the PTRC-HGSOC dataset, which limits the interpretation of specificity and PPV. Nevertheless, since publicly available ovarian cancer datasets that include both WSIs and HRD status are very limited, this constraint seems unavoidable. Future multi-institutional studies will be essential to further validate and enhance the model's generalizability.
However, several issues still should be addressed for acceptance:
Major Concerns
1. For a model intended as a screening tool, achieving a high PPV is crucial. Although the model demonstrates high specificity, the relatively low sensitivity is a significant limitation, as it increases the risk of missing HRD-positive patients who may potentially benefit from targeted therapies. The authors should further discuss how this limitation affects the model's clinical applicability and how HRDPath may be positioned as a surrogate biomarker in actual clinical practice.
2. The BRCA1/2 mutation prediction performance was evaluated in only 31 patients. It is unclear why the analysis was limited to this subset rather than including all patients in each cohort. Additional details or results regarding the performance across the entire three cohorts should be provided.
3. Interpretation of VAttentionCluster Results
The study demonstrates that most attention-highlighted regions correspond to tumor areas. Then, the authors interpreted the VAttentionClusters 5–7, containing fibrosis or immune infiltration, as a reflective of HRD biophenotypes. However, based on the presented images, these clusters seem to be composed exclusively of non-tumor regions, distant from tumor regions, rather than cancer-associated tissues, raising concerns about potential overinterpretation.
4. HRD status is also a well-established prognostic biomarker in ovarian cancer. However, this study only provides stratified analysis based on HRDPath/HRD concordance. It would be highly informative to include Kaplan–Meier survival curves comparing HRDPath-positive vs. HRDPath-negative groups, as well as HRD-positive vs. HRD-negative groups, in supplementary data.
5. Please specify the exact number of patients in the UWOV test cohort who had six or more WSIs. In addition, information regarding the distribution of slide numbers per patient in the training cohort should be provided.
6. The study concludes that six or more slides are sufficient for high prediction accuracy. However, in current clinical practice, an increasing proportion of ovarian cancer patients undergo diagnostic laparoscopy followed by neoadjuvant chemotherapy, resulting in limited tissue sampling. Could sampling tissue from anatomically diverse regions during diagnostic laparotomy enhance the predictive accuracy of the model, even when the number of slides is limited?

Minor Comments
• In Figure 3d, the letters labeling each image should be positioned lower.
• In Extended Data Figure 3d, the stage labels such as “i” and “ii” should be capitalized.

(Remarks on code availability)

Reviewer #2

(Remarks to the Author)
I would like to congratulate the authors for having developed the best histopathological predictor of HRD in ovarian cancer so far. But there are a number of major concerns which need to be addressed:
1. As reporting specificity without the corresponding sensitivity is not meaningful, the authors should always report the two measures together.
2. In Table 2, the authors compare the performance of nine models. How were the cutpoints for classification in HRD low and high defined? Additional to AUC, sensitivity, and specificity, the corresponding ROC curves should be shown to visualize the balance between sensitivity and specificity in more detailed.
3. The result of the HRDetect algorithms is a score (interpretable as probability) and a trinary classification (high/intermediate/low). How are the patients with intermediate HRD score treated during training?
4. Additional to the HRD calls, the correlation of the continuous HRD scores (histopathology vs. molecular) should be shown and analyzed.
5. At the end of the introduction the authors write “patches that were converted into feature vectors using CLAM”. Actually, CLAM uses ResNet that is pretrained on ImageNet as standard feature extractor. In the Methods section the authors state that they used RetCCL extract features. Please clarify, as it important if the feature extractor was pretrained on natural images or histopathological images.
6. UNI was pretrained on 100,0000, while RetCLL was pretrained on 22,000 histopathological WSI. How can it happen that CLAM with RetCLL outperformed UNI in the HRD classification task? Are there other differences between the two classifiers besides the different feature extractors?
7. It would be nice to see a systematic ablation analysis to understand which features of HRDPath contribute to outperforming other models.
8. Figure 3 (morphological differences): The p-values should be corrected for multiple hypothesis testing.
9. Figure 3: The sample sizes (e.g. the number of cells) are very large, but the effect sizes are very small. How much of the total prediction power of HRDPath can be attributed to the analyzed morphological variables? The distributions (medians and boxes) of the variables look virtual identical in HRD+ and HRD- cancers. Thus, the classification power of each of the variables is expected to be very weak.
10. Figure 6 g/h (Kaplan-Meier analyses): Please add the information which of the cohorts is analyzed here. How were the patients treated? In particular: Did the HRD+ patients receive PARR inhibitors? The results of the Kaplan-Meier analysis must be interpreted in the context of the therapies administered.
11. The total objective function is a sum of a loss function for continuous prediction and a loss function for class prediction. How was the relative weighting of these two parts of the loss function chosen? Is this choice optimal for the classification problem under consideration?
12. The conclusion (Discussion section) that the morphological differences between HRD+ and HRD- cancer could be attributed to the EMT is not supported very good by the data, as the effect sizes are very small.
13. The authors conclude (end of Discussion section) that HRDPath has the potential to be translated into a digital biomarker for HRD detection in ovarian cancer. Does HRDPath have the potential to by improved by being trained on larger data sets or in a different manner? The specificity of 94% could be sufficient for clinical utility, but a sensitivity of 69% appears much too low.

(Remarks on code availability)

Version 2:

Reviewer comments:

Reviewer #1

(Remarks to the Author)
The authors have satisfactorily addressed my comments and improved the clarity and rigor of the manuscript. I have no further major concerns.

(Remarks on code availability)

Reviewer #2

(Remarks to the Author)
The authors have addressed most of my comment, but two concerns remain:

1. Related to my original concern 2 and Suppl. Fig 2:
The AUC curves displayed appear as two straight lines with different slopes that are connected at the sensitivity-specificity point reported in Table 2. Thus, the ROC curves and probably (and the reported AUCs) seem to belong to a binary classification. However, it would be important and interesting to see the AUC curves for the continuous HRD score. Starting from this, trade-off between sensitivity and specificity and an optimization of the trade-off according to the clinical needs can be discussed.

2. Related to my original concern 4:
The correlations between the histopathological HRD score and the molecular HRD score should be shown in scatterplots for each of the cohorts. Such displays will provide additional insights: whether disagreements between the two scores occur for tumors close to the thresholds or if some of the tumors are having clearly high molecular HRD scores while having clearly low histopathological HRD scores or vice versa.

(Remarks on code availability)

Version 3:

Reviewer comments:

Reviewer #2

(Remarks to the Author)
The authors have satisfactorily addressed my two remaining comments.

(Remarks on code availability)

Open Access This Peer Review File is licensed under a Creative Commons Attribution 4.0 International License, which permits use, sharing, adaptation, distribution and reproduction in any medium or format, as long as you give appropriate credit to the original author(s) and the source, provide a link to the Creative Commons license, and indicate if changes were made.
In cases where reviewers are anonymous, credit should be given to 'Anonymous Referee' and the source.
The images or other third party material in this Peer Review File are included in the article's Creative Commons license,

unless indicated otherwise in a credit line to the material. If material is not included in the article's Creative Commons license and your intended use is not permitted by statutory regulation or exceeds the permitted use, you will need to obtain permission directly from the copyright holder.
To view a copy of this license, visit https://creativecommons.org/licenses/by/4.0/

Remarks to reviewers

Note: All changes to submission files are highlighted in red.

Reviewer #1 (Remarks to the Author):

This study proposes a novel deep learning model, HRDPath, that predicts homologous recombination deficiency (HRD) using histopathologic images. HRDPath utilizes an end-to-end learning approach and incorporates a multi-model architecture combining a Vision Transformer and DSMIL. The model achieved improved performance compared to previously published models, with an AUC of 0.846 and specificity of 0.938. Furthermore, the model demonstrated high generalizability by maintaining strong performance across two independent cohorts. The authors also attempted to explain the model's decision-making process using an attention-based explainable methods.

We thank the reviewer for their positive assessment and for recognizing the significant contribution of HRDPath in term of its performance, generalizability, and explainability.

One limitation is that HRDetect, not an FDA-approved assay, was used as the ground truth in the training dataset. However, I consider that this limitation appears to be mitigated by the model's high performance when tested on TCGA-OV and PTRC-HGSOC datasets, in which HRD status was determined using the myChoice HRD.

In addition, the number of HRD-negative patients was extremely small (n=5) in the PTRC-HGSOC dataset, which limits the interpretation of specificity and PPV. Nevertheless, since publicly available ovarian cancer datasets that include both WSIs and HRD status are very limited, this constraint seems unavoidable. Future multi-institutional studies will be essential to further validate and enhance the model's generalizability.

We appreciate the reviewer's recognition of the inherent challenges regarding data access and thank them for acknowledging our mitigation strategies. Indeed, the scarcity of publicly available balanced HGSOC datasets that include both high-quality whole-slide images and confirmed HRD status remains a significant constraint in the field. The publication of this manuscript will provide a strong foundation for future multi-institutional studies, which we are already considering as a next step.

Major Concerns

1. For a model intended as a screening tool, achieving a high PPV is crucial. Although the model demonstrates high specificity, the relatively low sensitivity is a significant limitation, as it increases the risk of missing HRD-positive patients who may potentially benefit from

targeted therapies. The authors should further discuss how this limitation affects the model's clinical applicability and how HRDPath may be positioned as a surrogate biomarker in actual clinical practice.

Response:

We agree that sensitivity is a critical consideration when identifying patients who may benefit from targeted therapies and lack of sensitivity assessment is a major limitation of previous studies.

To mitigate the limited sensitivity and support clinical translation, we propose to optimize the model performance by establishing minimum performance thresholds based on available diagnostic input. For instance, our model achieves a sensitivity of 0.910 and a specificity of 1.000 when 6 slides are used (Supplementary Table 3). Moreover, when using two or more WSI from primary site, we obtain an average sensitivity of 0.930 and an average specificity of 0.890 (Supplementary Table 4). Additionally, our consistency measure can provide clinicians with an estimate of model confidence, providing actionable insights. We have now updated the results section "Evaluation of HRDPath consistency and confidence to support clinical translation" to present these strategies. We further note that sensitivity is expected to improve with increased data availability and have clarified this point in the Discussion.

Revised text

Revised Results- Manuscript line137-161:

> "**Evaluation of HRDPath consistency and confidence to support clinical translation**
>
> As WSIs from multiple tumor locations are often acquired in HGSOC, we sought to evaluate HRDPath consistency when multiple slides are used per patient. We found that HRDPath predictions are generally consistent when using different slides from the same patient, and that integrating features from three or more slides can significantly improve model accuracy and consistency (Figure 2b–c and Methods). Importantly, when six or more slides were used, accuracy exceeded 0.950 with 0.910 sensitivity and 1.000 specificity (Figure 2b; Supplementary Table 3). To further assess whether anatomical diversity contributes to model performance beyond slide count, we evaluated all enumerated slide combinations at fixed sampling depths (two or three slides per patient), stratified by the number of primary metastatic WSIs, and diverse anatomy composition. When at least two primary-site slides were included, sensitivity and specificity remained high and stable regardless of whether additional slides originated from the primary site or other anatomical locations, indicating no independent performance gain from anatomical diversity under adequate primary sampling (0.906-0.938 sensitivity; 0.896-0.938 specificity; Supplementary Table 4). In contrast, when primary sampling was limited to a single slide, sensitivity

decreased while specificity remained high (0.769-0.869 sensitivity; 0.941-1.000 specificity; Supplementary Table 4). This is likely due to the fact that our training cohort is currently under-represented in metastatic samples, which are limited to omental sites and constitute only 23% of the total training data. When restricting analysis to patients sampled from three distinct anatomical locations, HRDPath achieved a sensitivity of 0.857 and a specificity of 1.000. However, we note that this analysis is based on data from 15 patients and needs to be expanded in future studies (Supplementary Table 4). This patient-level aggregation strategy can also be used to derive confidence score for model predictions by passing individual WSIs through the model and considering the prediction reliable only when at least 70% of slides yield consistent results. Collectively, these findings support the translational potential of HRDPath and underscore the utility of our patient-level approach in increasing the robustness and trustworthiness of model predictions."

Supplementary Table 3. Effect of number of slides fed per patient on HRDPath performance

Slides per patient	n patients	Sensitivity	Specificity
**1**	152	0.659	0.886
**2**	148	0.696	0.913
**3**	132	0.725	0.968
**4**	103	0.76	0.962
**5**	73	0.763	1.000
**6**	48	0.913	1.000
**7**	28	1.000	1.000
**8**	20	1.000	1.000
**9**	9	1.000	1.000
**10**	3	1.000	1.000
**11**	1	1.000	N/A

Supplementary Table 4. Composition-stratified sensitivity and specificity under fixed slide budgets (k). Abbreviations: p denotes number of primary slides and m denotes number of metastatic slides.

k	Composition (p,m)	n (HRD+)	Sensitivity	n (HRD−)	Specificity
2	p=2, m=0	32	0.938	48	0.896
2	p=1, m=1	33	0.879	51	0.941
3	p=3, m=0	29	0.931	37	0.892
3	p=2, m=1	32	0.906	48	0.938

3	p=1, m=2	13	0.769	27	1.000
3	Diverse 3 locations	7	0.857	8	1.000

Revised Discussion- manuscript line 454-465:

> "HRDPath operates at the patient level by aggregating information across multiple whole-slide images, aligning with standard pathological workflows involving multiple specimens per patient. Sensitivity increases substantially with the number of slides available, demonstrating that multi-slide aggregation improves confidence in HRD classification. To mitigate the limited sensitivity of HRDPath and support clinical translation, we propose to optimize by establishing minimum performance thresholds based on available diagnostic input. For instance, our model achieves a sensitivity of 0.910 and a specificity of 1.000 when 6 slides are used. When using two or more WSI from primary site, we obtain an average sensitivity of 0.930 and an average specificity of 0.890. Moreover, our consistency measure can provide clinicians with an estimate of model confidence, providing actionable insights. We anticipate that sensitivity and overall performance may be further improved through training on larger and more diverse datasets, incorporating alternative learning strategies such as self-supervised or weakly supervised pretraining, and integrating richer clinical or molecular annotations. "

Revised Discussion- manuscript line 466-475:

> "We believe that HRDPath has the potential to be translated into a digital biomarker for detecting HRD in ovarian cancer patients, integrating histopathology images with a transformer-based deep learning model for rapid assessment of genetic signatures. For example, to aid risk stratification, prioritize cases for molecular testing, or provide cost-effective decision support in settings where genomic assays are delayed or resource intensive. Our approach is the first to demonstrate significant improvement over existing methods and to predict HRD from metastatic tumors, thus expanding its potential clinical relevance beyond primary sites. Additionally, our morphological analysis uncovered previously unrecognized features of HRD+ HGSOC, offering insights into cancer evolution and tumor–microenvironment interactions. Together, these findings position HRDPath as a promising step toward image-based digital biomarkers for HRD."

2. The BRCA1/2 mutation prediction performance was evaluated in only 31 patients. It is unclear why the analysis was limited to this subset rather than including all patients in each

cohort. Additional details or results regarding the performance across the entire three cohorts should be provided.

Response:

BRCA1/2 prediction was evaluated on an unseen held-out test set (n = 31) following fine-tuning on all remaining 95 *BRCA*-annotated cases to assess generalization while avoiding data leakage, given the strong correlation between *BRCA* status and HRD labels. This split mirrors the UWOV HRD partition.

To further evaluate our HRD-BRCA model, we performed few-shot learning on datasets with available *BRCA1/2* status (TCGA-OV: 48 WSI, PTRC: no *BRCA1/2* labels available). HRDPath achieved a mean AUC of 0.68 ± 0.07 and AUC-PR of 0.59 ± 0.04 under a fixed-support few-shot setting, with higher sensitivity for BRCA-positive cases and reduced specificity (Supplementary Table 11). These results are now reported in the revised supplementary notes and Supplementary Table 10 to directly address performance under extreme label scarcity. We have clarified this experimental design and explicitly reframed the BRCA analysis as exploratory where the goal is to assess transferability of HRDPath rather than clinical readiness.

Revised Supplementary note 4 -line 48-67:

> **"Supplementary note 4: HRDPath can predict *BRCA1/2*-associated alterations**
>
> To evaluate HRDPath generalizability to other tasks, we finetuned HRDPath to predict *BRCA1/2*-associated alterations. We found that HRDPath-*BRCA* can predict *BRCA1/2*-associated alterations with an AUC of 0.80, specificity of 0.952 and sensitivity of 0.6 (Figure 2h, Supplementary Table 14 and Methods). This reinforces confidence in HRDPath predictive power and suggests that genomic alterations associated with HRD correlate with distinct morphological patterns in H&E images. For this experiment, all available *BRCA*-annotated cases were used for fine-tuning (n = 95), and performance was evaluated on an unseen held-out test set (n = 31).
>
> Given the limited number of TCGA-OV samples with *BRCA1/2* labels (48 patients), we performed few-shot learning to evaluate HRDPath-BRCA performance on independent cohort (Methods). HRDPath demonstrated reasonable performance (mean AUC 0.68 ± 0.07; AUC-PR 0.59 ± 0.04 across three folds), with relatively high sensitivity for *BRCA*-positive cases but reduced specificity (Supplementary Table 14). However, we note that this *BRCA1/2* analysis is exploratory and intended to demonstrate transferability of learned representations. Larger, more balanced multi-cohort datasets will be required for definitive evaluation of *BRCA1/2* prediction performance. Importantly, predicting patients with *BRCA1/2* alterations offer additional utility beyond overall HRD status, where it can provide a potential surrogate for patient stratification and prioritization in clinical trials targeting *BRCA1/2*-related mechanisms."

Supplementary Table 14. Classification Performance of HRDPath in predicting *BRCA1/2* alterations to demonstrate transferability

Task	Subset	#Patients	Sensitivity	Specificity
BRCA1/2	UWOV Test (All alterations)	31	0.600	0.952
BRCA1/2	UWOV *BRCA1/2*-(HRD-)	65	-	0.982
BRCA1/2	UWOV *BRCA1*-methylated	19	0.910	-
BRCA1/2	UWOV *BRCA1/2*-mutated	30	0.912	-
BRCA1/2	TCGAOV	48	0.767	0.442

3. Interpretation of VAttentionCluster Results

The study demonstrates that most attention-highlighted regions correspond to tumor areas. Then, the authors interpreted the VAttentionClusters 5–7, containing fibrosis or immune infiltration, as a reflective of HRD biophenotypes. However, based on the presented images, these clusters seem to be composed exclusively of non-tumor regions, distant from tumor regions, rather than cancer-associated tissues, raising concerns about potential overinterpretation.

Response:

Indeed, attention scores exhibit a moderate correlation with the neoplastic score (0.58-0.62, Fig. 3a), indicating significant agreement while also demonstrating that attention is not fully restricted to neoplastic regions. Consistent with this observation, VAttentionClusters 5–8 predominantly capture non-neoplastic regions, including stromal, fibrotic, adipose, and immune-rich tissue which could suggest that HRD+ is associated with changes in the tumor microenvironment. We have revised the Results and Discussion to explicitly distinguish tumor-intrinsic clusters (1–4) from microenvironmental clusters (5–8) and revised the results to reflect this distinction.

Revised Results- manuscript line 317-329:

> “Our VAttentionClusters revealed that HRDPath also identified several tumor-associated microenvironmental contexts associated with HRD status. Specifically,

VAttentionClusters 5-8 were characterized by low neoplastic cell proportions and cellularity but were dominated by necrotic, stromal, fibrotic, or adipose tissue regions (Figure 5). Through qualitative assessment, we found that VAttentionCluster 7 was mainly composed of adipose tissue and was significantly associated with HRD- cancers (Figure 6c). Conversely, VAttentionClusters 5 and 6 were primarily composed of stromal cells with high fibrosis present in VAttentionCluster 5 supported by its lower cellularity and high stromal proportion (Figure 5 and 6a). While these clusters were present in primary sites, VAttentionCluster 5 was also present in the omentum and abdomen metastases while VAttentionCluster 6 was present in Other Pelvic metastases (Figure 6e). VAttentionCluster 8, on the other hand, was characterized by infarcted tissue and fibrosis with the lowest cellularity and was enriched in both HRD- and Other HRD+ cancers (Figure 6a-d). These results suggest that HRD+ cancers display location-specific changes in the tumor microenvironment which can provide additional context for HRDPath predictions."

Revised Discussion- manuscript line 427-429:

"Our explainability pipeline can be potentially used to discover novel phenotypic differences but also advance our understanding of HRD alterations might shape the tumor microenvironment."

4. HRD status is also a well-established prognostic biomarker in ovarian cancer. However, this study only provides stratified analysis based on HRDPath/HRD concordance. It would be highly informative to include Kaplan–Meier survival curves comparing HRDPath-positive vs. HRDPath-negative groups, as well as HRD-positive vs. HRD-negative groups, in supplementary data.

Response:

Unexpectedly, we identified no association between HRDpath and overall survival, whereas HRDetect demonstrated a significant correlation. We have included the corresponding survival curves in the supplementary figures for reference (Supplementary Figure 5e). However, it is important to note that the primary objective of this study was to train a deep learning algorithm to predict HRD status. Therefore, the cohort was not powered sufficiently for a robust survival analysis, and these specific findings likely reflect the limited number of patients involved. The revised manuscript text and supplementary figures have been updated accordingly to reflect this.

Revised Results- manuscript line 343-355:

"**Association of HRDPath predictions and patient outcomes**

We found that differences in genomic alterations and morphological heterogeneity could partially explain discordant cases where image-based HRD prediction did not match genomic HRD status (Supplementary Note 5). We therefore investigated whether HRDPath could stratify patient outcomes using overall survival (OS). Interestingly, false positive cases (HRDPath+/HRD-) were associated with significantly worse survival in the integrated UWOV and TCGA cohorts when considering all available follow-up ($p \leq 0.01$, Figure 6g). Moreover, the false negative cases (HRDPath-/HRD+) showed significantly worse survival than the true positives and true negatives when considering patients with less than 5 years of survival only ($p \leq 0.05$ and Figure 6h). Surprisingly, we do not observe a significant association between OS and HRDPath predictions (Supplementary Figure 5e) which could be due to limited patient number. This suggests that image-based predictions by HRDPath capture clinically meaningful phenotypes not reflected in genomic-based HRD assays, highlighting potential for broader prognostic relevance."

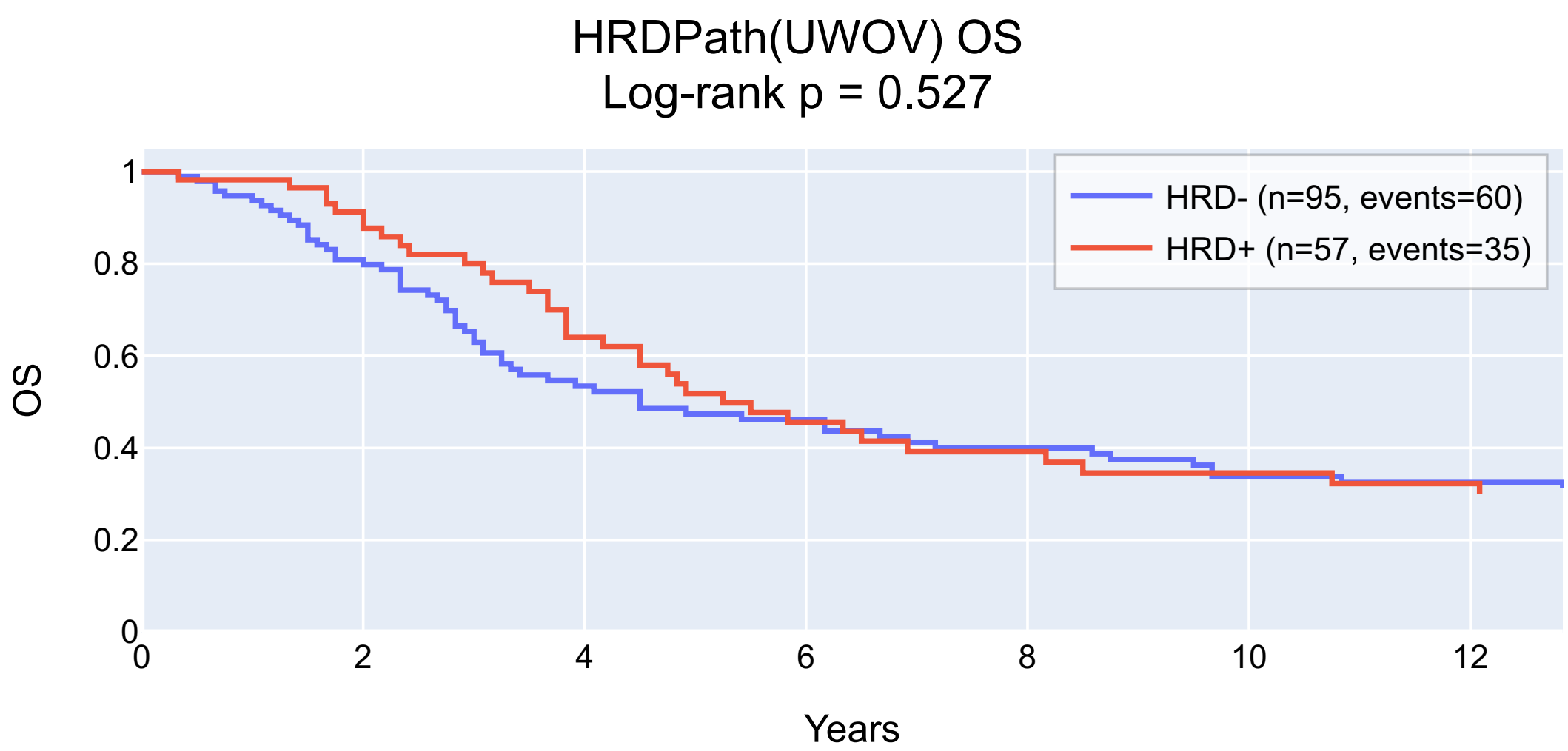

Supplementary Figure 5. e. Overall survival Kaplan-Meier analyses were performed on HRDPath prediction (UWOV cohort).

5. Please specify the exact number of patients in the UWOV test cohort who had six or more WSIs. In addition, information regarding the distribution of slide numbers per patient in the training cohort should be provided.

Response:

The distribution of the number of whole-slide images per patient is shown in Figure 1e and Supplementary Table 3. There are 48 patients with six or more slides. We have added a brief clarification in the manuscript to highlight this information more explicitly.

Revised text (Manuscript line 88-90):

"The number of slides per patient is comparable between HRD+ and HRD− groups (Overall average: 4.3 ± 2.19; HRD−: 4.99±2.07; HRD+: 4.52±2.12), where Figure 1e shows the distribution of the number of WSIs per patient across the cohort."

Supplementary Table 3. Effect of number of slides fed per patient on HRDPath performance

Slides per patient	n patients	Sensitivity	Specificity
**1**	152	0.659	0.886
**2**	148	0.696	0.913
**3**	132	0.725	0.968
**4**	103	0.76	0.962
**5**	73	0.763	1.000
**6**	48	0.913	1.000
**7**	28	1.000	1.000
**8**	20	1.000	1.000
**9**	9	1.000	1.000
**10**	3	1.000	1.000
**11**	1	1.000	N/A

6. The study concludes that six or more slides are sufficient for high prediction accuracy. However, in current clinical practice, an increasing proportion of ovarian cancer patients undergo diagnostic laparoscopy followed by neoadjuvant chemotherapy, resulting in limited tissue sampling. Could sampling tissue from anatomically diverse regions during diagnostic laparotomy enhance the predictive accuracy of the model, even when the number of slides is limited?

Response:

We appreciate the reviewer's suggestion. We evaluated sensitivity and specificity across varying availability of slides to address potential data limitations. We performed a composition-stratified analysis of sensitivity and specificity under fixed slide budgets (k = 2 and k = 3). Sensitivity and specificity were calculated at the patient level by aggregating predictions across all valid slide combinations of a given composition for each patient, thereby avoiding bias from unequal numbers of combinations per patient. This analysis shows that when at least two primary-site slides are available, both sensitivity and specificity reach 0.90, indicating model robustness. When sampling from primary tumor site is limited to a single slide, sensitivity decreases while specificity remains high, reflecting a conservative prediction behavior under slide-limited settings.

Revised Results- manuscript line 137-161:

"**Evaluation of HRDPath consistency and confidence to support clinical**

translation

As WSIs from multiple tumor locations are often acquired in HGSOC, we sought to evaluate HRDPath consistency when multiple slides are used per patient. We found that HRDPath predictions are generally consistent when using different slides from the same patient, and that integrating features from three or more slides can significantly improve model accuracy and consistency (Figure 2b–c and Methods). Importantly, when six or more slides were used, accuracy exceeded 0.950 with 0.910 sensitivity and 1.000 specificity (Figure 2b; Supplementary Table 3). To further assess whether anatomical diversity contributes to model performance beyond slide count, we evaluated all enumerated slide combinations at fixed sampling depths (two or three slides per patient), stratified by the number of primary metastatic WSIs, and diverse anatomy composition. When at least two primary-site slides were included, sensitivity and specificity remained high and stable regardless of whether additional slides originated from the primary site or other anatomical locations, indicating no independent performance gain from anatomical diversity under adequate primary sampling (0.906-0.938 sensitivity; 0.896-0.938 specificity; Supplementary Table 4). In contrast, when primary sampling was limited to a single slide, sensitivity decreased while specificity remained high (0.769-0.869 sensitivity; 0.941-1.000 specificity; Supplementary Table 4). This is likely due to the fact that our training cohort is currently under-represented in metastatic samples, which are limited to omental sites and constitute only 23% of the total training data. When restricting analysis to patients sampled from three distinct anatomical locations, HRDPath achieved a sensitivity of 0.857 and a specificity of 1.000. However, we note that this analysis is based on data from 15 patients and needs to be expanded in future studies (Supplementary Table 4). This patient-level aggregation strategy can also be used to derive confidence score for model predictions by passing individual WSIs through the model and considering the prediction reliable only when at least 70% of slides yield consistent results. Collectively, these findings support the translational potential of HRDPath and underscore the utility of our patient-level approach in increasing the robustness and trustworthiness of model predictions."

Supplementary Table 4. Composition-stratified sensitivity and specificity under fixed slide budgets (k). Abbreviations: p denotes number of primary slides and m denotes number of metastatic slides.

k	Composition (p,m)	n (HRD+)	Sensitivity	n (HRD−)	Specificity
2	p=2, m=0	32	0.938	48	0.896
2	p=1, m=1	33	0.879	51	0.941
3	p=3, m=0	29	0.931	37	0.892

3	p=2, m=1	32	0.906	48	0.938
3	p=1, m=2	13	0.769	27	1.000
3	Diverse 3 locations	7	0.857	8	1.000

Minor Comments

• In Figure 3d, the letters labeling each image should be positioned lower. (addressed)

• In Supplementary Figure 3d, the stage labels such as “i” and “ii” should be capitalized.

Addressed

Reviewer #2 (Remarks to the Author):

I would like to congratulate the authors for having developed the best histopathological predictor of HRD in ovarian cancer so far.

We thank the reviewer for their support and encouragement.

But there are a number of major concerns which need to be addressed:

1. As reporting specificity without the corresponding sensitivity is not meaningful, the authors should always report the two measures together.

Response:

We agree that specificity should be interpreted alongside sensitivity. In the revised manuscript, sensitivity and specificity are now reported jointly for all key evaluations.

2. In Table 2, the authors compare the performance of nine models. How were the cutpoints for classification in HRD low and high defined? Additional to AUC, sensitivity, and specificity, the corresponding ROC curves should be shown to visualize the balance between sensitivity and specificity in more detailed.

Response:

For all models in Table 2, HRD low/high classification was defined using a fixed probability threshold of 0.5, applied consistently without cohort-specific or post-hoc optimization. Sensitivity and specificity correspond to this operating point. To visualize the full sensitivity–specificity trade-off, ROC curves for all models have been added as Supplementary Figure 2 and mentioned in Results.

Revised Results- manuscript line 125-128:

*"*Incorporating a multi-task learning objective proved essential for achieving superior specificity with an absolute improvement of 0.25 and a higher overall performance, reflected by a 0.09 gain in AUC. However, this enhancement came at the cost of reduced sensitivity (Table 2; Supplementary Figure 2). "

Revised Results- manuscript line 163-166:

> "To rigorously evaluate HRDPath, we benchmarked its performance against widely used and recent deep learning models in histopathology using the same patient cohort. To ensure a fair comparison, all models were trained and tested using the same training/testing split, fixed 0.5 cut point and our proposed multi-task and patient-level learning framework."

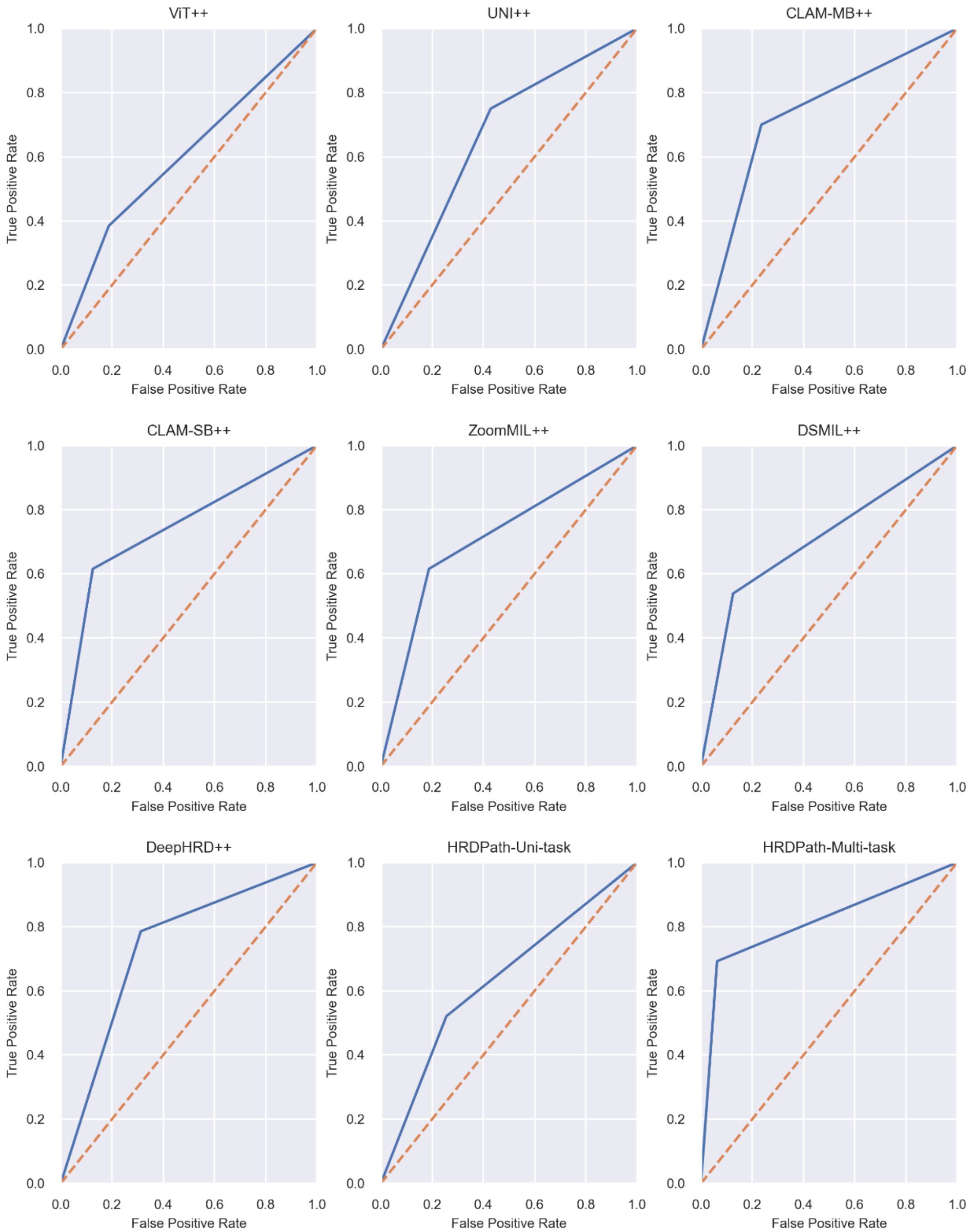

Supplementary Figure 2. Receiver operating characteristic (ROC) curves for all models evaluated in Table 2 based on Test subset.

3. The result of the HRDetect algorithms is a score (interpretable as probability) and a trinary classification (high/intermediate/low). How are the patients with intermediate HRD score treated during training?

Response:

HRDetect provides both a continuous score and a trinary classification. For training HRDPath, we adopted a binary definition, treating HRDetect-high cases as HRD-positive and grouping HRDetect-intermediate cases with HRDetect-low cases. This choice is based on that fact that clear HRD cases (i.e., BRCA mutated, BRCA1 methylated, RAD51C/D mutated) were all found in the HRDetect-high group. Therefore, for the purpose of training HRDPath, we only considered HRDetect high versus all others. The paper and analyses presenting HRDetect results is now in press in Clinical Cancer Research, and will be referenced in the final revision of this manuscript (also attached as related information).

Revised Methods- manuscript line 509-511:

> "For supervised learning, we adopted a binary HRD definition, grouping HRDetect-intermediate cases with HRDetect-low cases as HRD-negative in line with previous work (Clinical Cancer Research Paper will be referenced)."

4. Additional to the HRD calls, the correlation of the continuous HRD scores (histopathology vs. molecular) should be shown and analyzed.

Response:

We evaluated correlations between continuous HRDPath scores and molecular HRD reference scores. HRDPath predictions showed strong correlations with HRDetect scores in UWOV ($r = 0.627$, $p \leq 0.001$) and with myChoice genomic instability scores in TCGA-OV ($r = 0.735$, $p \leq 0.001$). These results have been clarified in the Results section.

Revised Results- manuscript Line 128-130:

> "Notably, predicted HRDPath score showed good correlation with HRDetect score in UWOV (Pearson correlation coefficient = 0.653, $p \leq 0.001$) and GIS score in TCGAOV (Pearson correlation coefficients = 0.625, $p \leq 0.001$) further supporting model robustness."

5. At the end of the introduction the authors write "patches that were converted into feature vectors using CLAM". Actually, CLAM uses ResNet that is pretrained on ImageNet as standard feature extractor. In the Methods section the authors state that they used RetCCL extract features. Please clarify, as it important if the feature extractor was pretrained on natural images or histopathological images.

Response:

We used the CLAM pipeline only for preprocessing (tiling, background filtering, normalization). Feature extraction was performed using RetCCL, a histopathology-pretrained encoder, rather

than CLAM's ImageNet-pretrained ResNet. The Introduction and Methods have been revised to clarify this distinction.

Revised Results- manuscript line 96-100:

> "Given the large size of WSI images, we extracted smaller patches that were processed following the CLAM[19] processing pipeline and encoded using a histopathology-pretrained RetCCL[20] feature extractor. We also performed color normalization to ensure transferability across datasets. This pipeline enables the efficient handling of gigapixel images into a series of deep learning-based feature vectors that are then used for the subsequent classification of HRD status. "

6a. UNI was pretrained on 100,0000, while RetCLL was pretrained on 22,000 histopathological WSI. How can it happen that CLAM with RetCLL outperformed UNI in the HRD classification task?

Response:

We also expected stronger performance given that UNI is pretrained on a substantially larger corpus and serves as a general-purpose histopathology foundation encoder. To further disentangle the effect of the feature representations themselves, we conducted a slide-level linear probing analysis using mean-pooled patch embeddings and logistic regression. Evaluation was performed using repeated stratified 80/20 train/test splits with 10 fixed random seeds on all 720 WSIs in the UWOV cohort, with UNI and RetCCL evaluated on identical splits. The results show that RetCCL features consistently achieved higher linear probing performance than UNI across accuracy, AUC, and macro-F1 (macro-F1: 0.718 ± 0.154 vs. 0.676 ± 0.136). This indicates that RetCCL representations are more linearly separable for the HRD prediction task, providing a quantitative explanation for why CLAM, and other methods, outperformed UNI when trained on RetCCL. This suggests that the observed performance difference could be attributable to differences in representation quality and task alignment of the pretrained features.

Revised Results- manuscript line 179-182:

> "Moreover, HRDPath outperformed ZoomMIL++, a hierarchical model that integrates features extracted at different image resolutions. Surprisingly, we observe that using the foundation model UNI++ as a feature extractor results in significantly lower performance which could be due to more complex embedding (Table 2, Supplementary Note 2)."

Added Supplementary Note 2- Supplementary Note line 13-26:

“**Supplementary note 2: Linear Probing Analysis of Pretrained Feature Representations**

To further isolate the contribution of pretrained feature representations, we performed a slide-level linear probing analysis comparing UNI and RetCCL embeddings. Although UNI is pretrained on a substantially larger corpus and designed as a general-purpose histopathology foundation encoder, we sought to directly assess the linear separability of the extracted features for HRD prediction. For each WSI in the UWOV cohort (n = 720), patch-level embeddings were mean-pooled to obtain slide-level representations. A logistic regression classifier was then trained and evaluated using repeated stratified 80/20 train/test splits with 10 fixed random seeds. UNI and RetCCL were evaluated on identical data splits to ensure fair comparison. Across accuracy, AUC, and macro-F1 metrics, RetCCL features consistently outperformed UNI. In particular, RetCCL achieved higher macro-F1 scores (0.718 ± 0.154) compared with UNI (0.676 ± 0.136). These findings indicate that RetCCL embeddings are more linearly separable for the HRD prediction task. This quantitative evidence suggests that differences in downstream model performance, including those observed with CLAM and related approaches, may be attributable to variation in representation quality and task alignment of the pretrained encoders.”

6b. Are there other differences between the two classifiers besides the different feature extractors?

Response:

Beyond feature extractors, the CLAM-based model incorporates a series of gated attention, UNI++ uses a single attention pooling layer while HRDPath adopts transformer’s multi-head self-attention module. These architectural differences could indeed contribute to performance differences.

7. It would be nice to see a systematic ablation analysis to understand which features of HRDPath contribute to outperforming other models.

To address the reviewer comment, we performed a systematic ablation study to evaluate the contribution of individual HRDPath components under identical experimental settings and added a new subsection titled “**Ablation analysis of HRDPath components**”. We also included our analysis of feature extractor contribution to this section.

Briefly we found that each HRDPath component addresses a distinct modelling need. ViT captures global slide-level morphology, while DSMIL performs discriminative instance selection to focus on sparse HRD-associated regions, improved sensitivity to 0.615 with the same specificity of 0.938 when integrating ViT and DSMIL compared to independent models. Local window construction (LWC), together with absolute positional encoding (APE), captures

spatially coherent local patterns that are not captured by instance selection alone, mimicking the sliding kernel mechanism of convolutional neural network. Consistent with this hypothesis, ablation results show that performance gains arise from the synergistic integration of LWC + APE rather than LWC alone (Table 3).

Added Results- manuscript line 213-228:

"**Ablation analysis of HRDPath components**

Having previously established the baseline performance of the individual ViT and DSMIL modules that comprise HRDPath, we conducted a series of multi-model variants under identical experimental settings to elucidate the contribution of different components (Table 2). Integrating global and instance-level representations (ViT + DSMIL) improved sensitivity and specificity balance but did not consistently enhance overall AUC, suggesting that fusion of global and instance features alone is not sufficient. We observed that introducing the proposed local window construction (LWC) without absolute positional encoding (APE) module did not lead improvement in performance (AUC = 0.745). While using LWC and APE together, we observed a significant gain (AUC=0.846). We attribute this gain to the fact that local window construction, together with APE, imposes a CNN-like inductive bias by restricting attention to spatially proximate regions and explicitly encoding relative spatial relationships. This localized inductive structure enhances the model's ability to capture spatially coherent morphological patterns that are critical for HRD prediction. These findings demonstrate that HRDPath's performance improvements arise from the synergistic interaction between global morphology, discriminative instance selection, and CNN-inspired local spatial inductive bias, rather than from a single architectural component."

Table 3. Ablation study of HRDPath model components: local window construction (LWC), with and without absolute positional encoding (APE).

Model	AUC	Sensitivity	Specificity
ViT++	0.710	0.385	0.813
DSMIL++	0.810	0.538	0.875
HRDPath-Multi-task (ViT + DSMIL)	0.769	0.615	0.938

HRDPath-Multi-task (ViT + DSMIL + LWC)	0.745	0.615	0.938
HRDPath-Multi-task (ViT + DSMIL + LWC + APE)	**0.846**	**0.692**	**0.938**

8. Figure 3 (morphological differences): The p-values should be corrected for multiple hypothesis testing.

Response:

All p-values associated with morphological analyses have been corrected using the Benjamini–Hochberg FDR procedure. Full statistical results are provided in Supplementary Table 9 (at the end of the document), and the Methods and figure captions have been updated accordingly.

Revised Figure 3 caption:

"P-values are calculated with the two-sided t-test and FDR-corrected, which are reported in Supplementary Table 9 and shown only if p-value < 0.05."

Revised Results (Manuscript line 249-273):

> "HRD+ HGSOC showed a higher proportion of neoplastic cells within tumor-associated regions compared with HRD– tumors. These differences were not due to variations in the overall neoplastic proportion between HRD+ and HRD- cancers, as no FDR-significant difference was observed when considering the entire WSI (Supplementary Figure 3a). At the nuclear level, HRD+ tumors were characterized by significantly increased nuclear roundness, reduced nuclear elongation and increased LCD ($p<0.05$; Figure 3b). HRD+ tumors also showed significantly reduced variability in neoplastic cell roundness and area ($p<0.05$, respectively; Figure 3c). To further characterize the magnitude of these associations, we evaluated effect sizes using Cohen's d by comparing feature distributions between HRD-positive and HRD-negative groups. Across evaluated features, effect sizes were generally small, consistent with the observed overlap in feature distributions between groups (Supplementary Table 9). Nonetheless, several features had absolute Pearson correlation greater than 0.15 with the predicted HRDScore, indicating consistent but modest correlations across patients ($p < 0.05$, Supplementary Table 10). Together, these results indicate that HRD status is associated with reproducible differences in nuclear morphology.
>
> Given the heterogeneity of HRD-associated genomic alterations, we next examined morphological differences across mutation-defined HRD subgroups. *BRCA1/2*-mutated tumors exhibited significantly increased nuclear roundness and reduced elongation, whereas these features were not significant in *BRCA1*-methylated cancers ($p<0.05$; Figure 3d–e). Other HRD+ tumors showed a significantly increased proportion of inflammatory cells, particularly within neoplastic regions (Supplementary Figure 3e–g). Significant reductions in both the mean and variability of nuclear area were observed in HRD+ tumors with

BRCA1/2-associated alterations, with the strongest effects detected in *BRCA1*-methylated cancers ($p<0.05$; Figure 3e–f). *BRCA1/2*-mutated tumors exhibited a significantly higher median LCD compared with HRD– tumors ($p<0.01$; Figure 3e). Overall, mutation-defined HRD subgroups display distinct but overlapping nuclear and immune microenvironmental features that remain significant after FDR correction."

Added Methods section (Manuscript line 644-653):

"**Statistical analysis of morphological features**

To compare morphological differences between HRD+ and HRD- tumors, we applied two-sample *t*-tests. Given the large number of morphological features evaluated, p-values were corrected for multiple hypothesis testing using the Benjamini–Hochberg false discovery rate (FDR) procedure. Statistical significance was assessed based on FDR-adjusted p-values unless otherwise stated.

In addition to statistical significance, effect sizes were explicitly quantified to account for the large sample sizes inherent to cell-level analyses. Cohen's *d* was reported for *t*-tests. These effect size measures were used to assess the magnitude of morphological differences independently of sample size. All statistical results, including raw p-values, FDR-adjusted p-values, and effect sizes, are provided in Supplementary Table 9."

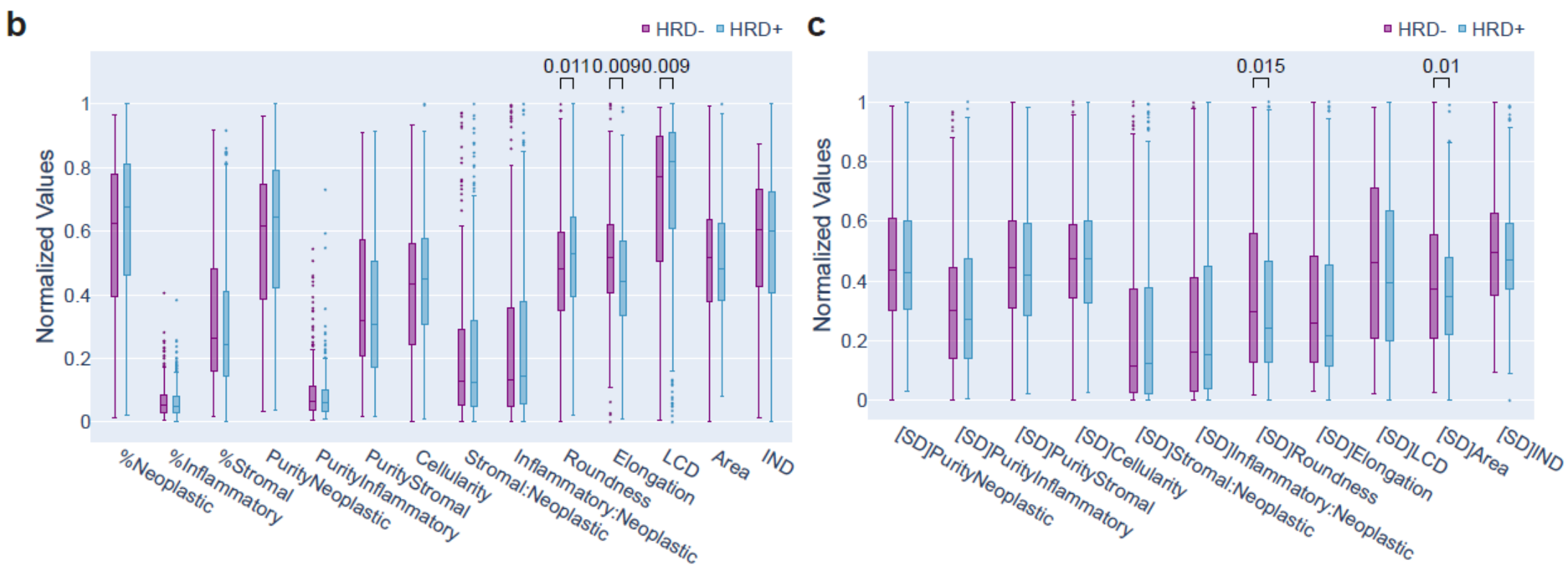

FDR-corrected Figure 3b-c.

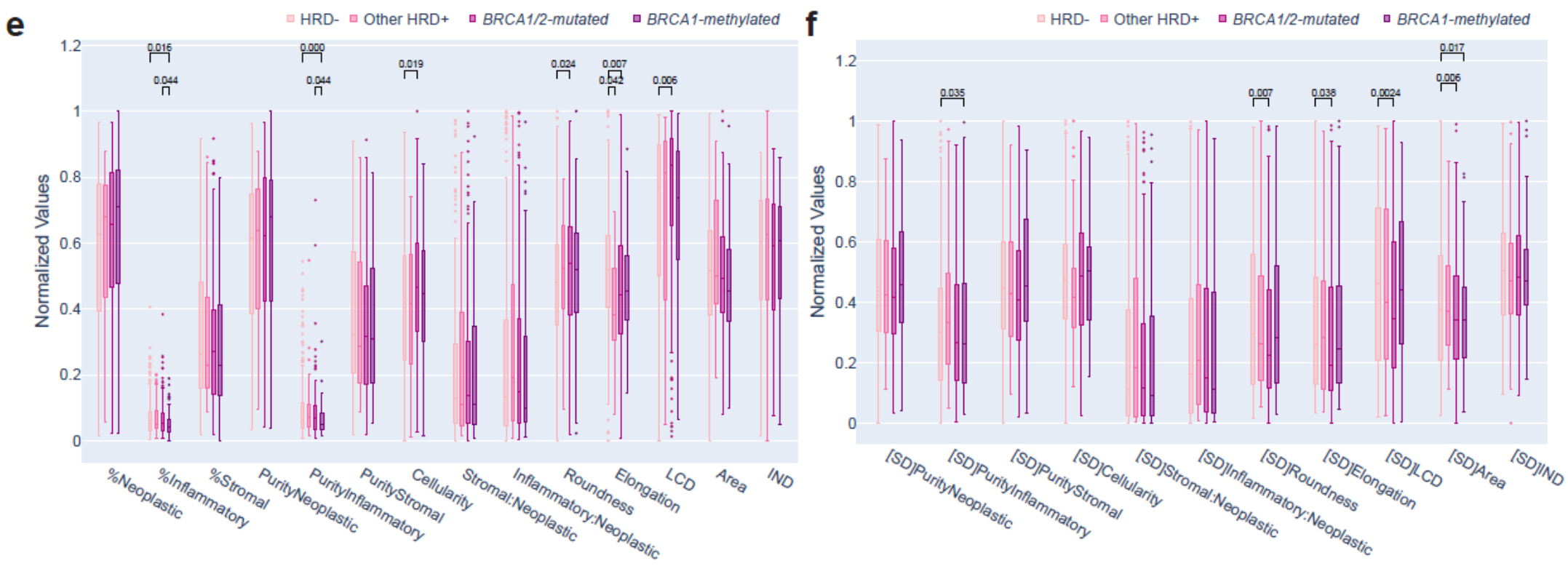

FDR-corrected Figure 3e-f.

9. Figure 3: The sample sizes (e.g. the number of cells) are very large, but the effect sizes are very small. How much of the total prediction power of HRDPath can be attributed to the analyzed morphological variables? The distributions (medians and boxes) of the variables look virtual identical in HRD+ and HRD- cancers. Thus, the classification power of each of the variables is expected to be very weak.

Response:

Although the effect size appears to be small, the pattern remains consistent and can be confirmed when considering the correlation with continuous HRDetect score (Supplementary Table 9). To quantify morphological features contribution, we performed a patient-level correlation analysis between the continuous HRDPath-predicted HRDScore and the FDR-significant morphology features. This analysis showed that several individual features were significantly associated with the predicted HRDScore (Pearson |r| >= 0.15 for many of the features, $p < 0.05$, Supplementary Table 10). To further quantify the predictive contribution of these features when combined, we trained linear Ridge regression models to predict HRDetect score and logistic regression models to classify HRD status under stratified 5-fold cross validation. Across both all-site and primary-only subsets, Ridge regression showed cross-validated R^2 values of −0.294 and HRD classification yielded AUCs of 0.593 (primary only) (Supplementary Table 11). This confirms that while morphology contribute to the predictivity of HRD, HRD cannot be predicted based on simple aggregated morphological features, and no single morphology variable is sufficient to explain or predict HRD status. It also highlights the power of HRDPath in integrating multiple morphological cues and their spatial context across whole-slide images. Accordingly, we have revised the Results and Discussion sections to clarify that the morphology analysis serves as a post hoc, descriptive assessment of individual associations, rather than a decomposition of the model's decision-making process.

Revised Results- manuscript line 259-261:

"Nonetheless, several features had absolute Pearson correlation greater than 0.15 with the predicted HRDScore, indicating consistent but modest correlations across patients ($p < 0.05$, Supplementary Table 10)."

Added Results- manuscript line 274-284:

"**Morphological feature associations and predictive contribution to HRD**

To gain better understanding of the contribution of our quantified morphological features to HRDPath predictions, we employed regression approach to evaluate the predictive contribution of aggregated single-cell morphological features. We used linear Ridge regression to predict HRDetect scores and logistic regression to classify HRD status, under stratified five-fold cross-validation. Across both all-site and primary-only subsets, Ridge regression yielded cross-validated R^2 values of −0.294. For HRD classification, morphology-only models achieved limited discrimination, with a maximum cross-validated AUC of 0.593 in the primary-only subset (Supplementary Table 11). Together, this confirms that while morphology contribute to the predictivity of HRD, HRD cannot be predicted based on simple aggregated morphological features, and no single morphology variable is sufficient to explain or predict HRD status. "

Revised Discussion- manuscript line 421-431:

"Analysis of morphological features contribution indicates that individual nuclear morphology features capture biologically-relevant aspects of HRD-associated tissue alterations. In particular, nuclei in HRD+ primary and metastatic cancers tended to exhibit reduced elongation compared with HRD- cases. Such alterations in nuclear morphology may reflect elevated proliferative activity associated with impaired DNA repair, as dividing cells often adopt a rounder morphology during mitosis [39]. Moreover, the high proliferation rate, as reflected by the higher neoplastic density, can disrupt epithelial polarity, as well as cell adhesion and cytoskeleton[40]. Our explainability pipeline can be potentially used to discover novel phenotypic differences but also advance our understanding of HRD alterations might shape the tumor microenvironment. Together, these findings indicate that individual morphology features capture biologically plausible aspects of HRD-related tissue alteration, while HRDPath integrates these signals within a broader spatial and contextual framework across whole-slide images."

Supplementary Table 9. See the end of the document.

Supplementary Table 10. Pearson correlation between individual morphology-derived features and the HRDPath-predicted HRDScore across anatomical subset (primary and all locations; significant features are marked in bold text).

Morphology feature	Subset	Pearson r	p-value
**Neoplastic elongation**	**Primary**	**−0.260**	**0.003**
Neoplastic roundness	Primary	0.136	0.125
**LCD**	**Primary**	**0.238**	**0.007**
**[SD] Neoplastic roundness**	**Primary**	**−0.206**	**0.019**
**[SD]Neoplastic area**	**Primary**	**−0.194**	**0.0228**
**Neoplastic elongation**	**All**	**−0.198**	**0.015**
Neoplastic roundness	All	0.052	0.530
**LCD**	**All**	**0.205**	**0.012**
[SD] Neoplastic roundness	All	−0.144	0.070
[SD]Neoplastic area	All	-0.148	0.070

Supplementary Table 11. Ridge and logistic regression analysis on all morphological features and HRDetect score and HRD status respectively across anatomical location subsets.

**Location**	**CV (Ridge)**<br>**HRDetect**	**CV AUC**<br>**HRD**
All sites	-0.294	0.539
**Primary only**	**−0.294**	**0.593**

10a. Figure 6 g/h (Kaplan-Meier analyses): Please add the information which of the cohorts is analyzed here. How were the patients treated?

Response:

Figures 6g and 6h are based on the integrated cohort combining UWOV and TCGAOV patients. Figure 6 captions have been revised to ensure clarity.

Revised Figure 6 caption:

g

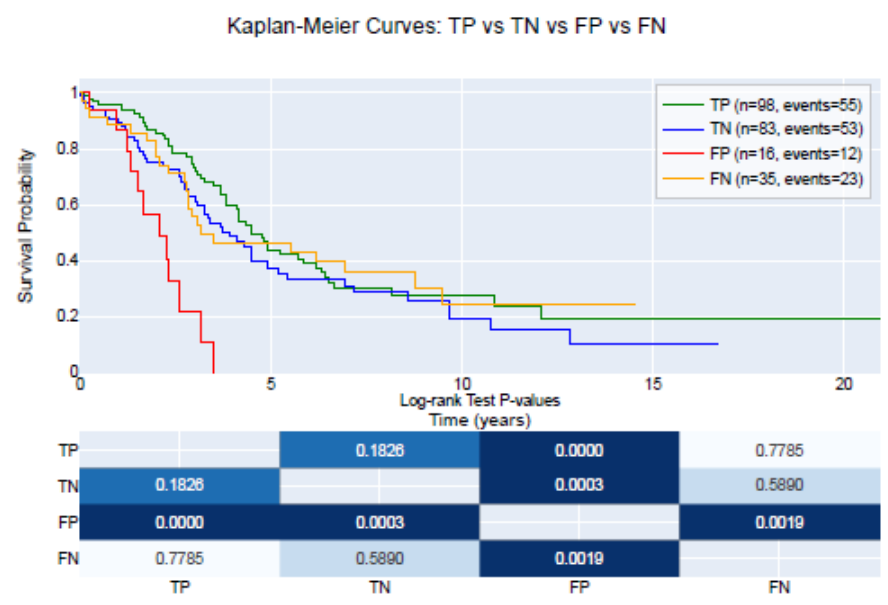

h

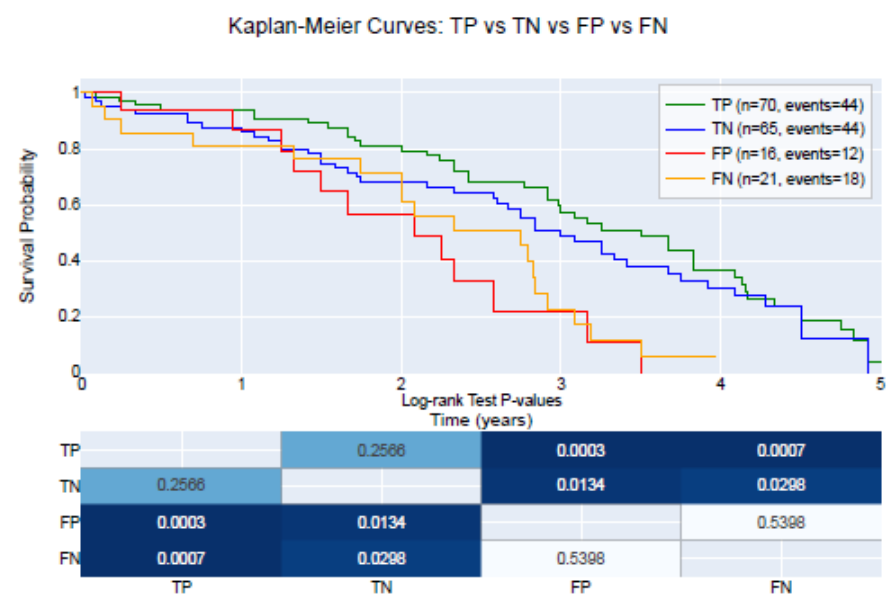

"**g-h.** Survival analyses were performed on the integrated UWOV and TCGAOV cohorts on overall survival (g) and patients with less than five years of survival (h)."

10b. In particular: Did the HRD+ patients receive PARR inhibitors? The results of the Kaplan-Meier analysis must be interpreted in the context of the therapies administered.

Response:

Patients in the UWOV cohort underwent primary debulking surgery followed by platinum-based chemotherapy and for most patients no PARP inhibitors were administered. However, we do not have detailed information on treatment received. We have now revised the discussion to include investigation of correlation between the groups identified in this analysis and their response to PARP inhibitors as interesting future direction.

Revised Results- manuscript line 347-355:

"Interestingly, false positive cases (HRDPath+/HRD-) were associated with significantly worse survival in the integrated UWOV and TCGA cohorts when considering all available follow-up ($p \leq 0.01$, Figure 6g). Moreover, the false negative cases (HRDPath-/HRD+) showed significantly worse survival than the true positives and true negatives when considering patients with less than 5 years of survival only ($p \leq 0.05$ and Figure 6h). Surprisingly, we do not observe a significant association between OS and HRDPath predictions (Supplementary Figure 5e) which could be due to limited patient number. This suggests that image-based predictions by HRDPath capture clinically meaningful phenotypes not reflected in genomic-based HRD assays, highlighting potential for broader prognostic relevance."

Revised Discussion- manuscript line 433-440:

"Intriguingly, misclassified cases, and especially HRDPath+/HRD-, were associated with significantly worse patient outcome, suggesting that image-derived morphological patterns may capture clinically relevant phenotypes not fully reflected by genomic HRD assays alone. Our current cohort involves heterogeneous treatments, where many patients were treated

before the frontline FDA approvals of PARP inhibitors for ovarian cancer in 2018[43]. An interesting direction in the future is to determine if these morphological signatures also correlate specifically with response to PARP inhibitor and to evaluate how morphological patterns can be leveraged to refine prognostic stratification in HGOSC.

11. The total objective function is a sum of a loss function for continuous prediction and a loss function for class prediction. How was the relative weighting of these two parts of the loss function chosen? Is this choice optimal for the classification problem under consideration?

Response:

The total loss was defined as an equal weighting sum of continuous and binary prediction losses. This choice was made to avoid introducing additional hyperparameters. We have clarified that no explicit loss-weight optimization was performed.

Revised Methods- manuscript line 545-547:

> "Equal weighting was adopted to avoid introducing additional hyperparameters and to encourage balanced learning across the two closely related tasks; no explicit optimization of loss weights was performed."

12. The conclusion (Discussion section) that the morphological differences between HRD+ and HRD- cancer could be attributed to the EMT is not supported very good by the data, as the effect sizes are very small.

Response:

We agree that that further experiments will be needed to confirm whether EMT could be particularly increased in HRD+ cancers lacking *BRCA1/2* associated alterations. The Discussion has been revised to frame this observation as potential explanation for our findings, and we explicitly state that further investigation is necessary to confirm it.

Revised Discussion (Manuscript line 429-433):

> "Interestingly, we identified a subset of HRD+ cancers that showed significantly increased elongation and reduced roundness, especially in cancers with no *BRCA1/2*-associated alterations. This might be associated with epithelial-to-mesenchymal transition which has been linked to differential DNA damage repair response[41,42]. However, further experimental validation is required to confirm the biological processes driving these differences…"

13. The authors conclude (end of Discussion section) that HRDPath has the potential to be

translated into a digital biomarker for HRD detection in ovarian cancer. Does HRDPath have the potential to be improved by being trained on larger data sets or in a different manner? The specificity of 94% could be sufficient for clinical utility, but a sensitivity of 69% appears much too low.

Response:

We agree that sensitivity is a critical consideration when identifying patients who may benefit from targeted therapies and lack of sensitivity assessment is major limitation of previous studies. Please refer to our response to reviewer 1 comment 1 where we propose strategies to improve model sensitivity and also discuss the need for larger patient cohort to progress the translational potential of HRDPath.

Supplementary Table 9. Merged statistical comparisons based on HRD status and mutation including FDR-corrected p-values and Cohen's d effect sizes. Values are reported to three decimal places.

	%]Neoplastic	% Inflam	% Stromal	Purity-Neoplastic	Purity- Inflam	Purity- Stromal	cellularity	Stromal:Neoplastic	Inflam:Neoplastic	SD-Purity-Neoplastic	SD-Purity-Inflam	SD-Purity-Stromal	SD-cellularity	SD-Stromal:Neoplastic	SD-Inflam:Neoplastic	Roundness	Elongation	LCD	Area	IND	SD-roundness	SD-Elongation	SD-LCD	SD-Area	SD-IND
**HRD+ vs HRD-**																									
**Stat**	2.131	-0.851	-1.788	1.917	-1.983	-1.921	2.154	-0.244	-0.948	-0.061	-1.984	-0.147	-0.45	0.673	0.139	3.136	-3.46	3.404	0.266	-0.753	-2.988	-1.679	-2.007	-3.261	-1.977
**P**	0.033	0.395	0.074	0.056	0.048	0.055	0.032	0.807	0.344	0.952	0.048	0.883	0.653	0.501	0.889	0.002	0.001	0.001	0.79	0.452	0.003	0.094	0.045	0.001	0.048
**FDR**	0.107	0.581	0.133	0.107	0.107	0.107	0.107	0.918	0.537	0.952	0.107	0.926	0.816	0.659	0.926	0.011	0.009	0.009	0.918	0.627	0.015	0.156	0.107	0.01	0.107
**Cohen_d**	0.16	-0.064	-0.134	0.144	-0.149	-0.144	0.162	-0.018	-0.071	-0.005	-0.15	-0.011	-0.034	0.05	0.01	0.236	-0.262	0.257	0.02	-0.056	-0.225	-0.127	-0.151	-0.247	-0.149
**Other HRD+ vs HRD-**																									
**stat**	0.537	0.567	-0.539	0.555	-0.024	-0.75	-0.444	-1.13	-2.271	0.435	-0.107	0.203	-1.795	-0.243	-0.782	2.492	-3.2	2.145	2.145	-0.356	-0.961	0.055	-0.348	-0.239	-1.351
**P**	0.592	0.572	0.591	0.58	0.981	0.455	0.658	0.261	0.024	0.665	0.915	0.839	0.076	0.808	0.435	0.014	0.002	0.034	0.034	0.723	0.339	0.956	0.729	0.812	0.18
**FDR**	0.954	0.954	0.954	0.954	0.981	0.954	0.954	0.815	0.172	0.954	0.981	0.954	0.315	0.954	0.954	0.172	0.042	0.172	0.172	0.954	0.94	0.981	0.954	0.954	0.642
**Cohen_d**	0.068	0.071	-0.069	0.07	-0.003	-0.096	-0.056	-0.119	-0.194	0.056	-0.013	0.027	-0.224	-0.03	-0.077	0.269	-0.316	0.231	0.284	-0.052	-0.112	0.008	-0.046	-0.029	-0.164
***BRCA1/2*-Mutated vs_HRD-**																									
**stat**	1.771	0.125	-1.624	1.506	-0.986	-1.81	2.912	0.01	-0.137	-0.734	-1.568	-1.138	0.247	0.34	0.59	2.743	-3.261	3.534	0.141	-1.418	-3.351	-2.517	-2.72	-3.495	-1.776
**P**	0.077	0.901	0.105	0.133	0.325	0.071	0.004	0.992	0.891	0.463	0.117	0.256	0.805	0.734	0.555	0.006	0.001	0.0	0.888	0.157	0.001	0.012	0.007	0.001	0.076

**FDR**	0.176	0.938	0.219	0.237	0.477	0.176	0.019	0.992	0.938	0.644	0.226	0.399	0.938	0.917	0.731	0.024	0.007	0.006	0.938	0.262	0.007	0.038	0.024	0.006	0.176
**Cohen_d**	0.155	0.011	-0.141	0.133	-0.087	-0.158	0.255	0.001	-0.012	-0.065	-0.133	-0.102	0.022	0.032	0.052	0.242	-0.26	0.293	0.012	-0.128	-0.279	-0.189	-0.239	-0.283	-0.15
***BRCA1*-Methylated vs HRD-**																									
**stat**	2.019	-3.253	-1.463	1.83	-4.532	-1.319	1.124	-0.035	-1.031	0.726	-2.799	1.261	-0.022	1.135	-0.189	1.653	-1.846	1.661	-1.388	0.561	-1.656	-1.214	-0.428	-3.113	-1.218
**P**	0.045	0.001	0.145	0.069	0.0	0.189	0.263	0.972	0.304	0.469	0.006	0.209	0.982	0.258	0.85	0.1	0.066	0.098	0.167	0.576	0.099	0.226	0.669	0.002	0.225
**FDR**	0.226	0.016	0.33	0.247	0.0	0.353	0.365	0.982	0.4	0.586	0.035	0.353	0.982	0.365	0.924	0.25	0.247	0.25	0.347	0.685	0.25	0.353	0.76	0.017	0.353
**Cohen_d**	0.225	-0.301	-0.159	0.208	-0.365	-0.146	0.123	-0.004	-0.118	0.084	-0.251	0.152	-0.002	0.142	-0.022	0.177	-0.162	0.165	-0.138	0.057	-0.165	-0.103	-0.047	-0.277	-0.126
***BRCA1/2*-Mutated vs Other HRD+**																									
**stat**	0.651	-0.442	-0.524	0.481	-0.634	-0.445	2.343	0.943	1.724	-0.892	-0.96	-0.907	1.867	0.46	1.21	-0.156	0.756	0.469	-1.988	-0.481	-1.315	-1.095	-1.373	-1.952	0.156
**P**	0.516	0.659	0.601	0.631	0.528	0.657	0.021	0.347	0.086	0.374	0.339	0.366	0.064	0.646	0.228	0.876	0.451	0.64	0.049	0.631	0.191	0.277	0.173	0.054	0.876
**FDR**	0.716	0.716	0.716	0.716	0.716	0.716	0.402	0.716	0.43	0.716	0.716	0.716	0.402	0.716	0.712	0.876	0.716	0.716	0.402	0.716	0.683	0.716	0.683	0.402	0.876
**Cohen_d**	0.09	-0.058	-0.074	0.065	-0.087	-0.063	0.323	0.102	0.172	-0.121	-0.134	-0.126	0.256	0.057	0.139	-0.019	0.104	0.064	-0.292	-0.071	-0.188	-0.223	-0.196	-0.303	0.022
***BRCA1*-Methylated vs Other HRD+**																									
**stat**	1.055	-2.709	-0.593	0.926	-2.717	-0.331	1.208	0.807	0.544	0.187	-1.837	0.766	1.574	1.097	0.386	-0.678	1.525	-0.539	-2.883	0.665	-0.384	-0.643	-0.005	-1.869	0.288
**P**	0.293	0.008	0.554	0.356	0.008	0.741	0.229	0.421	0.588	0.852	0.069	0.445	0.118	0.274	0.7	0.498	0.13	0.591	0.005	0.507	0.702	0.522	0.996	0.064	0.774
**FDR**	0.733	0.065	0.777	0.777	0.065	0.841	0.715	0.777	0.777	0.888	0.344	0.777	0.463	0.733	0.835	0.777	0.463	0.777	0.065	0.777	0.835	0.777	0.996	0.344	0.841
**Cohen_d**	0.165	-0.448	-0.094	0.143	-0.47	-0.052	0.19	0.119	0.077	0.029	-0.303	0.119	0.251	0.165	0.056	-0.103	0.241	-0.084	-0.473	0.111	-0.061	-0.114	-0.001	-0.311	0.046
***BRCA1*-Methylated vs *BRCA1/2*-Mutated**																									

**stat**	0.606	-2.947	-0.159	0.62	-2.941	0.082	-1.135	-0.037	-0.824	1.197	-1.233	1.944	-0.219	0.786	-0.583	-0.599	1.062	-1.176	-1.42	1.672	1.053	0.993	1.62	-0.025	0.195
**P**	0.545	0.003	0.874	0.536	0.004	0.935	0.258	0.97	0.411	0.233	0.219	0.053	0.827	0.433	0.561	0.55	0.289	0.241	0.157	0.096	0.293	0.322	0.107	0.98	0.846
**FDR**	0.738	0.044	0.98	0.738	0.044	0.98	0.611	0.98	0.722	0.611	0.611	0.445	0.98	0.722	0.738	0.738	0.611	0.611	0.611	0.534	0.611	0.62	0.534	0.98	0.98
**Cohen_d**	0.075	-0.317	-0.019	0.077	-0.297	0.01	-0.138	-0.004	-0.098	0.147	-0.138	0.244	-0.026	0.097	-0.074	-0.071	0.127	-0.145	-0.166	0.188	0.129	0.129	0.198	-0.003	0.024

Remarks to reviewers

Reviewer #2:

The authors have addressed most of my comment, but two concerns remain:

1. Related to my original concern 2 and Suppl. Fig 2:
The AUC curves displayed appear as two straight lines with different slopes that are connected at the sensitivity-specificity point reported in Table 2. Thus, the ROC curves and probably (and the reported AUCs) seem to belong to a binary classification. However, it would be important and interesting to see the AUC curves for the continuous HRD score. Starting from this, trade-off between sensitivity and specificity and an optimization of the trade-off according to the clinical needs can be discussed.

Response:

Thank you for raising this point. For the sensitivity and specificity trade off relevant to the manuscript's primary endpoint of HRD classification, the most appropriate ROC analysis is based on predicted HRD probability versus binary HRD status. We have corrected the ROC computation in the revised analysis so that curves are now calculated across all unique thresholds rather than a fixed threshold (Supplementary Figure 2a-i).

We also generated ROC curves for HRDScores (Figure R1 -provided for reviewer attention). However, we believe that the ROC curves based solely on the HRDScore do not fully reflect HRDPath performance as it is a multi-task model. Beyond the ability of the predicted score to separate binary HRD status, a critical consideration is its clinical interpretability and its behavior as a biologically meaningful continuous approximation of molecular HRD score. For this reason, we examined the distribution of the ground truth HRDScore (Figure R1i) and the correlation between the predicted histopathological HRDScore and the molecular HRDScore (Supplementary Figure 2j-l).

These analyses suggest that the molecular HRDScore is itself nonuniformly distributed, which may partly explain the more stepwise appearance of score-based ROC curves in multi-task models. More importantly, we observed that concordance between predicted and molecular HRDScore improves substantially when restricted to cases with a greater number of primary tumor slides (See Response to Comment 2). This pattern is consistent with other analyses and suggests that the clinical feasibility of HRDPath is closely linked to tissue representation. In practical terms, it indicates that performance can be materially improved when more primary tumour slides are available, supporting a deployment strategy in which the number of primary slides is treated as an important quality control factor.

Accordingly, we interpret the probability-based ROC as the most relevant analysis for classification trade off, while the score distribution and slide stratified concordance analyses provide the more informative evidence for the clinical feasibility of the

continuous HRDScore output.

We have updated the results and analysed the trade-off between sensitivity and specificity and how this can be adjusted according to the clinical need as follows:

Revised Results- manuscript line 127-137:

> "To evaluate the inherent discriminative capacity of the continuous HRD probabilities, AUROC analysis was performed (Supplementary Figure 2a-i). HRDPath maintained robust overall ranking ability across the continuous score spectrum. Notably, the ROC curve demonstrates a favorable trade-off pattern in the high-specificity regime, achieving a True Positive Rate of approximately 0.7-0.8 while maintaining a False Positive Rate below 0.2. However, pushing the model to maximum sensitivity requires a substantial compromise in specificity, a pattern consistent with the score dispersion observed among certain HRDetect positive cases (Supplementary Figure 2j). These continuous probability metrics confirm that while the fixed 0.5 threshold provides a balanced baseline, the continuous scores capture a meaningful clinical signal that could support threshold optimization for specific sensitivity-driven or specificity-driven clinical objectives."

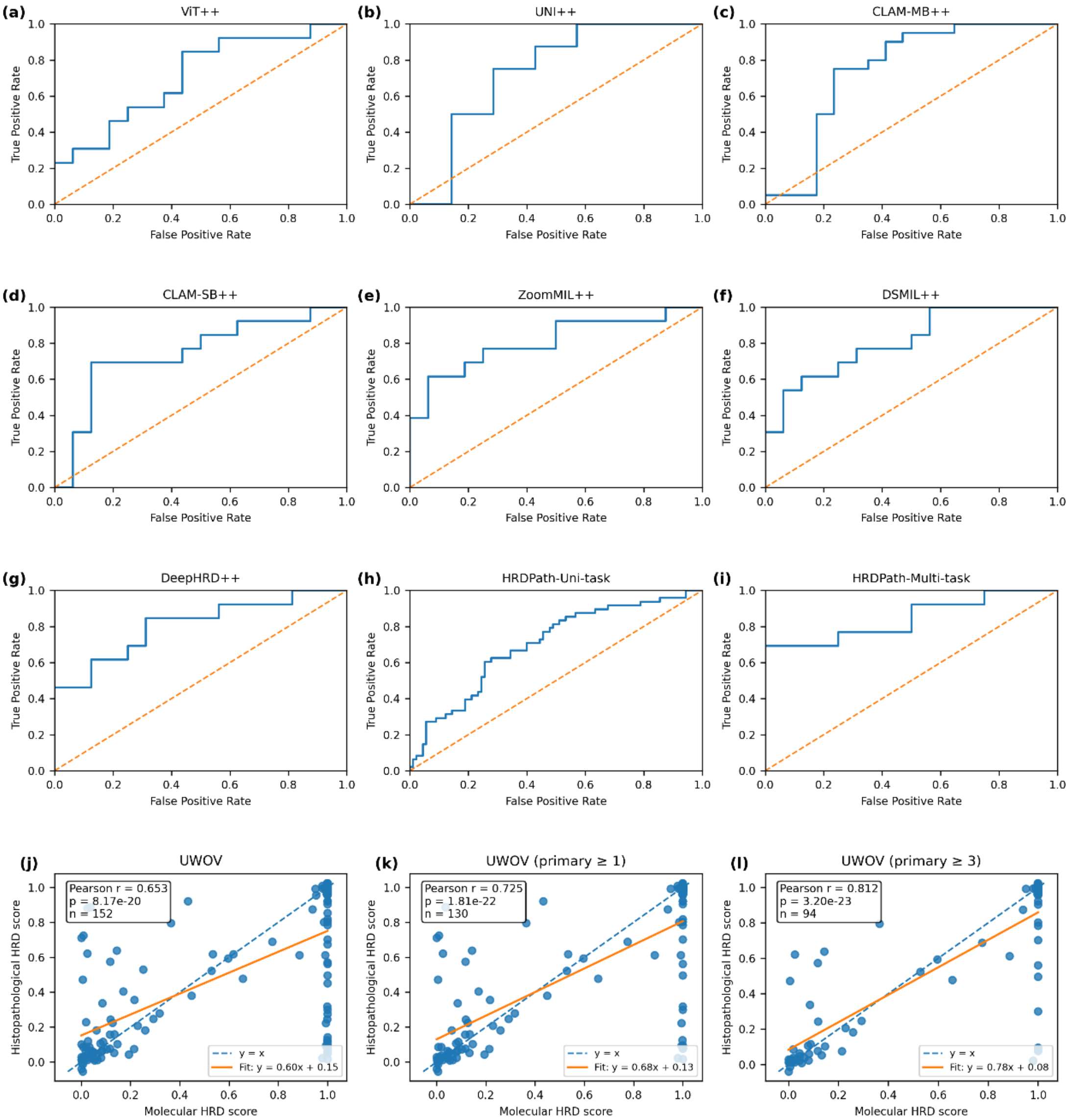

Supplementary Figure 2. Receiver operating characteristic (ROC) curves and score correlation analyses across cohorts. (a–i) show ROC curves for the nine models evaluated on the UWOV test subset, corresponding to the models reported in Table 2. Each subplot reports the area under the curve (AUC). The binary HRD low/high classification metrics in Table 2 were computed using a fixed probability threshold of 0.5 for all models, without cohort-specific or post hoc threshold optimisation. The ROC curves therefore illustrate the full sensitivity–specificity trade-off across all possible operating points for each model's predicted HRD probability. **(j-l)** Scatterplot comparing predicted histopathological HRD scores by HRDPath against ground truth molecular HRD scores based on UWOVHRD, varied by the subsets defined by the number of available primary slides (n_primary) per patient when all slides considered (j), n_primary ≥ 1 (k), and n_primary ≥ 3 (l). Correlation coefficients for each nested subset are reported in the legend. Dashed lines indicate the identity line (y = x), and the solid orange line indicates the overall linear regression fit.

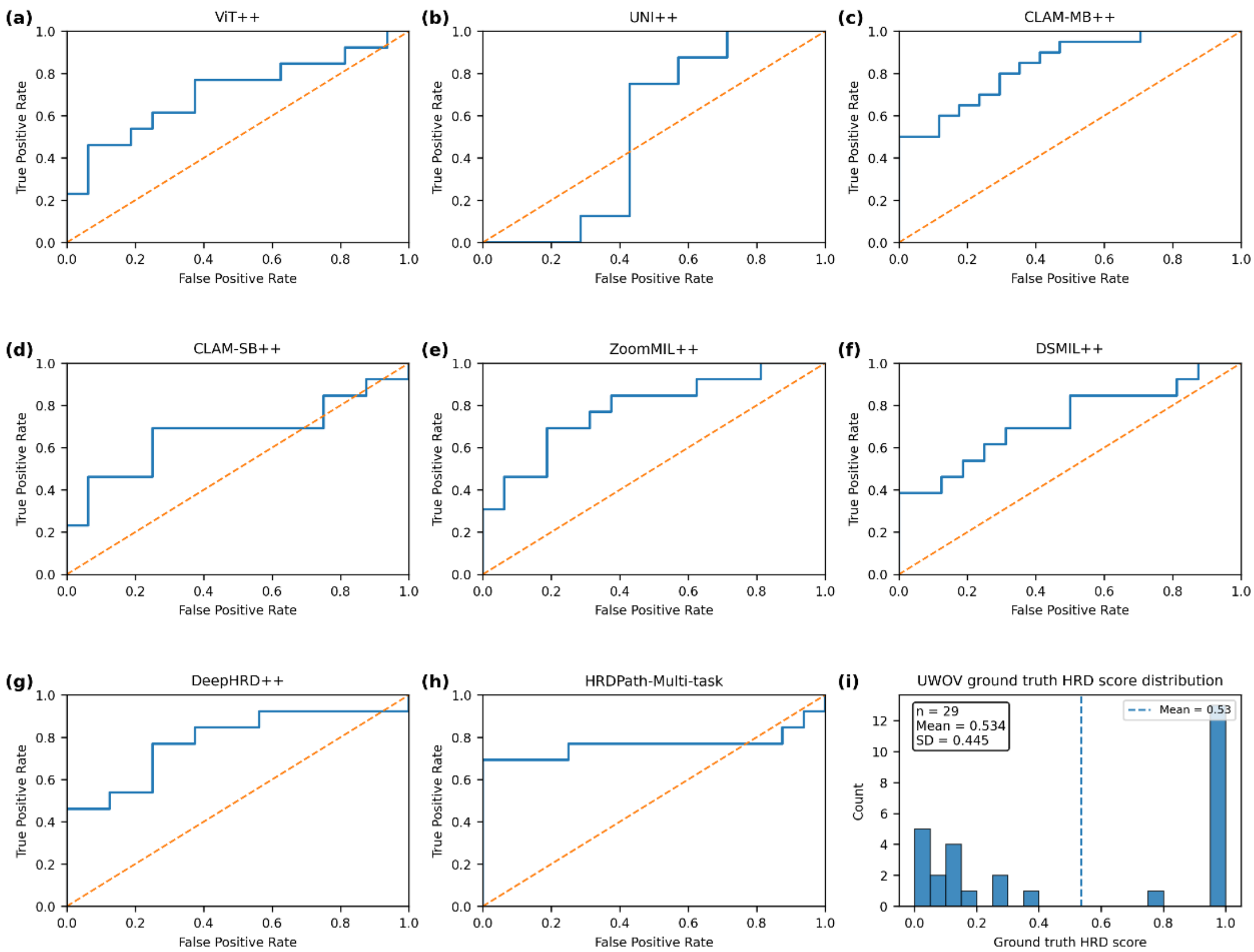

Figure R1 (For reviewer attention). Receiver operating characteristic (ROC) curves of predicted HRDScore to HRD status. (a-h) show ROC curves for eight models evaluated on the UWOV test subset with HRDScore predictions. (i) shows the distribution of UWOV HRDScore distributions.

2. Related to my original concern 4: "The correlations between the histopathological HRD score and the molecular HRD score should be shown in scatterplots for each of the cohorts. Such displays will provide additional insights: whether disagreements between the two scores occur for tumors close to the thresholds or if some of the tumors are having clearly high molecular HRD scores while having clearly low histopathological HRD scores or vice versa."

Response:

We have now added scatterplots for the UWOV cohort in Supplementary Figure2 j-k, comparing the continuous histopathological HRD scores predicted by HRDPath with the corresponding molecular HRD reference scores. These plots complement the correlation coefficients by enabling direct visual assessment of concordance, dispersion, and discordant cases across the full score range.

Revised Results- manuscript Line 164-168:

> "… Interestingly, we found that Pearson correlation between continuous HRD

score and HRDPath predicted score increased from 0.65 to 0.81 when more primary slides were considered (Supplementary Figure 2k-l), suggesting that this approach might be more appropriate to mitigate tumor heterogeneity. This pattern remained consistent across the entire HRD score range, with no specific HRD score values linked to discordant cases.”

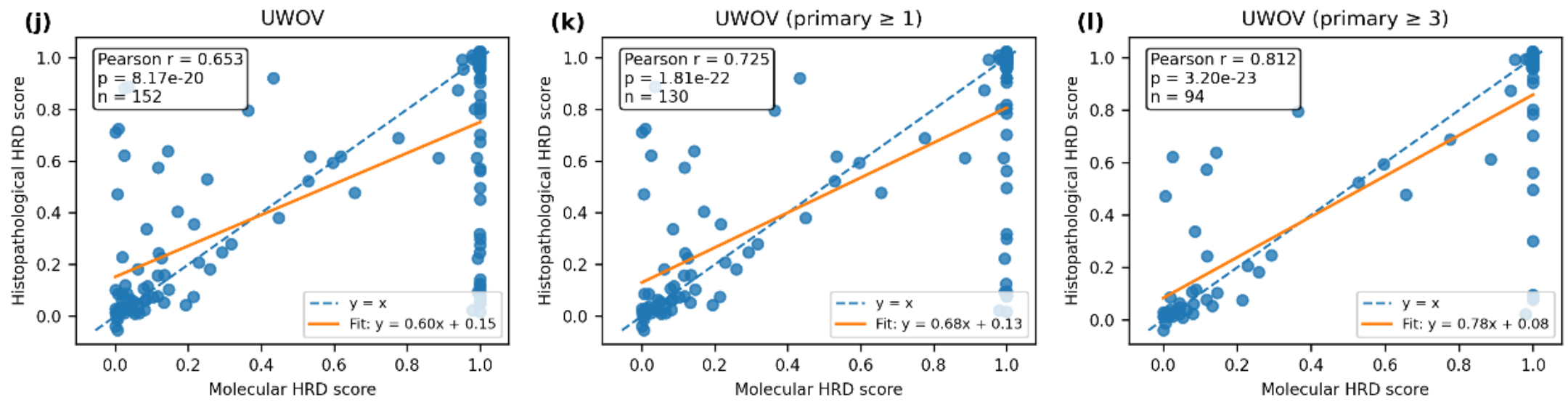

Supplementary Figure 2… (j-l) Scatterplot comparing predicted histopathological HRD scores by HRDPath against ground truth molecular HRD scores based on UWOVHRD, varied by the subsets defined by the number of available primary slides (n_primary) per patient when all slides considered (j), n_primary ≥ 1 (k), and n_primary ≥ 3 (l). Correlation coefficients for each nested subset are reported in the legend. Dashed lines indicate the identity line (y = x), and the solid orange line indicates the overall linear regression fit.
